# Sublethal executioner caspase activation in hepatocytes promotes liver regeneration through the JAK/STAT3 pathway

**DOI:** 10.1371/journal.pbio.3003357

**Published:** 2025-08-28

**Authors:** Zhiyuan Cao, Lining Qin, Kaixuan Liu, Chen Yao, Enhong Li, Xiaoyu Hao, Molin Wang, Baichun Jiang, Yongxin Zou, Huili Hu, Qiao Liu, Changshun Shao, Yaoqin Gong, Gongping Sun

**Affiliations:** 1 The Key Laboratory of Experimental Teratology of the Ministry of Education, State Key Laboratory of Reproductive Medicine and Offspring Health, and Department of Histology and Embryology, School of Basic Medical Sciences, Cheeloo College of Medicine, Shandong University, Jinan, China; 2 The Key Laboratory of Experimental Teratology of the Ministry of Education, State Key Laboratory of Reproductive Medicine and Offspring Health, and Department of Genetics, School of Basic Medical Sciences, Cheeloo College of Medicine, Shandong University, Jinan, China; 3 Department of Systems Biomedicine and Research Center of Stem Cell and Regenerative Medicine, School of Basic Medical Sciences, Cheeloo College of Medicine, Shandong University, Jinan, Shandong, China; 4 State Key Laboratory of Radiation Medicine and Protection, Institutes for Translational Medicine, Soochow University Suzhou Medical College, Suzhou, Jiangsu, China; The University of Edinburgh School of Biological Sciences, UNITED KINGDOM OF GREAT BRITAIN AND NORTHERN IRELAND

## Abstract

Apoptosis has been reported to drive regeneration in many species. Executioner caspases, the key effectors in apoptosis, are responsible for production and secretion of various pro-regenerative signals from apoptotic cells to the surrounding cells. However, whether executioner caspase activation (ECA) can promote regeneration without inducing apoptosis is poorly understood. Here, by generating transgenic mice carrying a lineage tracing system for cells that have experienced ECA, we demonstrate that ECA occurs in a few hepatocytes in homeostatic livers. The fraction of hepatocytes with ECA dramatically expands during regeneration after partial hepatectomy (PHx) or carbon tetrachloride (CCl_4_) treatment. Interestingly, rather than undergoing apoptosis, the majority of hepatocytes with ECA survive and proliferate during liver regeneration. Inhibition of ECA in livers results in reduced hepatocyte proliferation and impaired regeneration, whereas increasing ECA to a level sufficient to kill hepatocytes also impedes regeneration, suggesting that ECA needs to be precisely controlled at a sublethal level. Mechanistically, we show that ECA promotes hepatocyte proliferation through enhancing JAK/STAT3 activity. Our work reveals an essential apoptosis-independent role of executioner caspases in liver regeneration.

## Abstract 2

Besides their critical roles in apoptosis, executioner caspases also have non-apoptotic functions. This study shows that sublethal executioner caspase activation enhances hepatocyte proliferation via JAK/STAT3 signaling, revealing an apoptosis-independent role for executioner caspases in promoting liver regeneration.

## Introduction

Apoptosis is a conserved cell death program critical in organ development and homeostasis maintenance. Executioner caspases, including caspase-3 and caspase-7 in mammals, are key apoptosis effectors whose activation dismantles cells into apoptotic bodies [1,2]. Besides their role in apoptosis, executioner caspases also have non-apoptotic functions [3]. For example, executioner caspases can regulate the growth of *Drosophila* wing discs [4], proliferation of cells in mouse sebaceous glands [5], cell fate specification [6], oncogenic transformation [7–10], and aggressiveness of cancer cells [11].

Executioner caspases have emerged as critical regulators in regeneration across diverse species including Hydra [12], *Drosophila* [13], zebrafish [14], Xenopus [15], salamander [16], and mouse [17]. Upon injury, activated executioner caspases in apoptotic cells can cleave multiple protein substrates, leading to the production and secretion of molecules like Wnt3 [18], PGE2 [17], EGFR ligands [19], ATP [20] to promote survival and proliferation of the neighboring cells. Executioner caspase activation (ECA) also causes formation of apoptotic extracellular vesicles [21–23], which can be engulfed by the neighboring cells or immune cells to initiate regenerative processes [24–26]. In these cases, the cells with ECA die, and the regenerative proliferation is conducted by other cells in the tissue or organ. Recently, studies on salamander and *Drosophila* have unveiled the involvement of living cells with ECA in regeneration. Wang and colleagues reported the existence of myofibers with active caspase-3, but no TUNEL staining in regenerating salamander limbs after amputation. Inhibition of caspase activity by XIAP overexpression impaired dedifferentiation of myofibers after amputation [16]. Previously, using CasExpress, a lineage tracing system for cells that have experienced ECA, our group demonstrated that after X-ray radiation or transient overexpression of pro-apoptotic genes, a large group of cells in *Drosophila* wing imaginal discs can survive from ECA, proliferate, and participate in formation of regenerated discs [27]. However, whether and how executioner caspases promote regeneration without inducing apoptosis in mammals are unclear.

The liver exhibits remarkable regenerative capacity among adult mammalian organs. After partial hepatectomy (PHx) or acute chemical injury, livers can restore the original weight and function by triggering proliferation of hepatocytes [28]. To investigate the role of ECA in liver regeneration, we generated transgenic mice carrying mCasExpress reporter, the mammalian version of the CasExpress lineage tracing system. Using these mice, we demonstrate that hepatocytes with ECA are dramatically increased in regenerating livers. Strikingly, we show that instead of committing apoptosis and triggering apoptosis-induced regeneration, hepatocytes with ECA must survive to ensure robust hepatocyte proliferation and efficient liver regeneration. Mechanistically, we show that ECA promotes hepatocyte proliferation through increasing JAK-STAT3 activity.

## Results

### mCasExpress reporter reveals a few cells with ECA in the homeostatic liver

**Fig 1:**
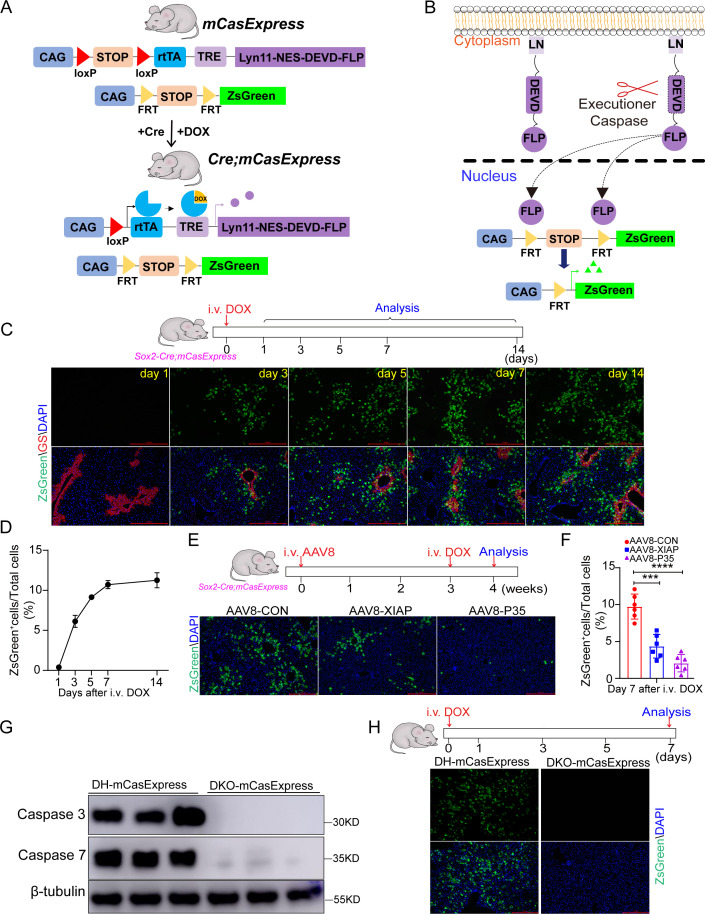
mCasExpress reporter reveals a few cells with ECA in the homeostatic liver. **(A)** The schematic of the mating strategy to generate *Cre; mCasExpress* mice. **(B)** The schematic of mCasExpress. **(C)** The representative images of livers at the indicated time points after injection of DOX. The staining for glutamine synthase (GS) marks the central veins. Scale bar: 100 μm. **(D)** Quantification of the percentage of ZsGreen^+^ cells in all cells within the field. For each time point, 3 mice were included and 3 fields per mouse were quantified. **(E)** The representative images showing the effect of overexpressing XIAP or p35 on expression of ZsGreen in livers. Scale bar, 100 μm. **(F)** Quantification of the percentage of ZsGreen^+^ cells in livers from the indicated groups. Six mice per group and 3 fields per mouse. **(G)** Western blotting shows loss of caspase-3 and caspase-7 proteins in livers from *Alb-Cre*; *mCasExpress*; *Casp3*^*flox/flox*^; *Casp7*^*flox/flox*^ (DKO-mCasExpress) mice. Livers from the heterozygotes (*Alb-Cre*; *mCasExpress*; *Casp3*^*flox/+*^; *Casp7*^*flox/+*^, labeled as DH-mCasExpress) were used as control. **(H)** No ZsGreen expression in DKO-mCasExpress livers after DOX injection. Scale bar: 100 μm. Data are presented as the mean ± SD. ***: *P* < 0.001. ****: *P* < 0.0001. i.v.: intravenous injection. The data underlying the graphs shown in the figure can be found in S1 Data. Raw blot images can be found in S1 Raw Images.

To label cells that experience ECA in mice, we generated a transgenic mouse line carrying both *CAG-loxP-STOP-loxP-rtTA* (*LSL-rtTA*) and *TRE-Lyn11-NES-DEVD-FLP* and a transgenic mouse line carrying *CAG-FRT-STOP-FRT-ZsGreen* (*FSF-ZsGreen*). By crossing these two lines, we obtained *LSL-rtTA; TRE-Lyn11-NES-DEVD-FLP; FSF-ZsGreen* mice, which were designated as *mCasExpress* mice. In *mCasExpress* mice, a fusion protein containing Lyn11 sequence, nuclear export signal (NES), an executioner caspase-specific cleavage site DEVD, and a DNA recombinase FLP (LN-DEVD-FLP) is expressed in a Cre- and doxycycline (DOX)-dependent manner (Fig 1A). Without executioner caspase activity, FLP is tethered to the cell membrane. Once executioner caspases are activated, FLP is released from the membrane and translocate into the nucleus to remove the transcriptional termination signal (STOP) between the two FRT sites, leading to expression of the green fluorescent protein ZsGreen (Fig 1B). We crossed *mCasExpress* mice with *Sox2-Cre* mice, in which Cre is expressed in all the epiblast-derived tissues, to generate *Sox2-Cre; mCasExpress* mice. Both *mCasExpress* mice and *Sox2-Cre; mCasExpress* mice exhibited normal appearance, body weight, liver weight, liver histology, serum aspartate transaminase (AST), and serum alanine transaminase (ALT) (S1 Fig).

Without DOX, LN-DEVD-FLP was rarely expressed and *Sox2-Cre; mCasExpress* mice displayed no ZsGreen expression in the liver (S2A and S2B Fig). Transcription of *LN-DEVD-FLP* was highly induced after injection of 5 mg/kg DOX through tail veins and diminished within 7 days after injection (S2A Fig). We assessed ZsGreen expression in the livers from *Sox2-Cre; mCasExpress* mice after injection of DOX. Few ZsGreen^+^ cells were detected on day 1 after injection (Fig 1C and 1D), possibly because one day was insufficient for cells to accumulate a detectable level of ZsGreen protein. The percentage of ZsGreen^+^ cells increased to 6% on day 3 after injection and 10.7% on day 7 (Fig 1C and 1D). From day 7 to day 14, the percentage of ZsGreen^+^ cells stayed constant (Fig 1C and 1D), in consistence with the diminished LN-DEVD-FLP expression (S2A Fig). *mCasExpress* mice or *Sox2-Cre; FSF-ZsGreen* mice contained no ZsGreen^+^ cells in livers after DOX injection (S2C Fig), supporting the requirement of FLP activity in ZsGreen expression. To validate that mCasExpress specifically detects ECA, we performed a series of experiments. First, we generated mice carrying *LSL-rtTA* and *TRE:Lyn11-NES-DEVA-FLP*, in which the executioner caspase cleavage site was mutated, and crossed these mice with *FSF-ZsGreen* mice and *Sox2-Cre* mice to obtain *Sox2-Cre; mCasExpress*^*mut*^ mice (S3A Fig). Little ZsGreen^+^ cells were detected in *Sox2-Cre; mCasExpress*^*mut*^ livers after DOX injection (S3B Fig), confirming that expression of ZsGreen in *Sox2-Cre; mCasExpress* mice requires cleavage of the DEVD site. Next, we delivered adeno-associated virus serotype 8 (AAV8) expressing baculovirus p35 or mouse XIAP, which are inhibitors of caspases [29], to *Sox2-Cre; mCasExpress* mice. Overexpression of either p35 or XIAP did not affect the liver-to-body weight ratio and serum ALT/AST levels (S4A–S4C Fig), but dramatically reduced the percentage of ZsGreen^+^ cells in livers (Fig 1E and 1F), indicating that ZsGreen^+^ cells depend on caspase activation. p35 and XIAP suppress the activity of both initiator caspases and executioner caspases in mice. To determine whether expression of ZsGreen relies on executioner caspases, we generated liver-specific *Casp3* and *Casp7* double knockout mice (*Alb-Cre; Casp3*^*flox/flox*^*; Casp7*^*flox/flox*^, referred to as DKO). Knockout of both *Casp3* and *Casp7* did not affect liver weight and function (S4D–S4F Fig). We crossed mCasExpress into DKO background to get *Alb-Cre; mCasExpress; Casp3*^*flox/flox*^*; Casp7*^*flox/flox*^ (DKO-mCasExpress) mice. While livers heterozygous in both *Casp3* and *Casp7* (*Alb-Cre; mCasExpress; Casp3*^*flox/+*^*; Casp7*^*flox/+*^, referred to as DH-mCasExpress) contained ZsGreen^+^ cells on day 7 after DOX injection, loss of caspase-3 and caspase-7 abolished expression of ZsGreen (Fig 1G and 1H). All these data together demonstrate that ZsGreen^+^ cells in homeostatic livers are cells with ECA.

### ECA occurs preferentially in the pericentral hepatocytes of the homeostatic liver

**Fig 2:**
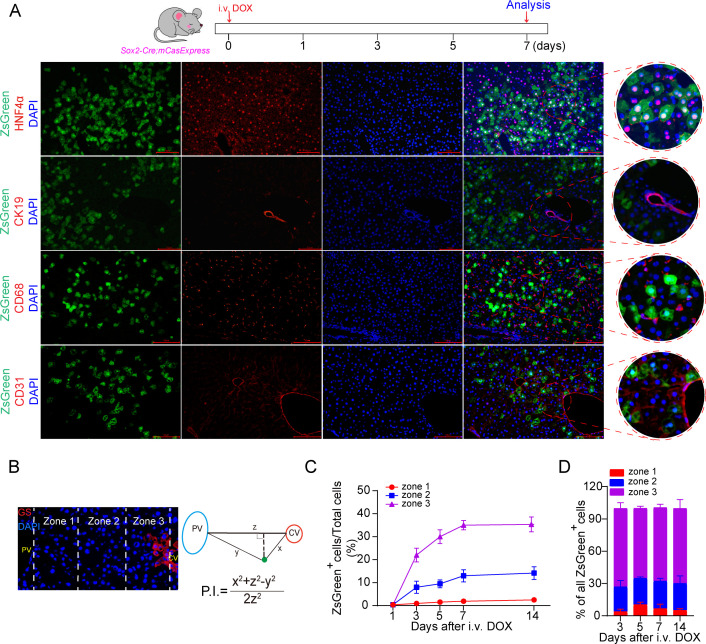
ECA occurs preferentially in pericentral hepatocytes of homeostatic livers. **(A)** The representative images of livers with staining of the hepatocyte marker HNF4α, the cholangiocyte marker CK19, the Kupffer cell marker CD68 or the endothelial cell marker CD31. Scale bar: 50 μm. **(B)** The zonation method. PV: portal vein. CV: central vein. P.I.: position index. Zone 1: P.I. ≥ 0.66. Zone 2: 0.33 ≤ P.I. < 0.66. Zone 3: P.I. < 0.33. **(C)** Quantification of the percentage of ZsGreen^+^ cells in total cells of each zone at the indicated time points. Three mice per group and 3 fields per mouse. **(D)** The bar graph shows the ratio of the number of ZsGreen^+^ cells in zone 1, 2, or 3 to the number of all ZsGreen^+^ cells in the quantified field. Three mice per group and 3 fields per mouse. i.v.: intravenous injection. The data underlying the graphs shown in the figure can be found in S1 Data.

To determine the cell types that activate executioner caspases in liver, we stained *Sox2-Cre; mCasExpress* livers collected on day 7 after DOX injection with markers of the major cell types in the liver. We found that all ZsGreen^+^ cells in liver expressed the hepatocyte marker HNF4α, and detected no co-localization of ZsGreen with the cholangiocyte marker CK19, the macrophage marker CD68, or the endothelial cell marker CD31 (Fig 2A), suggesting that ECA occurs predominantly, if not exclusively, in hepatocytes. Hepatocytes are highly heterogenous [30]. We analyzed the spatial distribution of ZsGreen^+^ hepatocytes using the method reported by Wei and colleagues [31], in which the position index (P.I.) of a hepatocyte was calculated based on its distance to the closest central vein, which was surrounded by GS (glutamine synthase)^+^ hepatocytes, and the distance to the closest portal vein (Fig 2B). In the homeostatic liver after DOX injection, the percentage of ZsGreen^+^ hepatocytes increased much faster in the pericentral zone 3 (P.I. < 0.33) than in the other two zones (Figs 1C and 2C). At all the time points we analyzed, about 70% of ZsGreen^+^ cells were in zone 3, while less than 10% were in zone 1 (P.I. > 0.66) (Fig 2D). To figure out whether the pericentral enrichment of ZsGreen^+^ hepatocytes are due to uneven distribution of ECA or DOX, we injected DOX to *CAG-rtTA; *tetO-Cre; LSL-tdTomato** mice to induce Cre expression, which leads to tdTomato expression. On day 7 after injection, the tdTomato^+^ cells were uniformly distributed in the liver (S5 Fig). Therefore, the pericentral enrichment of ZsGreen^+^ hepatocytes indicates a higher frequency of ECA in the pericentral region. The cellular and nuclear morphology of ZsGreen^+^ hepatocytes was indistinguishable from that of ZsGreen^−^ hepatocytes (S6A Fig). In addition, few TUNEL^+^ cells were detected in homeostatic livers (S6B Fig), suggesting that ZsGreen^+^ hepatocytes are not apoptotic cells but living cells with ECA.

### Transient ECA is induced in the early stage of liver regeneration

**Fig 3:**
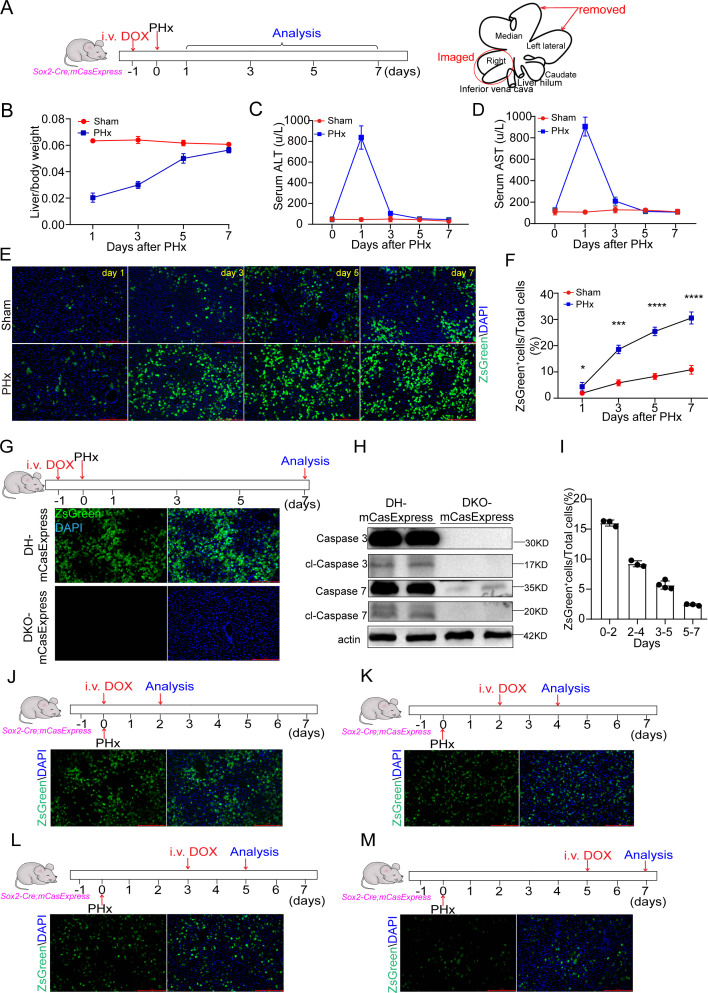
Transient ECA is induced during regeneration after PHx. **(A)** The timeline of the experiments and the schematic to show the lobes removed by surgery and the lobes imaged. **(B–D)** Change of the liver-to-body weight ratio (B), serum ALT (C) and serum AST (D) within 7 days after PHx. Three mice in each group. **(E, F)** The representative images and quantification of ZsGreen^+^ cells in livers at different time points after sham operation or PHx. Scale bar: 100 μm. Five mice per group and 3 fields per mouse. **(G)** The representative images showing the effect of knocking out both *Casp3* and *Casp7* on ZsGreen expression. Scale bar: 100 μm. **(H)** Western blots showing loss of cleaved (cl) and total caspase-3 and caspase-7 in livers with both *Casp3* and *Casp7* knocked out (DKO-mCasExpress) on day 2 after PHx. **(I–M)** Analysis of ZsGreen^+^ cells shown up within a 48-h window during regeneration after PHx. DOX was injected on day 0 (J), day 2 (K), day 3 (L), day 5 (M) and livers were collected 48 h after DOX injection. i.v.: intravenous injection. Four mice in day 3–5 and 3 mice in other groups. Scale bar: 100 μm. Data are presented as the mean ± SD. *: *P* < 0.05. **: *P* < 0.01. ***: *P* < 0.001. ****: *P* < 0.0001. The data underlying the graphs shown in the figure can be found in S1 Data. Raw blot images can be found in S1 Raw Images.

To determine whether executioner caspases are activated during liver regeneration, we performed 70% PHx on *Sox2-Cre; mCasExpress* mice at 24 hours after DOX injection and harvested the livers on day 1, 3, 5, and 7 after PHx (Fig 3A). The liver-to-body weight ratio was mostly restored to the control level by day 7 (Figs 3B and S7A). The levels of serum AST and ALT were restored on day 3 (Fig 3C and 3D). On day 1 after surgery, livers from the PHx group contained more ZsGreen^+^ cells than those from the sham group. The percentage of ZsGreen^+^ cells in the regenerating livers strongly increased from day 1 to day 3 and exhibited dramatic difference compared to the sham group. On day 7, about 30% cells in the livers from the PHx group were ZsGreen^+^, while only 10% cells in the sham-operated livers expressed ZsGreen (Fig 3E and 3F). The elevated ECA was also observed in the regenerated livers from *Alb-Cre; mCasExpress* mice and *CAG-Cre; mCasExpress* mice after PHx (S8 Fig).

To confirm ZsGreen^+^ cells in regenerating livers rely on ECA, we first performed PHx on *Sox2-Cre; mCasExpress*^*mut*^ mice. Few ZsGreen^+^ cells were detected in regenerated *Sox2-Cre; mCasExpress*^*mut*^ livers (S3B Fig). Furthermore, overexpression of caspase inhibitors or genetic deletion of both caspase-3 and caspase-7 suppressed ZsGreen expression after PHx (Figs 3G, 3H, and S9), indicating that the ZsGreen signal was a consequence of ECA.

In Fig 3E and 3F, the 30% ZsGreen^+^ hepatocytes detected on day 7 were the sum of hepatocytes that experienced ECA and their descendants over 7-day regeneration. To monitor the temporal dynamics of ECA in the process of regeneration, we injected DOX at different time points after PHx and collected liver samples at 48 hours post-injection. We found that ZsGreen was expressed in about 16% hepatocytes between day 0 and day 2 post-PHx. The percentage decreased to 9% between day 2 and day 4, and further to 5.5% in day 3–5 window. In the terminating phase of regeneration (between day 5 and day 7), only 2.5% of hepatocytes were ZsGreen^+^ (Fig 3I–3M). TUNEL assays revealed that apoptotic cell death was rare in the first three days after PHx (S7B Fig), suggesting that most of the hepatocytes with ECA were alive. These data indicate that ECA during regeneration is sublethal, transient, and occurs more frequently in the early stage.

To determine whether the elevated fraction of hepatocytes with ECA is specific to the regeneration after PHx, we peritoneally injected 10% carbon tetrachloride (CCl_4_) to *Sox2-Cre; mCasExpress* mice and *CAG-Cre; mCasExpress* mice to induce acute liver injury. Injection of 10% CCl_4_ strongly increased ZsGreen^+^ cells in livers after 7-day regeneration (S10 Fig), suggesting survival from ECA may be a common process involved in liver regeneration.

**Fig 4:**
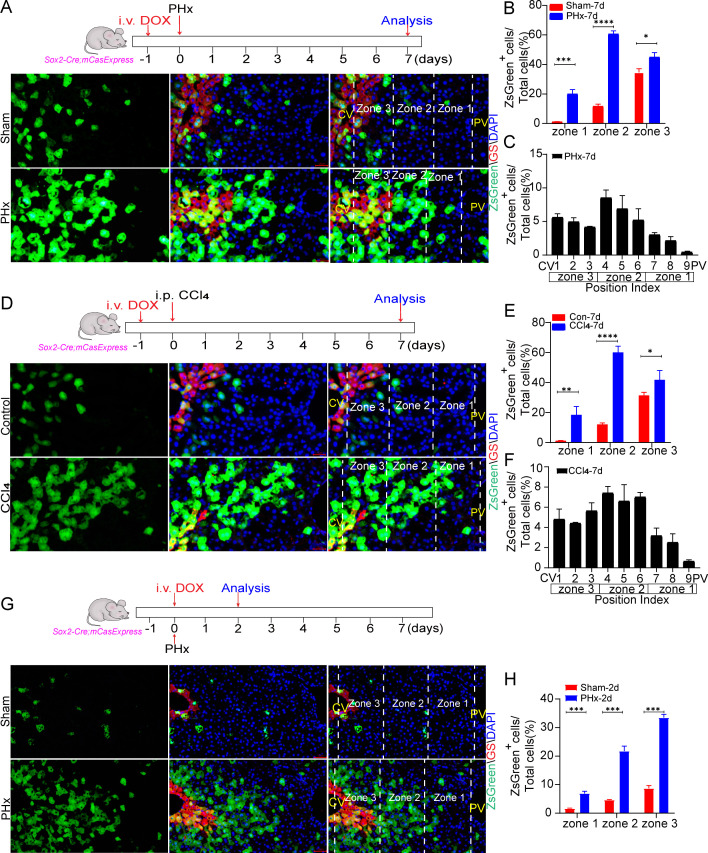
The zonal distribution of hepatocytes with ECA during regeneration. **(A)** The representative images of livers on day 7 after PHx or sham operation with staining of pericentral region marker GS. Scale bar: 25 μm. DOX was injected one day before PHx. **(B)** Quantification of the percentage of ZsGreen^+^ cells in total cells of each zone on the samples shown in (A). **(C)** The percentage of ZsGreen^+^ cells in each position (P.I. = 1–9) of the liver lobule shown in (A). Three mice per group and 3 fields per mouse. **(D)** The representative images of livers on day 7 after injection of CCl_4_ or corn oil (control) with staining of pericentral region marker GS. Scale bar: 25 μm. DOX was injected one day before CCl_4_ injection. i.p. intraperitoneal injection. **(E)** Quantification of the percentage of ZsGreen^+^ cells in total cells of each zone on the samples shown in (D). **(F)** The percentage of ZsGreen^+^ cells in each position (P.I. = 1–9) of the liver lobule shown in (D). Four mice in the CCl_4_ group and 3 mice in the control group. Three fields per mouse. **(G)** The representative images of livers on day 2 after PHx with staining of pericentral region marker GS. Scale bar: 25 μm. DOX was injected on the day of PHx. **(H)** Quantification of the percentage of ZsGreen^+^ cells in total cells of each zone on the samples shown in (G). Three mice per group and 3 fields per mouse. Data are presented as the mean ± SD. *: *P* < 0.05. **: *P* < 0.01. ***: *P* < 0.001. ****: *P* < 0.0001. CV: central vein. PV: portal vein. The data underlying the graphs shown in the figure can be found in S1 Data.

We then analyzed the spatial distribution of ZsGreen^+^ cells in the regenerated livers. On day 7 after PHx or CCl_4_ injection, the highest density of ZsGreen^+^ hepatocytes was observed in zone 2 (Fig 4A, 4B, 4D, and 4E). ZsGreen^+^ cells were also observed in zone 1, but largely excluded from the region adjacent to the portal vein (Fig 4C and 4F). Zone 3 displayed the smallest difference between the regenerated liver and the sham control, possibly due to the accumulation of ZsGreen^+^ cells in the pericentral region over 7 days in the sham group. Thus, we analyzed the distribution of the ZsGreen^+^ cells on day 2 after surgery when the proportion of ZsGreen^+^ cells in the sham group was small. The ZsGreen^+^ cells were strongly increased in all three zones by day 2 after PHx, and the increase in zone 2 and zone 3 was larger than that in zone 1 (Fig 4G and 4H).

During apoptosis, executioner caspases are activated by initiator caspases. To determine whether and which initiator caspases induce ECA in regenerating livers, we reduced the expression of caspase-2, caspase-8 and caspase-9, the initiator caspases in mouse, by in vivo transfection of siRNA targeting *Casp2*, *Casp8*, and *Casp9*, respectively. Silencing any single initiator capase did not alter the percentage of ZsGreen^+^ cells in the liver at day 2 post-PHx (S11A–S11C Fig), nor did it affect liver-to-body weight ratio (S11D Fig), serum ALT/AST levels (S11E Fig) or hepatocyte proliferation (S11F and S11G Fig). These findings suggest that the three initiator caspases may function redundantly to activate executioner caspases, or alternatively, that executioner caspases may be activated via an intiator caspase-independent mechanism during liver regeneration.

### ECA is required for hepatocyte proliferation and liver regeneration

**Fig 5:**
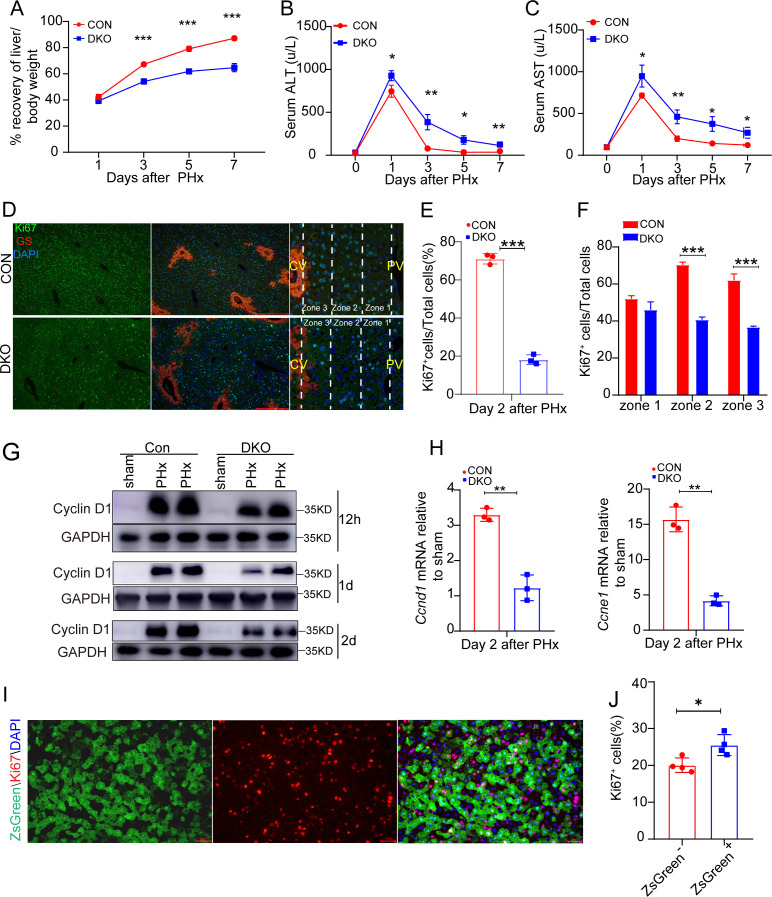
Loss of executioner caspases impairs liver regeneration and hepatocyte proliferation after PHx. **(A–C)** The percentage of recovery of the liver-to-body weight ratio (A), serum ALT (B) and serum AST (C) after PHx in *Casp3*^*flox/flox*^ *Casp7*^*flox/flox*^ (CON) mice and *Alb-Cre*^*+/−*^ *Casp3*^*flox/flox*^ *Casp7*^*flox/flox*^ (DKO) mice. In (A), the average liver-to-body weight ratio of the sham group in each genotype was considered as 100%. Three mice per group. **(D)** The representative images of Ki67 staining in the indicated groups on day 2 after PHx. Scale bar: 100 μm. In the right column are magnified images of the region between a central vein (CV) and a portal vein (PV) to show the distribution of Ki67 across different zones. **(E)** Quantification of the percentage of Ki67^+^ cells in the field at low magnification of the indicated groups on day 2 after PHx. Three mice per group and 3 fields per mice. **(F)** Quantification of the percentage of Ki67^+^ cells in each zone in livers on day 2 after PHx. Three mice per group and 3 fields per mouse. **(G)** Western blots showing Cyclin D1 levels at 12 h, 1 day and 2 days after surgery in the indicated groups. **(H)** The mRNA levels of *Ccnd1* and *Ccne1* in the indicated livers. The average levels in the sham-operated animals were set as 1. Three mice per group. **(I)** The representative images showing co-localization of ZsGreen and Ki67 in the liver on day 3 after PHx. Scale bar: 50 μm. **(J)** Quantification of Ki67^+^ cells in ZsGreen^+^ and ZsGreen^−^ populations on day 3 after PHx. Four mice per group and 3 fields per mouse. Data are presented as the mean ± SD. *: *P* < 0.05. **: *P* < 0.01. ***: *P* < 0.001. The data underlying the graphs shown in the figure can be found in S1 Data. Raw blot images can be found in S1 Raw Images.

To investigate the role of ECA in liver regeneration, we evaluated the effect of inhibition or depletion of executioner caspases. Notably, mice with liver-specific knockout of *Casp3* and *Casp7* or with overexpression of XIAP or p35 exhibited significantly impaired restoration of the liver-to-body weight ratio and elevated serum ALT/AST levels after PHx (Figs 5A–5C and S12A–S12C). Regeneration after PHx relies on hepatocyte proliferation. We found that both inhibition of caspases and genetic deletion of executioner caspases markedly reduced the percentage of Ki67^+^ hepatocytes (Figs 5D–5F and S12D–S12F) and downregulated Cyclin D1 and Cyclin E1 expression in regenerating livers (Figs 5G, 5H, S12G, and S12H). In early phase of regeneration after PHx, hepatocytes with ECA were more abundant in zone 2 and 3 than in zone 1 (Fig 4G and 4H). Accordingly, blocking ECA exerted a more pronounced suppression on hepatocyte proliferation in zone 2 and 3 compared to zone 1 (Figs 5F and S12F). These data suggest that executioner caspases promote hepatocyte proliferation during liver regeneration.

Neither genetic deletion of executioner caspases nor inhibition of caspase activity entirely abolished hepatocyte proliferation (Figs 5D–5H and S12D–S12H), implying the contribution of other signals. This aligns with our observation that both ZsGreen^+^ and ZsGreen^−^ hepatocytes in regenerating *Sox2-Cre; mCasExpress* livers displayed Ki67 staining (Fig 5I). Quantification reveals a higher Ki67^+^ fraction in ZsGreen^+^ hepatocytes than in their ZsGreen^−^ counterparts (Fig 5J), indicating the enhanced proliferative capacity in hepatocytes with ECA. We also noticed that the reduction in Ki67^+^ hepatocytes caused by deletion of executioner caspases exceeded the proportion of ZsGreen^+^ cells in regenerating livers (compare Fig 5E to Fig 3F). This, together with the modest difference in proliferation rates between ZsGreen^+^ and ZsGreen^−^ populations, suggests that hepatocytes with ECA not only proliferate more robustly but may also promote proliferation in neighboring cells.

### Survival of hepatocytes with ECA is necessary for liver regeneration

**Fig 6:**
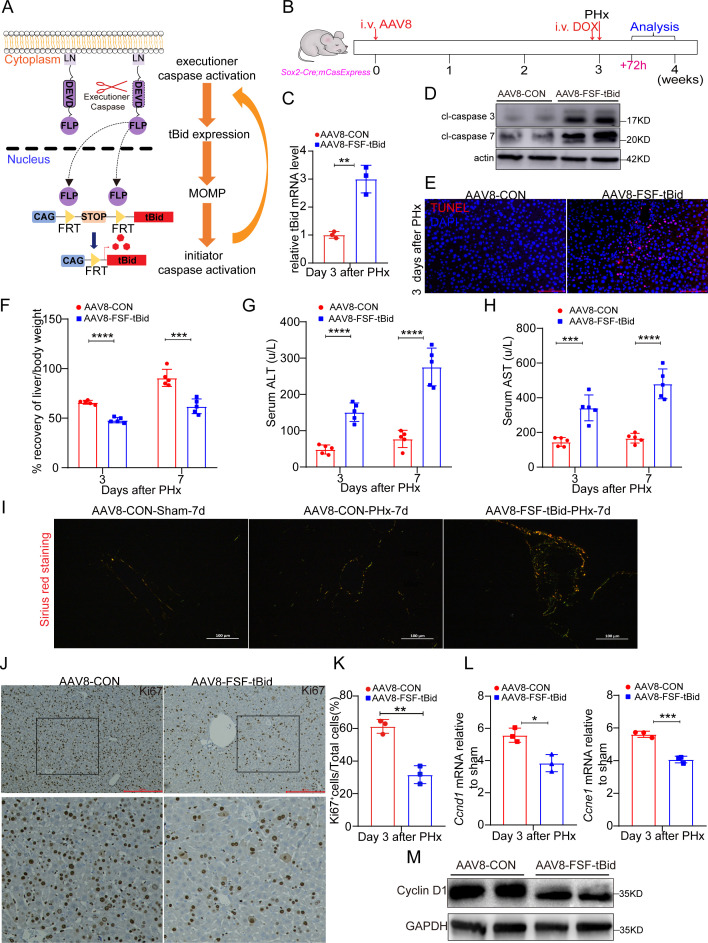
Ablation of hepatocytes with ECA suppresses liver regeneration and hepatocyte proliferation. **(A)** The schematic of ablating cells with ECA. **(B)** The workflow of experiments in this figure. i.v.: intravenous injection. **(C)** The mRNA expression of *tBid* in livers on day 3 after PHx. Three mice per group. **(D)** Western blots showing the levels of cleaved (cl) caspase-3 and cleaved caspase-7 in the indicated livers on day 3 after PHx. **(E)** The representative images of TUNEL assays in livers on day 3 after PHx. Scale bar: 50 μm. **(F)** The percentage of recovery of the liver-to-body weight ratio at the indicated time points after PHx. The average liver-to-body weight ratio of the sham group was considered as 100%. Five mice per group. **(G, H)** The serum ALT (G) and AST (H) at the indicated time points after PHx. Five mice per group. **(I)** Pricosirius red staining of the indicated livers on day 7 after PHx. The images were captured under polarized light. Scale bar: 100 μm. **(J, K)** The representative images and quantification of Ki67 staining in the indicated livers on day 3 after PHx. Scale bar: 100 μm. Three mice per group and 3 fields per mouse. **(L)** The mRNA levels of *Ccnd1* and *Ccne1* in the indicated livers on day 3 after PHx. Three mice per group. **(M)** Western blots showing Cyclin D1 level the indicated livers on day 3 after PHx. Data are presented as the mean ± SD. *: *P* < 0.05. **: *P* < 0.01. ***: *P* < 0.001. ****: *P* < 0.0001. The data underlying the graphs shown in the figure can be found in S1 Data. Raw blot images can be found in S1 Raw Images.

An important function of ECA is to conduct apoptotic cell death. Apoptosis can stimulate compensatory proliferation of the surrounding cells, a process implicated in tissue regeneration, in diverse organisms like *Drosophila*, Hydra, Xenopus [12,13,15]. However, the observation that many ZsGreen^+^ hepatocytes proliferated and apoptotic cells were rarely detected in the early stage of post-PHx regeneration suggest that ECA promotes hepatocyte proliferation independent of apoptosis execution. To further verify this notion, we developed a genetic ablation system consisting of the executioner caspase-activatable FLP (LN-NES-DEVD-FLP) and *CAG-FRT-STOP-FRT-tBid* cassette, which can express tBid after removal of the transcription termination signal (STOP) by FLP. In cells carrying this system, ECA induces overexpression of tBid, the cleaved form of the BH3-only protein Bid that can trigger activation of apoptotic caspase cascade by inducing mitochondrial outer membrane permeabilization (MOMP) [32,33], leading to rapid amplification of ECA and commitment of cell death (Fig 6A). If hepatocytes with ECA eventually die and promote liver regeneration through apoptosis-induced proliferation, ablation of these cells should have no or even positive effect on liver regeneration. Conversely, if survival of these cells is necessary for regeneration, forcing them to die will impair liver regeneration.

We infected *Sox2-Cre; mCasExpress* mice with AAV8 carrying *CAG-FRT-STOP-FRT-tBid* (AAV8-FSF-tBid). Without DOX, *Sox2-Cre; mCasExpress* mice injected with AAV8-FSF-tBid displayed body weight and liver weight similar to those administered with control AAV8 (AAV8-CON) (S13A Fig). Seven days after DOX injection, *Sox2-Cre; mCasExpress* mice injected with AAV8-FSF-tBid exhibited mildly increased serum ALT and AST compared to those with AAV8-CON (S13B Fig), possibly due to ablation of hepatocytes with ECA under homeostasis. We then performed PHx on *Sox2-Cre; mCasExpress* mice administered with AAV8-FSF-tBid or AAV8-CON at 24 hrs after DOX injection (Fig 6B). Three days after PHx, compared to the livers injected with AAV8-CON, those with AAV8-FSF-tBid exhibited higher levels of tBid (Fig 6C), cleaved caspase-3, cleaved caspase-7 (Fig 6D), and TUNEL^+^ cells (Fig 6E) while reduced ZsGreen^+^ cells (S14 Fig), confirming that induction of tBid overexpression in cells with ECA led to more cell death. On day 3 and day 7 after PHx, mice with AAV8-FSF-tBid exhibited a significantly lower liver-to-body weight ratio and markedly higher serum AST and ALT (Fig 6F–6H). Sirius red staining revealed elevated collagen deposition in the liver with AAV8-FSF-tBid after 7-day regeneration (Fig 6I). We then assessed the effect of death of cells with ECA on proliferation in early regeneration. Compared to livers with AAV8-CON, livers with AAV8-FSF-tBid displayed reduced Ki67^+^ hepatocytes (Fig 6J and 6K) and downregulated expression of Cyclin D1 and Cyclin E1 (Fig 6L and 6M), indicative of suppressed proliferation. These data together suggest that survival of cells with ECA is required for rapid hepatocyte proliferation and proper liver regeneration.

### ECA promotes hepatocyte proliferation through enhancing activity of JAK/STAT3 signaling

**Fig 7:**
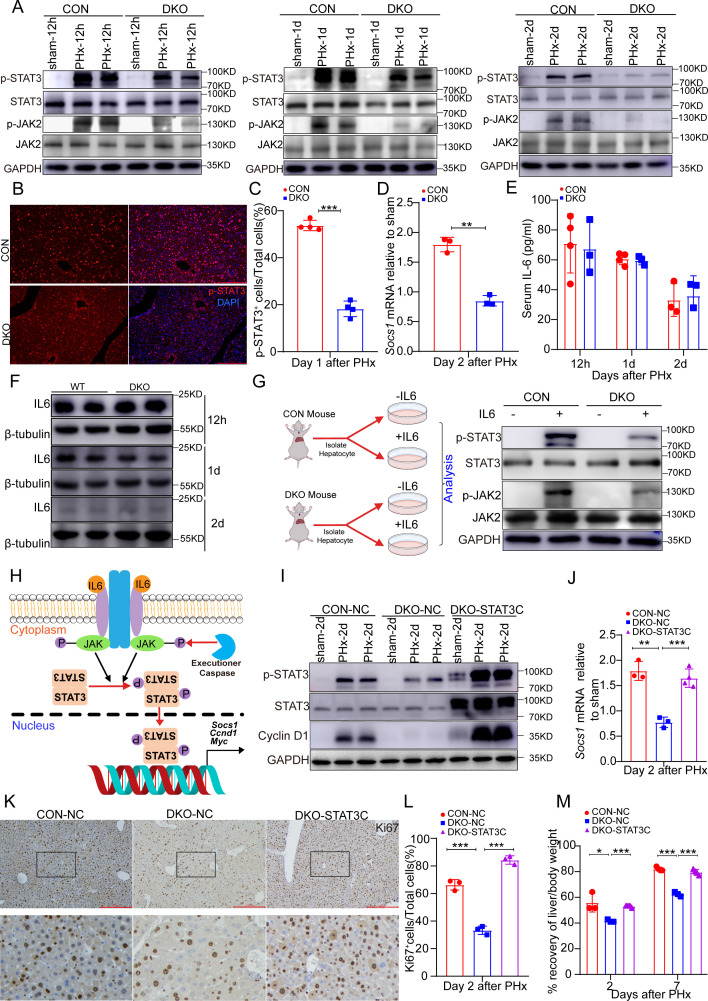
ECA promotes hepatocyte proliferation and liver regeneration by enhancing the activity of JAK/STAT3 pathway. **(A)** Western blots showing the levels of p-STAT3, STAT3, p-JAK2, JAK2 in CON and DKO livers at 12 hrs, day 1 and day 2 after PHx. **(B)** The representative images of pSTAT3 staining on CON and DKO livers on day 1 after PHx. Scale bar: 100 μm. **(C)** Quantification of the percentage of pSTAT3^+^ cells in CON and DKO livers on day 1 after PHx. Four mice per group and 3 fields per mouse. **(D)** The mRNA level of *Socs1* in CON and DKO livers on day 2 after PHx. Three mice per group. **(E)** ELISA results showing IL-6 levels in serum of CON and DKO mice at 12 h, day 1, day 2 after PHx. Four mice per group for CON at 12 h and day 1, and 3 mice per group for others. **(F)** Western blots showing IL-6 levels in CON and DKO livers at 12 h, day 1, day 2 after PHx. **(G)** Western blots showing the levels of p-STAT3, STAT3, p-JAK2, JAK2 in primary hepatocytes isolated from CON or DKO livers with or without 30 min in vitro treatment with IL-6. **(H)** The illustration showing the regulation of JAK/STAT3 pathway by executioner caspases. **(I)** Western blots showing the levels of p-STAT3, STAT3, and Cyclin D1 in livers from CON mice infected with control AAV8 (CON-NC), DKO mice infected with control AAV8 (DKO-NC) and DKO mice infected with AAV8 carrying STAT3C (DKO-STAT3C) on day 2 after PHx. **(J)** The mRNA level of *Socs1* in the indicated groups on day 2 after PHx. Three mice per group for CON-NC and DKO-NC and 4 mice in DKO-STAT3C group. **(K, L)** The representative images and quantification of Ki67 staining in the indicated groups on day 2 after PHx. Scale bar, 100 μm. Three mice per group and 3 fields per mouse. **(M)** The percentage of recovery of the liver-to-body weight ratio of the indicated mice on day 2 and day 7 after PHx. The average liver-to-body weight ratio of the sham group was considered as 100%. Three mice for CON-NC and DKO-NC groups and 4 mice for DKO-STAT3C group. Data are presented as the mean ± SD. *: *P* < 0.05. **: *P* < 0.01. ***: *P* < 0.001. The data underlying the graphs shown in the figure can be found in S1 Data. Raw blot images can be found in S1 Raw Images.

Given the critical role of STAT3 in driving hepatocyte proliferation during liver regeneration [34–36], we investigated whether ECA regulates STAT3 activity. In sham-operated livers, phosphorylated STAT3 (p-STAT3) was almost undetectable, indicating absence of STAT3 activity (Fig 7A). PHx induced robust STAT3 phosphorylation, which was significantly attenuated in livers lacking ECA (Figs 7A–7C and S15A). Consistently, *Socs1*, a target gene of STAT3, was also markedly downregulated in these livers (Figs 7D and S15B), suggesting that executioner caspases enhance STAT3 activation during liver regeneration. Moreover, although livers carrying AAV8-FSF-tBid showed elevated executioner caspase activity (Fig 6D), they exhibited reduced STAT3 activation (S15C and S15D Fig), indicating that strong STAT3 activation in regenerating livers is sustained by ECA in living hepatocytes rather than apoptosis.

During liver regeneration, IL-6 secreted by macrophages and hepatocytes binds to the receptors on hepatocytes to activate JAK family proteins, leading to phosphorylation of STAT3 [28,37–39]. We found reduced JAK2 phosphorylation in livers lacking executioner caspases (Fig 7A), positioning ECA upstream of JAK2. To determine whether ECA enhances JAK/STAT3 activation by modulating ligand production or cellular response to ligands, we first compared IL-6 levels in CON (*Casp3*^*flox/flox*^*; Casp7*^*flox/flox*^) and DKO (*Alb-Cre; Casp3*^*flox/flox*^*; Casp7*^*flox/flox*^) mice. Loss of executioner caspases in hepatocytes had no impact on IL-6 levels in serum and livers (Fig 7E and 7F). Next, we isolated primary hepatocytes from CON and DKO mice and exposed them to IL-6 treatment ex vivo. Little p-JAK2 and p-STAT3 was detected in CON and DKO hepatocytes in the absence of IL-6. Upon IL-6 treatment, DKO hepatocytes showed strongly reduced phosphorylation of JAK2 and STAT3 (Fig 7G), indicating that executioner caspases intrinsically enhance JAK2 and STAT3 activation in the signal-receiving hepatocytes (Fig 7H).

Intriguingly, while loss of executioner caspases dramatically reduced STAT3 activation after PHx, the difference in the intensity of nuclear p-STAT3 in ZsGreen^+^ and ZsGreen^−^ hepatocytes in post-PHx *Sox2-Cre; mCasExpress* livers was small (S15E and S15F Fig), suggesting that ECA not only enhances STAT3 activation cell-autonomously but may also non-autonomously increase STAT3 signaling in the surrounding cells.

To determine whether STAT3 mediates regulation of hepatocyte proliferation by executioner caspases, we overexpressed a constitutively active form of STAT3, STAT3C, in DKO livers and evaluated its effect on regeneration after PHx. Overexpression of STAT3C in DKO livers strongly upregulated the level of phosphorylated STAT3 and the expression of the target gene *Socs1* (Fig 7I and 7J). Overexpression of STAT3C in DKO livers rescued the defects in hepatocyte proliferation (Fig 7K and 7L) and liver regeneration (Fig 7M), indicating that executioner caspases promote hepatocyte proliferation and liver regeneration by increasing STAT3 activity.

## Discussion

In this study, we generated transgenic mice carrying mCasExpress lineage tracing system to track cells that have experienced ECA. Using this system, we demonstrate that while only a small fraction of hepatocytes exhibit ECA in the homeostatic liver, this population expands significantly during liver regeneration. We further show that ECA in the regenerating liver must be controlled at a sublethal level without inducing cell death to promote hepatocyte proliferation. Mechanistically, ECA enhances hepatocyte proliferation through potentiating the activity of JAK/STAT3 signaling.

The mCasExpress sensor reveals that a small group of hepatocytes (enriched in pericentral region) undergoes ECA with no sign of apoptosis in livers under homeostasis. Previous studies from late 1980s detected apoptotic bodies in the pericentral region of the homeostatic liver [40,41]. Recent lineage tracing work has shown that maintenance of liver homeostasis is mainly conducted by hepatocytes derived from the midlobular region [31,42]. These studies suggest that the turnover of hepatocytes may involve apoptosis of the pericentral hepatocytes and generation of new hepatocytes from the midlobular region. Apoptosis can induce non-autonomous ECA and cell death [19,43,44]. In *Drosophila* pupal notum where cell elimination occurs at high frequency, both the eliminated cells and the cells neighboring them activate executioner caspases, but the neighboring cells survive through elevated ERK activity to prevent concomitant extrusion of two or more cells together and ensure the epithelium integrity [19]. Survival of hepatocytes with ECA in the pericentral region may represent a similar important protective mechanism that prevents excessive cell loss during hepatocyte turnover, thereby preserving liver homeostasis.

Using mCasExpress reporter, we observed a striking increase in hepatocytes with ECA during the first 2 days after PHx. The dynamic of ECA is similar to hepatocyte proliferation, which peaks between 36 and 48 h [45,46], suggesting a connection between ECA and hepatocyte proliferation. Overexpression of caspase inhibitors and genetic deletion of both caspase-3 and caspase-7 impaired hepatocyte proliferation and liver regeneration, demonstrating that ECA promotes these processes. The defective post-PHx hepatocyte proliferation and regeneration in livers with *Casp3* or *Casp7* deficiency was previously reported by Li and colleagues as an evidence for apoptosis-induced proliferation [17]. However, both our TUNEL assay results (S7B Fig) and the publications from other labs show that apoptosis rarely occurs in the first three days after PHx [47,48], suggesting that ECA enhances hepatocyte proliferation without inducing apoptosis. Importantly, we found that increasing the activity of executioner caspases to a level that can trigger apoptosis impaired hepatocyte proliferation and liver regeneration, demonstrating that ECA needs to be maintained at a sublethal level to support liver regeneration.

Recent studies have reported a phenomenon named “anastasis”, in which cells activate executioner caspases and exhibit some apoptotic morphological features yet ultimately survive [1,49]. Our group and others have demonstrated that some cancer cells can exploit this process to survive chemotherapy and acquire stronger migratory and angiogenic capacities [50–54]. However, the living hepatocytes with ECA observed in homeostatic and regenerating livers seem different from anastasis as they display no apoptotic morphological features. Interestingly, we found that knocking down any single initiator caspase failed to attenuate ECA during regeneration. This observation suggests either functional redundancy among the initiator caspases, or alternatively, the existence of initiator caspase-independent ECA. Further investigation is required to elucidate the precise mechanisms driving ECA during liver regeneration.

ECA in apoptotic cells can trigger proliferation of surrounding cells by releasing pro-proliferative signals like Wnt3, ATP, PGE2, and EGFR ligands [17–20]. Additionally, non-apoptotic ECA has been reported to intrinsically promote cell proliferation by cleaving some intracellular proteins. For instance, active caspase-3 induces YAP activation through cleavage of α-catenin to promote proliferation of cells in mouse sebaceous glands [5]. Shinoda and colleagues have demonstrated that ECA facilitates proliferation of *Drosophila* wing disc cells by cleaving Acinus [4]. In cancer cells, low level of ECA induces DNA damage and the following activation of NFκB and STAT3, which sustains proliferation and stemness [55]. In this study, we found that loss of executioner caspases significantly attenuated activation of JAK2 and STAT3 following PHx. Overexpression of the constitutively active STAT3 rescued the impaired hepatocyte proliferation and regeneration in livers lacking executioner caspases, suggesting that ECA promotes hepatocyte proliferation and liver regeneration by augmenting JAK/STAT3 pathway. Upon liver injury, JAK/STAT3 signaling in hepatocytes is activated by IL-6 secreted from macrophages and hepatocytes [28,37,39]. We found that deletion of executioner caspases in hepatocytes did not alter serum and hepatic IL-6 levels but impaired JAK/STAT3 activation in response to IL-6 stimulation, suggesting that executioner caspases intrinsically potentiate JAK/STAT3 activation in hepatocytes upon ligand engagement.

Immunostaining of Ki67 and p-STAT3 in regenerating livers carrying mCasExpress revealed that proliferation and STAT3 activation were not restricted to hepatocytes with ECA (ZsGreen^+^). This observation can be attributed to the fact that STAT3 activation and proliferation are also driven by caspase-independent signals, as they were not entirely abolished by loss of executioner caspases. ZsGreen^+^ hepatocytes displayed higher p-STAT3 level and proliferation rate than ZsGreen^−^ hepatocytes in the same liver, supporting the notion that ECA intrinsically enhances the responsiveness of hepatocytes to STAT3-activating ligands in the liver microenvironment. However, compared to the dramatic reduction in STAT3 activation and hepatocyte proliferation caused by depletion of executioner caspase in the whole liver, the differences in STAT3 activation and proliferation between ZsGreen^+^ and ZsGreen^−^ hepatocytes were small. In addition, the decrease in Ki67^+^ and p-STAT3^+^ fractions caused by loss of executioner caspases exceeded the proportion of ZsGreen^+^ hepatocytes detected in regenerating livers. These data imply that in addition to enhancing JAK/STAT3 activation cell-autonomously, sublethal ECA in hepatocytes may also non-cell-autonomously promote STAT3 activation and proliferation in the neighboring cells. This could occur through secretion of ligands other than IL-6 for JAK/STAT3 signaling or other signaling pathways that can converge on STAT3 activation.

While several components in JAK/STAT3 pathway are known caspase substrates, the reported cleavage events typically suppress the pathway activity. For example, STAT3 is cleaved in a caspase-dependent manner in cancer cells treated with staurosporine, resulting in inactivation of STAT3 [56]. In liver, activated caspase-3 induced by CD95L can cleave gp130, a subunit of IL-6 receptor complex, thereby suppressing STAT3 activation [57,58]. Activation of JAK/STAT3 signaling by executioner caspases has, to our knowledge, only been documented in cancer cells. Low-level caspase-3 activation causes DNA damage by activating endonuclease G and caspase-dependent DNase. The DNA damage then leads to phosphorylation of STAT3 via activation of Src [10,55]. However, the mechanism underlying caspase-induced STAT3 activation during liver regeneration may be different from that in cancer cells, as little DNA damage was detected in regenerating livers (S16 Fig). Moreover, our data suggest that executioner caspases regulate STAT3 upstream of JAK, but Src has been reported to activate STAT3 in parallel to JAK [59]. The ECA-induced JAK/STAT3 activation may be mediated by cleavage of negative regulators like SOCS family proteins or tyrosine phosphatases or by increasing the responsiveness of receptors to ligands.

In summary, our work identifies executioner caspases as positive regulators of JAK/STAT3 signaling to promote hepatocyte proliferation during liver regeneration. Rather than triggering apoptosis-induced proliferation, we demonstrate that ECA in hepatocytes must be maintained at a sublethal level to enhance proliferation and regeneration.

## Materials and methods

### Mice

All mouse experiments were performed in accordance with the protocol approved by the Institutional Animal Care and Use Committee at School of Basic Medical Sciences, Shandong University (ECSBMSSDU2023-2-141). Generation of the transgenic mice carrying *CAG-FSF-ZsGreen* and *CAG-LSL-rtTA-TRE-Lyn11-NES-DEVD-FLP* were done by GemPharmatech Co., Ltd (Nanjing, China) using CRISPR-Cas9 technology. *Sox2-Cre* (Stain# 008454), *tetO-Cre* (Strain# 006234) and *CAG-LSL-tdTomato* mice (Strain# 007909) were obtained from the Jackson Laboratory (Bar Harbor, Maine, US). *CAG-Cre* (Strain# T050269), *Alb-Cre* (Strain# T003814), *Casp3*^*flox/flox*^ (Strain# T005781) and *Casp7*^*flox/flox*^ (Strain# T005784) were obtained from GemPharmatech. *CAG-rtTA* mice (Strain# C001185) were obtained from Cyagen Biosciences (Suzhou, China). All the mice were housed in a specific-pathogen-free facility at 22−26 °C and 40%–70% humidity, with a 12/12 h light-dark cycle. To induce expression of *Lyn11-NES-DEVD-FLP*, doxycycline (DOX; Sangon Biotech Cat# A600889-0025) was dissolved in 0.9% NaCl and administered to mice through the tail veins at 5 mg per kg mouse body weight.

### Genotyping

For genotyping, mouse tails (~ 0.1 cm) were incubated with Proteinase K (Spark jade, Cat# AA1906) overnight at 55 °C, followed by 10 min incubation at 100 °C to inactivate Proteinase K. After centrifugation at 12,000 rpm for 10 min, the supernatant containing genomic DNA was applied to PCR. The primers were synthesized by Tsingke Biotech (Beijing, China). The primers used for genotyping are listed in S1 Text.

### Liver injury

Seventy percent PHx was performed as reported in literature [60]. In brief, 8-week-old male mice were anaesthetized with isoflurane and oxygen flow. The abdominal skin and muscle were incised to expose the liver. The left lateral and median hepatic lobes were ligated and removed. After closing the abdominal cavity, betadine was applied to the suture, and the mice were kept in a 37 °C incubator to recover. For CCl_4_-induced liver injury model, 10% CCl_4_ (dissolved in corn oil) were administered intraperitoneally into 8-week-old male mice at a dose of 10 μl per gram body weight. The mice were euthanized at the expected time points, and the liver weight and body weight were measured. The serum AST and ALT were measured by Kingmed Diagnostics (Guangzhou, China). Serum IL-6 was measured using Mouse IL-6 high sensitivity ELISA kit (Multisciences Biotech, Cat# EK206HS).

### AAV injection

AAV8-CAG-XIAP, AAV8-CAG-p35, AAV8-CAG-FSF-tBid, AAV8-CAG-STAT3C, and the control AAV8 virus were provided by GeneChem (Shanghai, China). The virus was injected into 6-week-old mice through tail veins at a dose of 3 × 10^11^ vg per mouse.

### Histology and immunohistochemistry

The liver tissues were fixed with 4% paraformaldehyde (ServiceBio, Cat# G1101) for 48 h, and embedded in paraffin. The embedded tissues were sectioned and stained with hematoxylin and eosin. For immunohistochemistry, the tissue sections were subjected to antigen retrieval with citric acid antigen retrieval buffer (pH6.0) followed by three times wash with phosphate-buffered saline (PBS). Activity of endogenous peroxidase was blocked by incubation with 3% hydrogen peroxide for 30 min. The sections were then incubated with 3% BSA for 30 min at room temperature. Primary antibodies were incubated overnight at 4 °C. Secondary antibodies were incubated at 37 °C for 50 min. The signals were detected using DAB (ORIGENE, Cat# ZLI-9108). The images were taken on an upright microscope (Olympus) and quantified using ImageJ. The antibodies used are listed in S2 Text.

### Immunofluorescent staining

Liver tissues were fixed in 4% paraformaldehyde, equilibrated to 30% sucrose and frozen in O.C.T. compound. 6 μm sections were cut, washed with PBS, and blocked with 5% goat serum. The sections were then incubated with primary antibody at 4 °C overnight and secondary antibody at 37 °C for 1 h. The images were captured on an upright fluorescent microscope (Olympus). All the antibodies used for staining are listed in S2 Text. To detect apoptotic cells, TUNEL assays were performed using TUNEL Apoptosis Detection Kit (Vazyme, Cat# A113-03 and Beyotime C1091) according to the manufacturer’s protocol. For picrosirius red staining, the liver sections were stained according to the published protocol [61] and imaged under polarized light using a NIKON Eclipse ci microscope. Quantification of immunofluorescent staining were done using StrataQuest software (TissueGnostics, Austria).

### Quantification of zonal location

Quantification of zonal location was done according to the literature using StrataQuest software [31]. Glutamine synthase (GS) was stained to mark the central veins. The position index (P.I.) of each ZsGreen^+^ cell was calculated based on its distance to the closest CV (*x*), the distance to the closest PV (*y*), and the distance between the CV and PV (*z*) using the formula P.I. = (*x*^2^ + *z*^2^ − *y*^2^)/(2*z*^2^) (Fig 2B). Cells with P.I. less than 0.33 were considered in zone 3. Cells with P.I. between 0.33 and 0.66 were considered in zone 2. Cells with P.I. more than 0.66 were considered in zone 1.

### Transient knockdown in livers using siRNA

Knocking down *Casp2*, *Casp8*, or *Casp9* in livers was performed following the published protocol with some modification [62]. Briefly, 40 μg siRNA was diluted in 100 μl 5% glucose. 6.4 μl vivo-jetPEI (Polyplus, Cat# 101000040) was also diluted in 100 μl 5% glucose. The diluted vivo-jetPEI was then added to the siRNA solution. The siRNA/vivo-jetPEI mixture was mixed by vortexing and incubated at room temperature for 30 min. Then the mixture was injected through the caudal vein. To get a better knockdown, injection of siRNA/vivo-jetPEI mixture was done two days before PHx and repeated once 12 hrs before PHx. Liver samples were collected at day 2 post-PHx. The sequences for siRNA were listed in S1 Text.

### Isolation of primary hepatocytes and treatment with IL-6

Hepatocytes were isolated by two-step collagenase perfusion modified from a published protocol [63]. The liver was perfused through the portal vein with the perfusion buffer (HBSS containing 0.5 mM EGTA, pH7.4) followed by the digestion buffer (HBSS containing 5 mM CaCl_2_, 0.1 mg/ml Collagenase IV, and 10 mM HEPES, pH 7.4). The cell suspension was centrifuged at 50*g* for 3 min. The pellets were resuspended in DMEM/F12 (Gibco, Cat# 8123375) supplemented with 10% FBS and 100 U/ml penicillin and 100 μg/ml streptomycin and seeded on collagen-coated 6-well plates at a density of 3.5 × 10^5^ cells/well. After 16-h culture, the hepatocytes were serum-starved for 16 h and then treated with human recombinant IL-6 (Peprotech, Cat# 200-06) for 30 min.

### RNA extraction and quantitative RT-PCR

Total RNA was extracted using FastPure cell/tissue total RNA isolation kit V2 (Vazyme, Cat# R112-01) and converted to cDNA using HiScript III RT SuperMix for qPCR kits (Vazyme, Cat# R323-01). Quantitative PCR was performed using ChamQ SYBR Color qPCR Master Mix (Vazyme, Cat# Q411-02). The primers used are listed in S1 Text.

### Western blot

The frozen liver tissues were homogenized and lysed in RIPA buffer (Sigma, Cat# SLBZ0792). Protein concentrations were measured with the BCA assay kit (Vazyme, Cat# E112-02). 20 µg proteins were applied to SDS-PAGE and then transferred to PVDF membranes. The membranes were incubated with primary antibody (1:1,000) overnight at 4 °C and then with the secondary antibody (1:2,000) at room temperature for 1 hr. Proteins were detected using Chemiluminescent Substrates (Spark jade, Cat# ED0015-C) and Tanon 5200 Multi Chemiluminescence imager (Tanon Science & Technology Co., Shanghai, China). The antibodies used are listed in S2 Text.

### Statistical analysis

Data are presented as the mean ± standard deviation (SD). Statistical significance was determined using *t* test for two-sample comparison or one-way ANOVA for comparing three or more samples. The Tukey test was used to derive an adjusted *P*-value for multiple comparisons. *P* < 0.05 was considered as statistically significant. The assumption of equal variance was validated by *F*-test. Statistical analyses were performed using GraphPad Prism version 8 (GraphPad Software). The sample sizes were chosen empirically based on the observed effects and previous reports. The sample size for each experiment is listed in the figure legends. When collecting and analyzing data of immunohistochemistry and immunofluorescent staining, the investigators were blinded to the group allocation.

### Ethics statement

All mouse experiments were performed in accordance with the protocol approved by the Institutional Animal Care and Use Committee at School of Basic Medical Sciences, Shandong University (ECSBMSSDU2023-2-141).

## Supporting information

S1 Fig *mCasExpress* and *Sox2-Cre; mCasExpress* mice have body and liver morphology comparable to wild type mice. **(A)** Pictures of the 8-week-old mice with the indicated genotypes. **(B–E)** The body weight (B), liver-to-body weight ratio (C), serum AST (D) and serum ALT (E) of the *wild type*, *mCasExpress* and *Sox2-Cre; mCasExpress* mice. Three mice per group. Data are presented as the mean ± SD. ns: no significance. **(F)** The representative images of H & E staining of livers from the indicated mice. Scale bar: 100 μm. The data underlying the graphs shown in the figure can be found in S1 Data.
(TIF)

S2 Fig Expression of ZsGreen requires the presence of DOX, Cre and FLP. **(A)** The relative mRNA level of *LN-DEVD-FLP* before (−DOX) and after DOX injection. Three mice in −DOX group and 5 mice in all the other groups. Data are presented as the mean ± SD. **(B)** The representative images of the livers from *Sox2-Cre; mCasExpress* mice without (w/o) DOX injection. Scale bar: 100 μm. **(C)** The representative images of the livers from *mCasExpress* mice and *Sox2-Cre; FSF-ZsGreen* mice on day 7 after DOX injection. Scale bar: 100 μm. i.v.: intravenous injection. The data underlying the graphs shown in the figure can be found in S1 Data.
(TIF)

S3 Fig ZsGreen expression requires cleavage of DEVD site. **(A)** The schematic showing mutation of the executioner caspase-specific cleavage site. **(B)** The representative images of livers from *Sox2-Cre; mCasExpress*^*mut*^ mice on day 7 after sham operation or PHx. Scale bar: 100 μm.
(TIF)

S4 Fig Inhibition of executioner caspase activity or expression does not affect liver weight or function. **(A)** RT-qPCR results confirm the overexpression of XIAP or p35. Three mice in AAV8-CON group and 4 mice in AAV8-p35 or AAV8-XIAP group. **(B, C)** The liver-to-body weight ratio (4 mice per group), serum AST and ALT (5 mice per group) in mice injected with the indicated AAV8. **(D)** The image of CON (*Casp3*^*flox/flox*^*; Casp7*^*flox/flox*^) and DKO (*Alb-Cre^+/-^; Casp3*^*flox/flox*^*; Casp7*^*flox/flox*^) mice and Western blots showing loss of caspase-3 and caspase-7 in DKO livers. **(E, F)** The liver-to-body weight ratio (E), serum ALT and AST levels (F) of the 7-week-old male CON and DKO mice. Three or 4 mice per group. Data are presented as the mean ± SD. **: *P* < 0.01. ***: *P* < 0.001. ns: no significance. The data underlying the graphs shown in the figure can be found in S1 Data. Raw blot images can be found in S1 Raw Images.
(TIF)

S5 Fig Response to DOX is uniform across all zones in the liver lobule. The upper part shows the mating strategy and the lower are the representative images of tdTomato expression in livers from *CAG-rtTA; tetO-Cre; LSL-tdTomato* mice on day 7 after DOX injection. Scale bar: 200 μm.
(TIF)

S6 Fig ZsGreen^+^ hepatocytes in homeostatic livers are not apoptotic. **(A)** Immunostaining of *Sox2-Cre; mCasExpress* livers on day 7 after DOX injection with Phalloidin, which labels F-actin, and DAPI to show the morphology of the cells and the nuclei. Arrows point to examples of ZsGreen^+^ cells. Scale bar, 50 μm. **(B)** TUNEL staining of homeostatic livers on day 1, 3, 5, 7, and 14 after DOX injection. Livers collected two days after CCl_4_ injection is used as positive control. Scale bar, 15 μm.
(TIF)

S7 Fig Liver regeneration after PHx. **(A)** Representative images of the appearance and histology of the liver after sham operation or at different time points after PHx. Scale bar, 100 μm. **(B)** TUNEL staining of livers at different time points during regeneration after PHx. Livers on day 2 after CCl_4_ injection were used as the positive control. Scale bar, 100 μm. In the lower row are the magnified images showing the morphology of TUNEL^+^ cells, if any.
(TIF)

S8 Fig ECA is elevated during regeneration after PHx. The representative images and quantification of ZsGreen^+^ cells in *Alb-Cre; mCasExpress* livers **(A)** and *CAG-Cre; mCasExpress* livers **(B)** on day 7 after PHx. Scale bar: 100 μm. Four mice in the PHx group of *Alb-Cre; mCasExpress* and 3 mice per group for all the others. Three fields per mouse. Data are presented as the mean ± SD. ***: *P* < 0.001. ****: *P* < 0.0001. The data underlying the graphs shown in the figure can be found in S1 Data.
(TIF)

S9 Fig Overexpression of caspase inhibitors strongly reduces expression of ZsGreen. The representative images **(A)** and quantification **(B)** of ZsGreen^+^ cells showing the effect of XIAP or p35 overexpression on ZsGreen expression. Scale bar: 100 μm. Four mice per group and 3 fields per mouse. The data underlying the graphs shown in the figure can be found in S1 Data.
(TIF)

S10 Fig ECA is dramatically increased in livers during regeneration after acute CCl_4_ injury. **(A)** The representative images of the appearance and histology of livers after injection of corn oil (CON) or CCl_4_. Scale bar, 100 μm. **(B, C)** The representative images and quantification of ZsGreen^+^ cells in livers from *Sox2-Cre; mCasExpress* mice (B) or *CAG-Cre; mCasExpress* mice (C) on day 7 after injection of CCl_4_ or corn oil (CON). Scale bar: 100 μm. Three mice per group and 3 fields per mouse. i.v.: intravenous injection. i.p. intraperitoneal injection. Data are presented as the mean ± SD. ***: *P* < 0.001. ****: *P* < 0.0001. The data underlying the graphs shown in the figure can be found in S1 Data.
(TIF)

S11 Fig Knocking down *Casp2*, *Casp8* or *Casp9* did not affect ECA in regenerating livers. **(A)** Western blots showing the knockdown efficiency and specificity of si*Casp2*, si*Casp8*, and si*Casp9* in livers. **(B)** The representative images of livers transfected with si*Casp2*, si*Casp8*, or si*Casp9* on day 2 after PHx. Scale bar: 100 μm. **(C–E)** Quantification of the percentage of ZsGreen^+^ cells (C), liver-to-body weight ratio (D) and serum ALT and AST (E) in the indicated groups. Four mice in each group. **(F,G)** The representative images and quantification of Ki67 staining in the indicated groups. Scale bar: 100 μm. Four mice in each group and 3 fields per mouse. The data underlying the graphs shown in the figure can be found in S1 Data. Raw blot images can be found in S1 Raw Images.
(TIF)

S12 Fig Inhibition of caspase activity suppresses liver regeneration and hepatocyte proliferation. **(A–C)** The percentage of recovery of the liver-to-body weight ratio (A), serum ALT (B), and serum AST (C) at the indicated time points after PHx. In (A), the average liver/body weight ratio of the sham group in each genotype was considered as 100%. *N* = 3 for all groups except the AAV8-CON-PHx on day 2, for which *N* = 4. **(D)** The representative images of Ki67 staining in the indicated groups on day 2 after PHx. Scale bar: 100 μm. In the right column are magnified images of the region between a central vein (CV) and a portal vein (PV) to show the distribution of Ki67 in different zones. Staining of livers from sham group is used as a negative control. **(E)** Quantification of the percentage of Ki67^+^ cells in the field at low magnification of the indicated groups on day 2 after PHx. Three mice per group and 3 fields per mice. **(F)** Quantification of the percentage of Ki67^+^ cells in each zone in livers on day 2 after PHx. Three mice per group and 3 fields per mice. **(G)** Western blots showing the protein level of Cyclin D1 in the indicated groups on day 2 after PHx. **(H)** The mRNA levels of *Ccnd1* and *Ccne1* in the indicated groups on day 2 after PHx. The level in the sham-operated animals was set as 1. Three mice per group. Data are presented as the mean ± SD. *: *P* < 0.05. **: *P* < 0.01. ***: *P* < 0.001. ns: no significance. The data underlying the graphs shown in the figure can be found in S1 Data. Raw blot images can be found in S1 Raw Images.
(TIF)

S13 Fig Ablating cells with ECA in homeostatic livers mildly affects liver function. **(A)** Without (w/o) DOX injection, AAV8-FSF-tBid showed little effect on liver morphology, body weight and liver-to-body weight ratio. Four mice per group. 3W: 3 weeks after AAV8 injection. **(B)** Mice administered with AAV8-FSF-tBid exhibited similar liver-to-body weight ratio but mildly increased serum AST and ALT on day 7 after DOX injection. Four mice per group. i.v.: intravenous injection. Data are presented as the mean ± SD. *: *P* < 0.05. **: *P* < 0.01. ns: no significance. The data underlying the graphs shown in the figure can be found in S1 Data.
(TIF)

S14 Fig Overexpression of tBid promotes more cells with ECA to die. The representative images of livers with AAV8-CON or AAV8-FSF-tBid on day 3 after PHx. Livers with AAV8-FSF-tBid showed dramatically reduced ZsGreen^+^ cells, and the remaining ZsGreen^+^ cells showed abnormal morphology. Scale bar, 100 μm. In the right column are magnified images of the red rectangular regions.
(TIF)

S15 Fig Inhibition of caspase activity suppresses JAK/STAT3 activation after PHx. **(A)** Western blots showing the protein levels of p-STAT3 and STAT3 in livers with the indicated AAV8 on day 1 and 2 after PHx. **(B)** The mRNA level of *Socs1* in the indicated groups on day 1 and 2 after PHx. The level in the sham-operated animals was set as 1. Three mice per group. **(C, D)** The protein levels of p-STAT3 and STAT3 (C) and the mRNA level of *Socs1* (D) in livers with the indicated AAV8 on day 3 after PHx. Three mice per group. **(E)** The representative images of p-STAT3 staining in *Sox2-Cre; mCasExpress* livers on day 2 after PHx. Scale bar: 25 μm. **(F)** Quantification of the integrated intensity of nuclear p-STAT3 in ZsGreen^+^ cells and ZsGreen^−^ cells in *Sox2-Cre; mCasExpress* livers. Five mice were included and about 8 fields were quantified per mouse. Each point in the graph represents the average intensity of p-STAT3 in cells in one field. Data are presented as the mean ± SD. *: *P* < 0.05. **: *P* < 0.01. ***: *P* < 0.001. The data underlying the graphs shown in the figure can be found in S1 Data. Raw blot images can be found in S1 Raw Images.
(TIF)

S16 Fig PHx does not induce DNA damage in livers. γH2AX staining in CON or DKO mice on day 1 and 2 after PHx. Scale bar: 100 μm.
(TIF)

S1 Text The list of oligonucleotides used in this study. (DOCX)

S2 Text The list of antibodies used in this study. (DOCX)

S1 Data Numerical values underlying all graphs in the main body and supporting information. (XLSX)

S1 Raw Images Uncropped version of all Western Blot images in the main body and supporting information. (PDF)

## Abbreviations

AAV8: adeno-associated virus serotype 8
PHx: partial hepatectomy
ALT: alanine transaminase
AST: aspartate transaminase
CCl_4_: carbon tetrachloride
CV: central vein
DOX: doxycycline
ECA: executioner caspase activation
EGFR: epidermal growth factor receptor
GS: glutamine synthase
MOMP: mitochondrial outer membrane permeabilization
NES: nuclear export signal
PBS: phosphate-buffered saline
PGE2: prostaglandin E2
P.I.: position index
PV: portal vein
