# Supplementary figures and images for: Sublethal executioner caspase activation in hepatocytes promotes liver regeneration through the JAK/STAT3 pathway
Source: PLoS Biol. 2025 Aug 28;23(8):e3003357. doi: 10.1371/journal.pbio.3003357 (PMC12407553; doi:10.1371/journal.pbio.3003357)

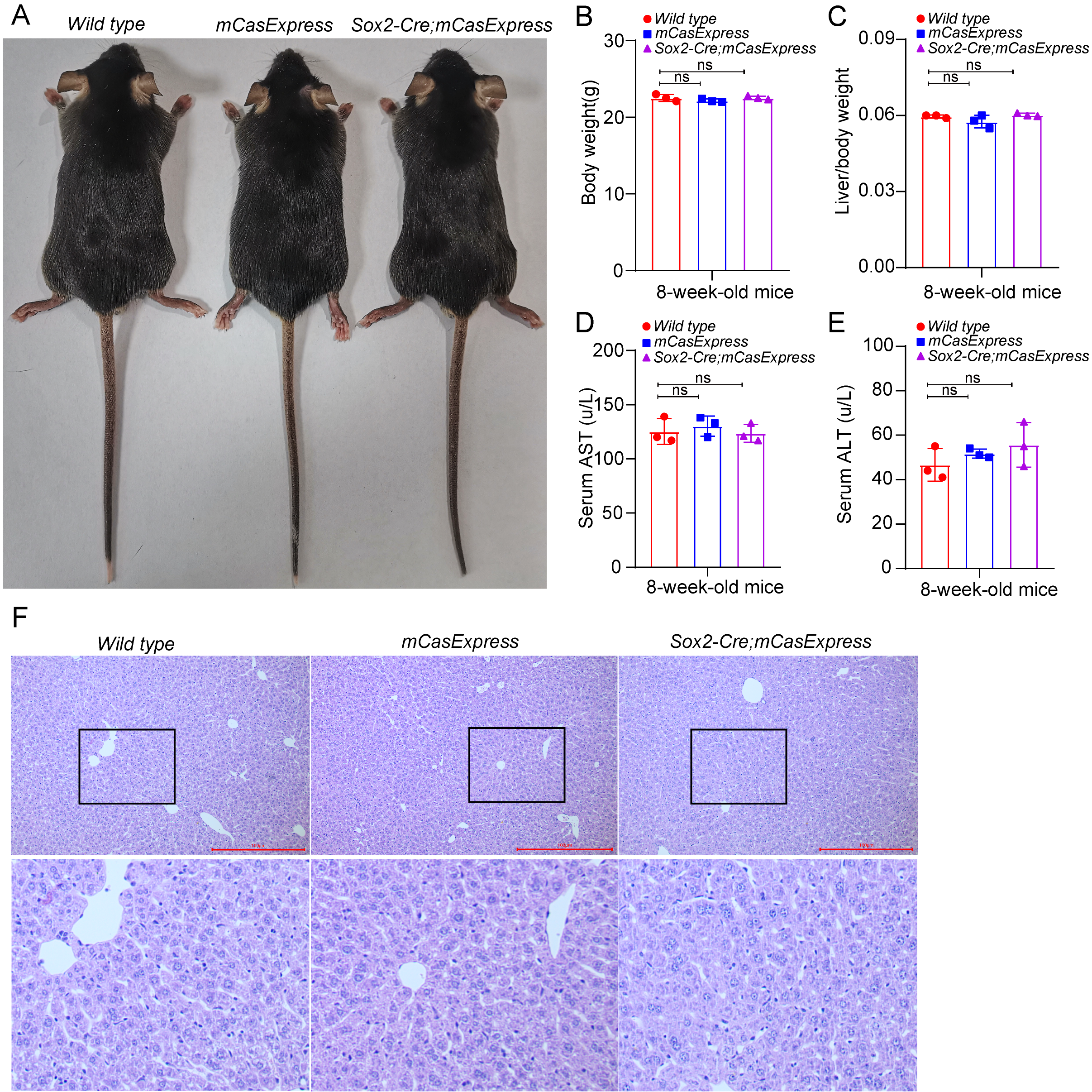

Supplement: S1 Fig — (A) Pictures of the 8-week-old mice with the indicated genotypes. (B–E) The body weight (B), liver-to-body weight ratio (C), serum AST (D) and serum ALT (E) of the wild type, mCasExpress and Sox2-Cre; mCasExpress mice. Three mice per group. Data are presented as the mean ± SD. ns: no significance. (F) The representative images of H & E staining of livers from the indicated mice. Scale bar: 100 μm. The data underlying the graphs shown in the figure can be found in S1 Data. (TIF) [file pbio.3003357.s001.tif]

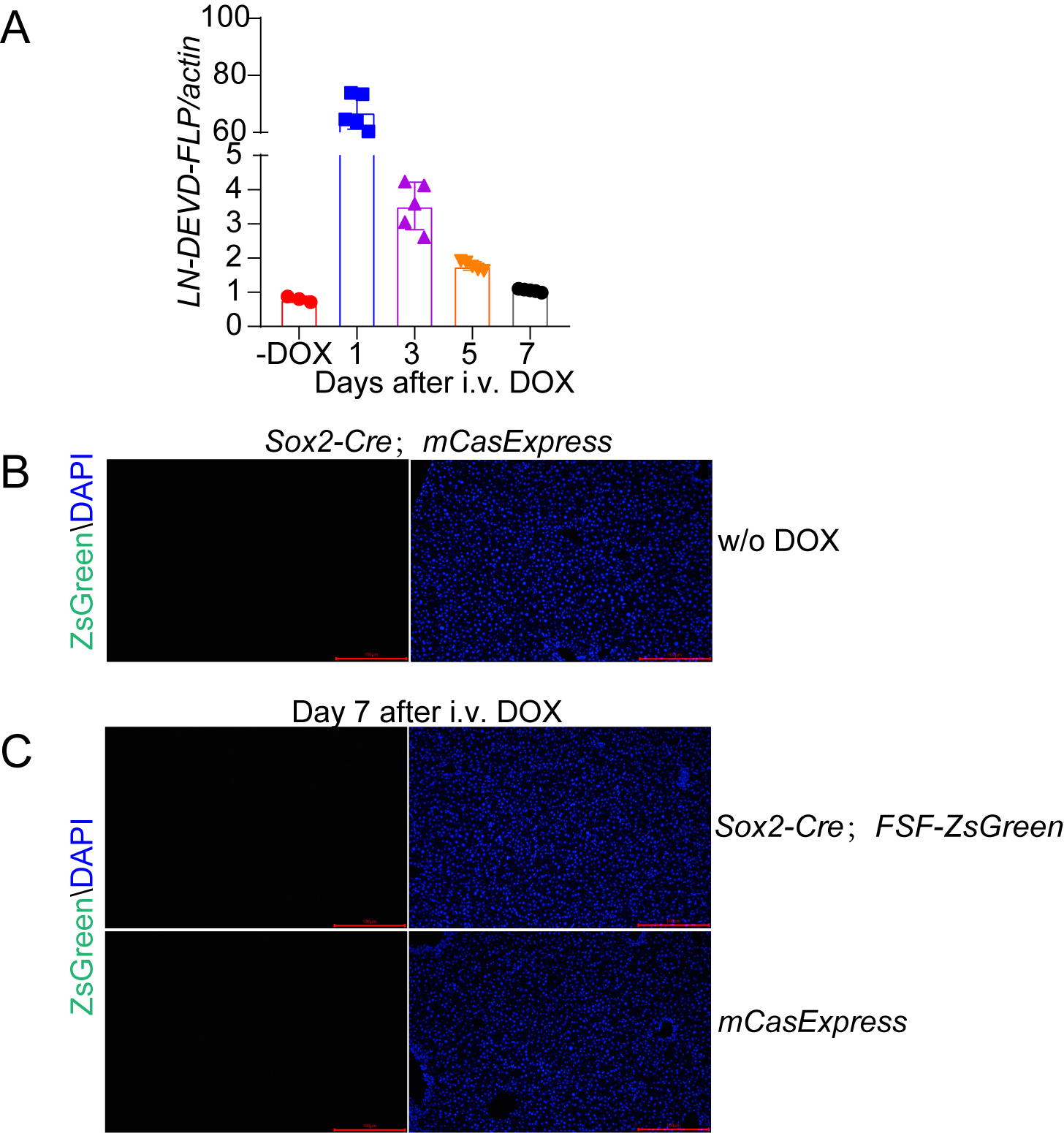

Supplement: S2 Fig — (A) The relative mRNA level of LN-DEVD-FLP before (−DOX) and after DOX injection. Three mice in −DOX group and 5 mice in all the other groups. Data are presented as the mean ± SD. (B) The representative images of the livers from Sox2-Cre; mCasExpress mice without (w/o) DOX injection. Scale bar: 100 μm. (C) The representative images of the livers from mCasExpress mice and Sox2-Cre; FSF-ZsGreen mice on day 7 after DOX injection. Scale bar: 100 μm. i.v.: intravenous injection. The data underlying the graphs shown in the figure can be found in S1 Data. (TIF) [file pbio.3003357.s002.tif]

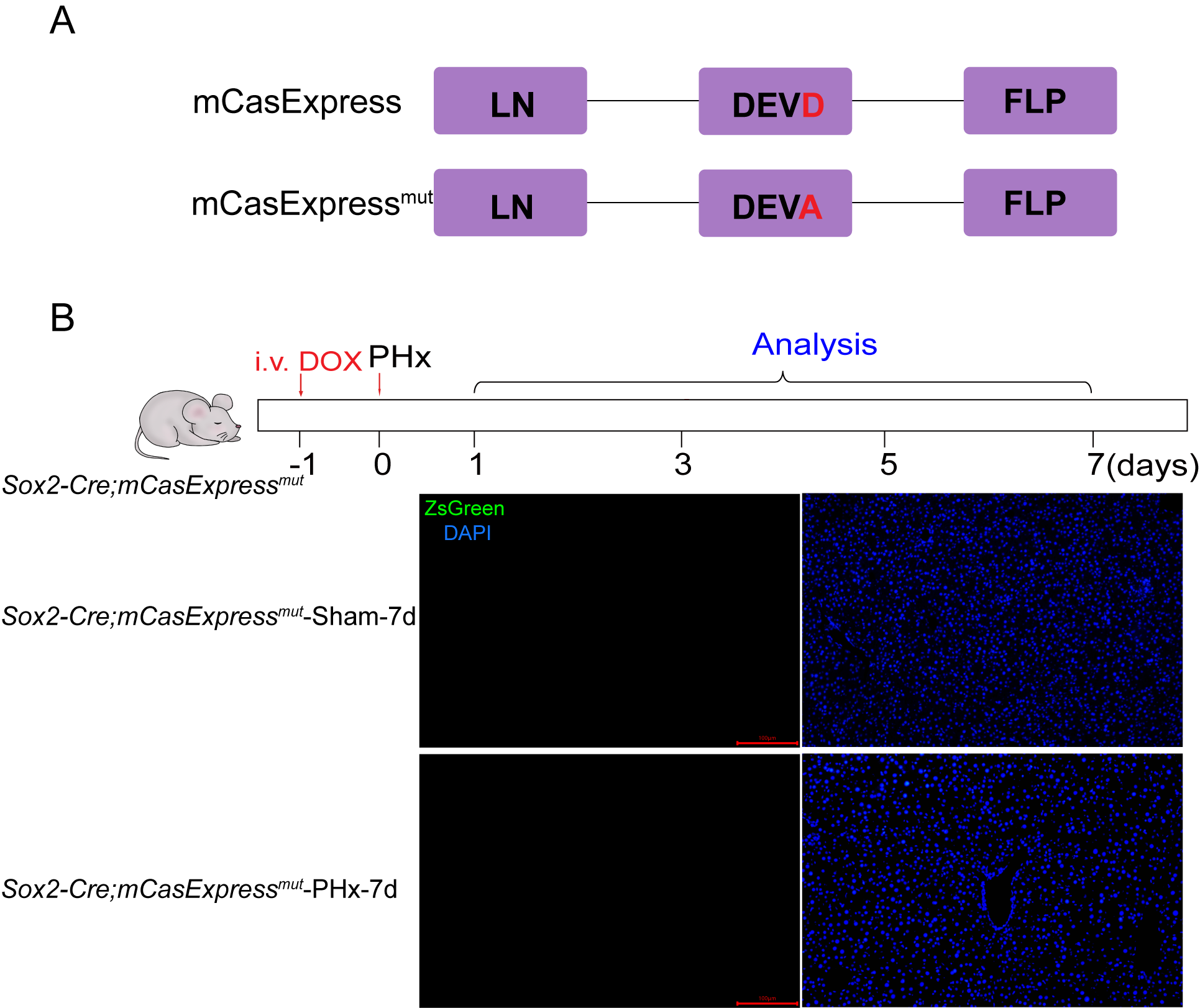

Supplement: S3 Fig — (A) The schematic showing mutation of the executioner caspase-specific cleavage site. (B) The representative images of livers from Sox2-Cre; mCasExpressmut mice on day 7 after sham operation or PHx. Scale bar: 100 μm. (TIF) [file pbio.3003357.s003.tif]

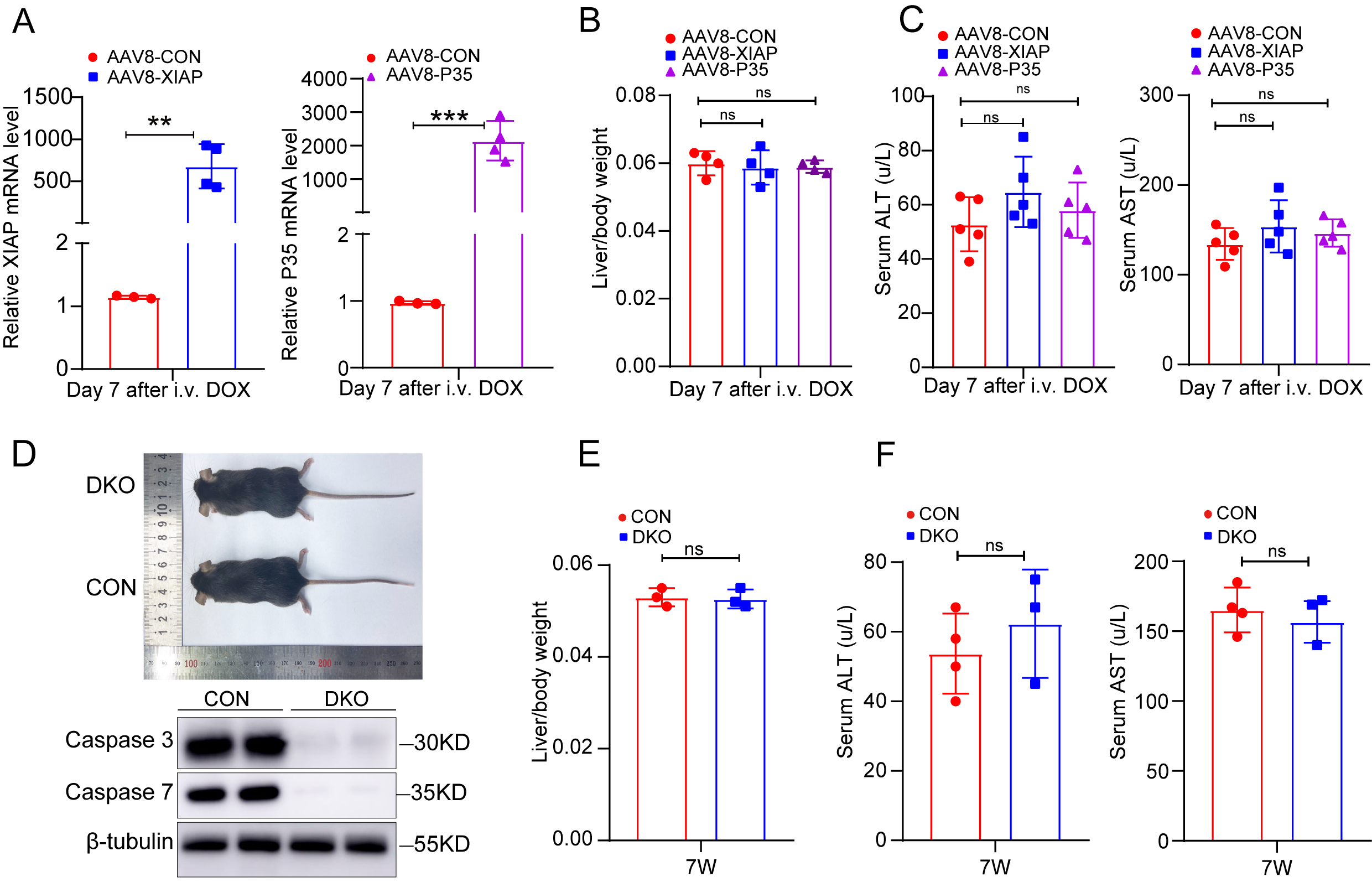

Supplement: S4 Fig — (A) RT-qPCR results confirm the overexpression of XIAP or p35. Three mice in AAV8-CON group and 4 mice in AAV8-p35 or AAV8-XIAP group. (B, C) The liver-to-body weight ratio (4 mice per group), serum AST and ALT (5 mice per group) in mice injected with the indicated AAV8. (D) The image of CON (Casp3flox/flox; Casp7flox/flox) and DKO (Alb-Cre+/-; Casp3flox/flox; Casp7flox/flox) mice and Western blots showing loss of caspase-3 and caspase-7 in DKO livers. (E, F) The liver-to-body weight ratio (E), serum ALT and AST levels (F) of the 7-week-old male CON and DKO mice. Three or 4 mice per group. Data are presented as the mean ± SD. **: P < 0.01. ***: P < 0.001. ns: no significance. The data underlying the graphs shown in the figure can be found in S1 Data. Raw blot images can be found in S1 Raw Images. (TIF) [file pbio.3003357.s004.tif]

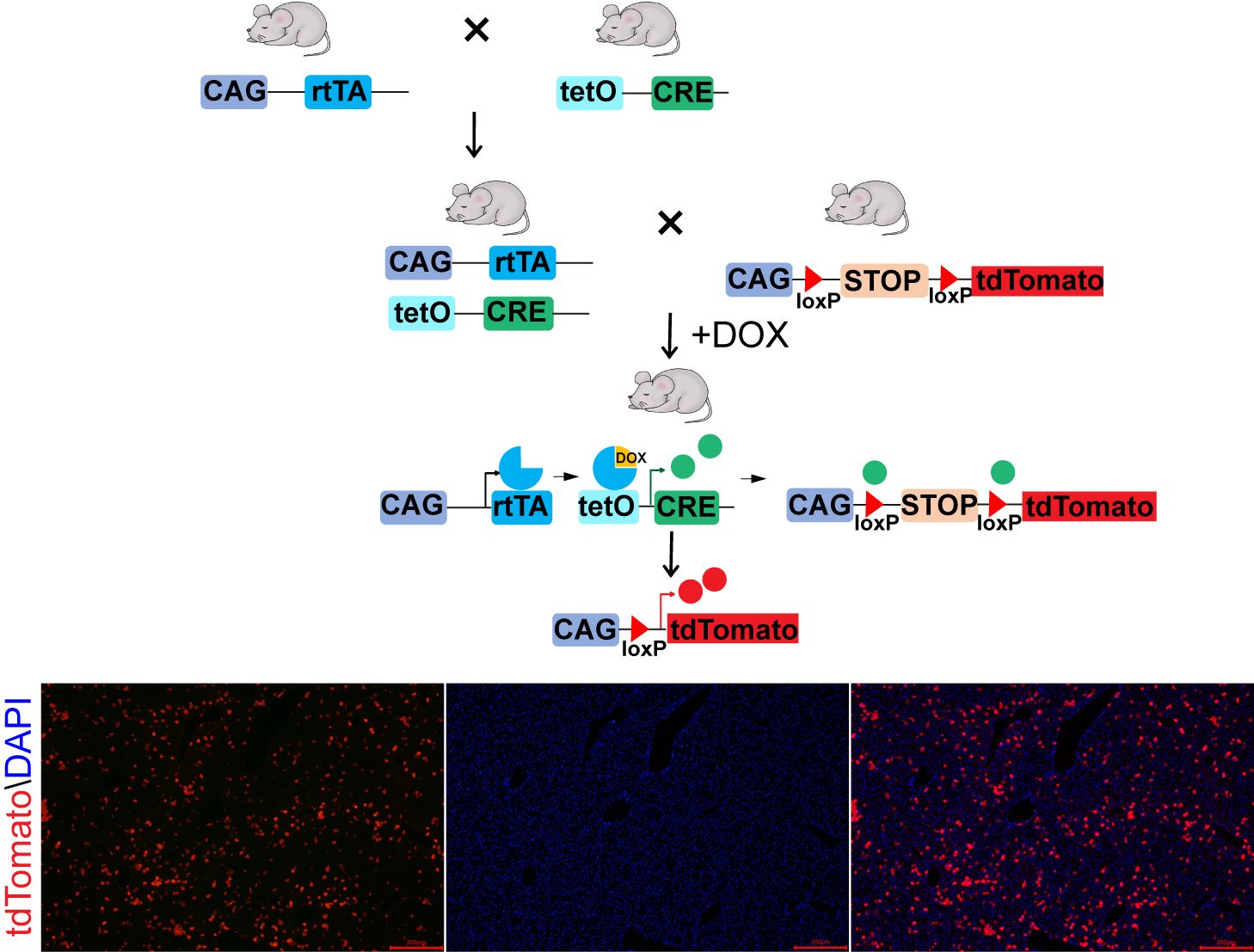

Supplement: S5 Fig — The upper part shows the mating strategy and the lower are the representative images of tdTomato expression in livers from CAG-rtTA; tetO-Cre; LSL-tdTomato mice on day 7 after DOX injection. Scale bar: 200 μm. (TIF) [file pbio.3003357.s005.tif]

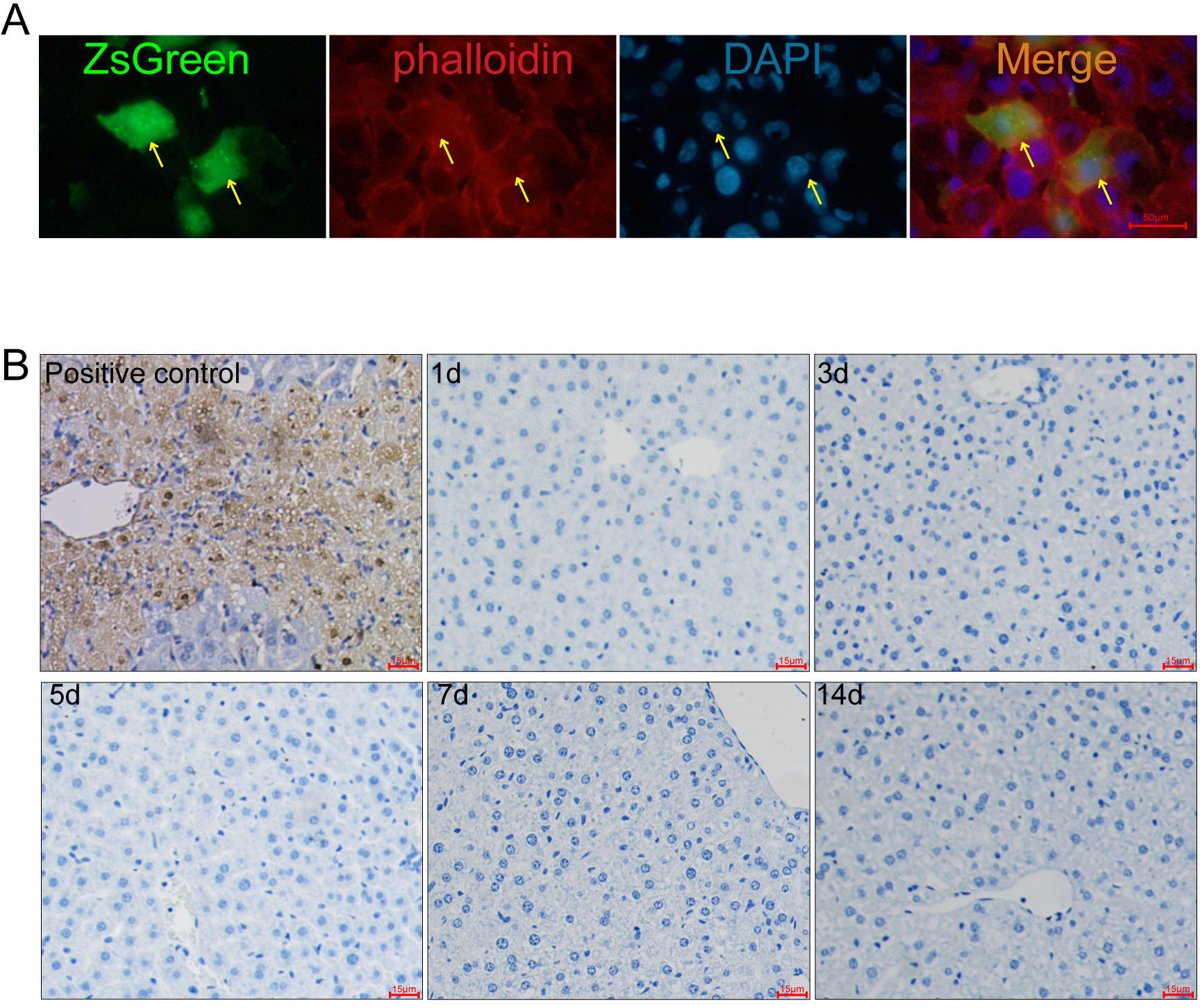

Supplement: S6 Fig — (A) Immunostaining of Sox2-Cre; mCasExpress livers on day 7 after DOX injection with Phalloidin, which labels F-actin, and DAPI to show the morphology of the cells and the nuclei. Arrows point to examples of ZsGreen+ cells. Scale bar, 50 μm. (B) TUNEL staining of homeostatic livers on day 1, 3, 5, 7, and 14 after DOX injection. Livers collected two days after CCl4 injection is used as positive control. Scale bar, 15 μm. (TIF) [file pbio.3003357.s006.tif]

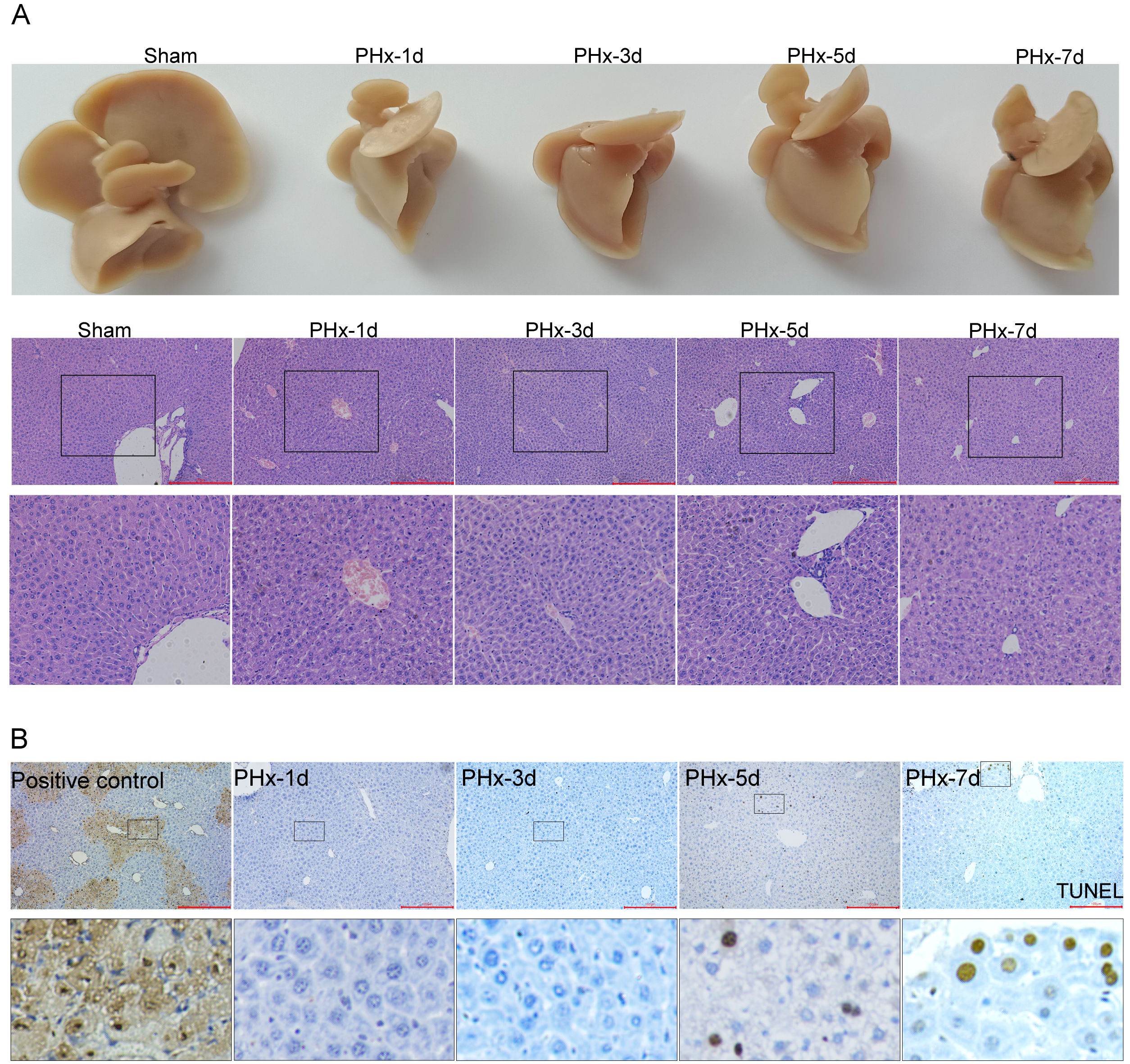

Supplement: S7 Fig — (A) Representative images of the appearance and histology of the liver after sham operation or at different time points after PHx. Scale bar, 100 μm. (B) TUNEL staining of livers at different time points during regeneration after PHx. Livers on day 2 after CCl4 injection were used as the positive control. Scale bar, 100 μm. In the lower row are the magnified images showing the morphology of TUNEL+ cells, if any. (TIF) [file pbio.3003357.s007.tif]

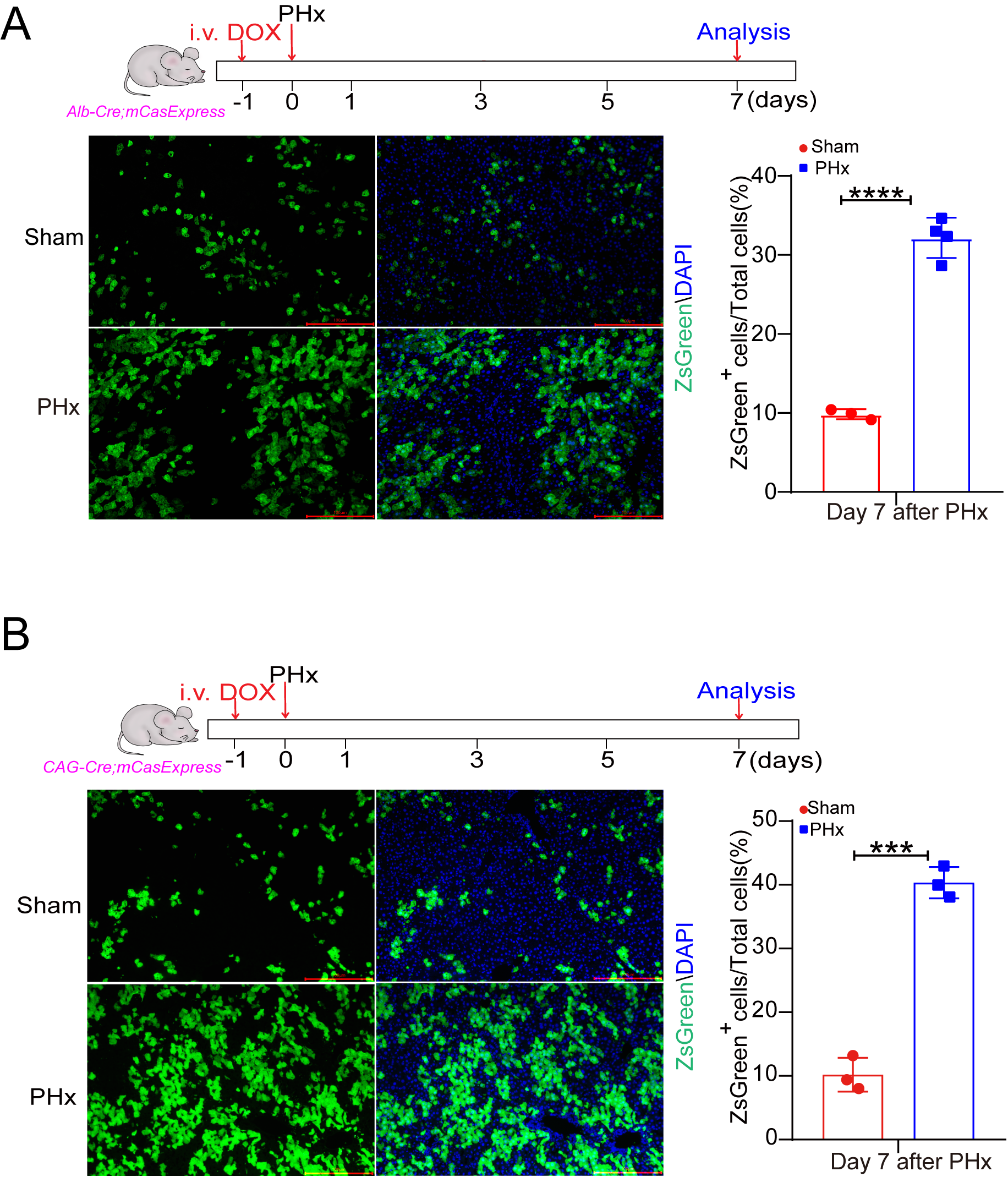

Supplement: S8 Fig — The representative images and quantification of ZsGreen+ cells in Alb-Cre; mCasExpress livers (A) and CAG-Cre; mCasExpress livers (B) on day 7 after PHx. Scale bar: 100 μm. Four mice in the PHx group of Alb-Cre; mCasExpress and 3 mice per group for all the others. Three fields per mouse. Data are presented as the mean ± SD. ***: P < 0.001. ****: P < 0.0001. The data underlying the graphs shown in the figure can be found in S1 Data. (TIF) [file pbio.3003357.s008.tif]

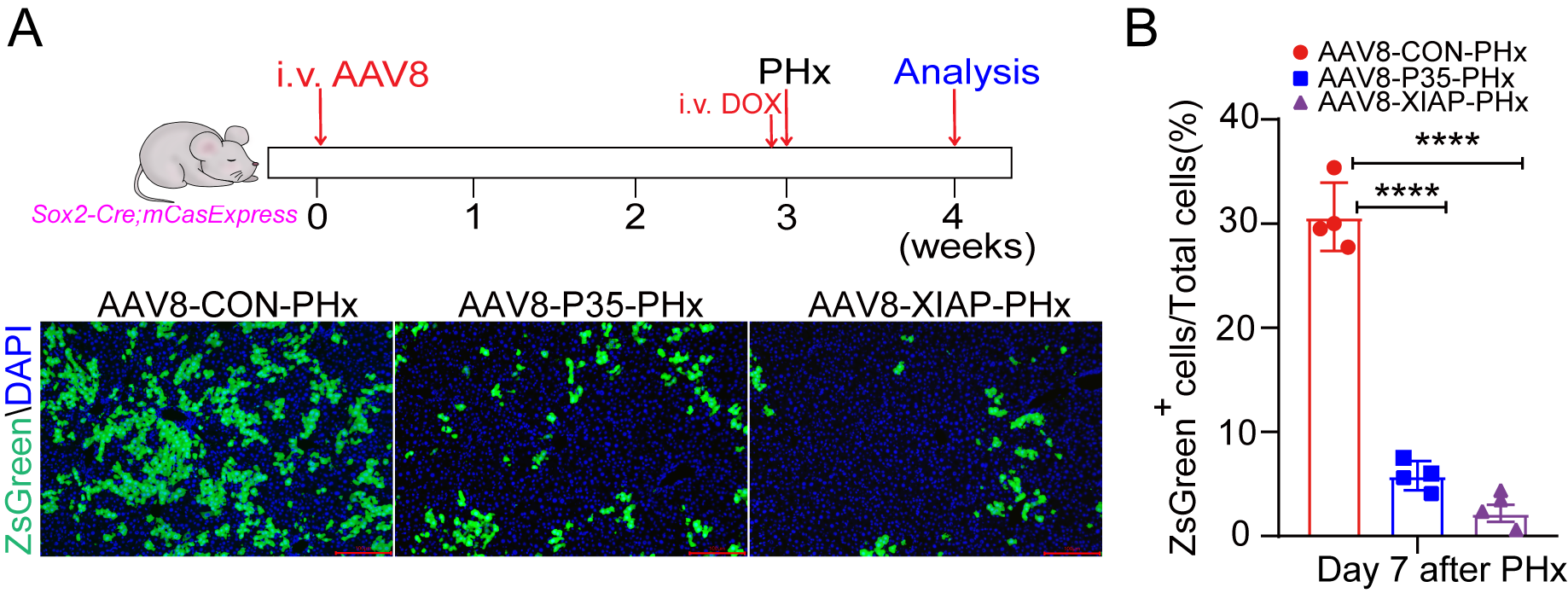

Supplement: S9 Fig — The representative images (A) and quantification (B) of ZsGreen+ cells showing the effect of XIAP or p35 overexpression on ZsGreen expression. Scale bar: 100 μm. Four mice per group and 3 fields per mouse. The data underlying the graphs shown in the figure can be found in S1 Data. (TIF) [file pbio.3003357.s009.tif]

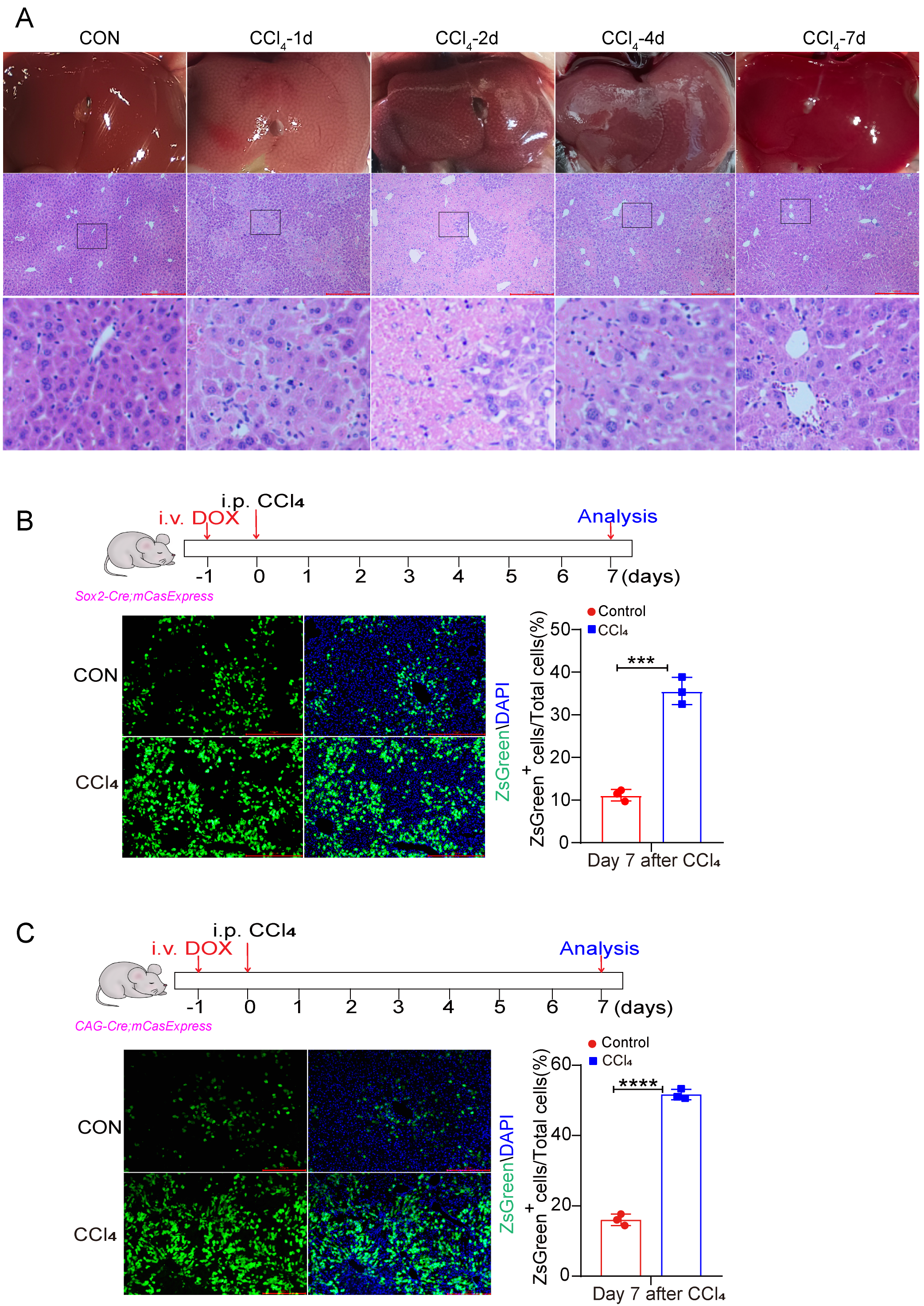

Supplement: S10 Fig — (A) The representative images of the appearance and histology of livers after injection of corn oil (CON) or CCl4. Scale bar, 100 μm. (B, C) The representative images and quantification of ZsGreen+ cells in livers from Sox2-Cre; mCasExpress mice (B) or CAG-Cre; mCasExpress mice (C) on day 7 after injection of CCl4 or corn oil (CON). Scale bar: 100 μm. Three mice per group and 3 fields per mouse. i.v.: intravenous injection. i.p. intraperitoneal injection. Data are presented as the mean ± SD. ***: P < 0.001. ****: P < 0.0001. The data underlying the graphs shown in the figure can be found in S1 Data. (TIF) [file pbio.3003357.s010.tif]

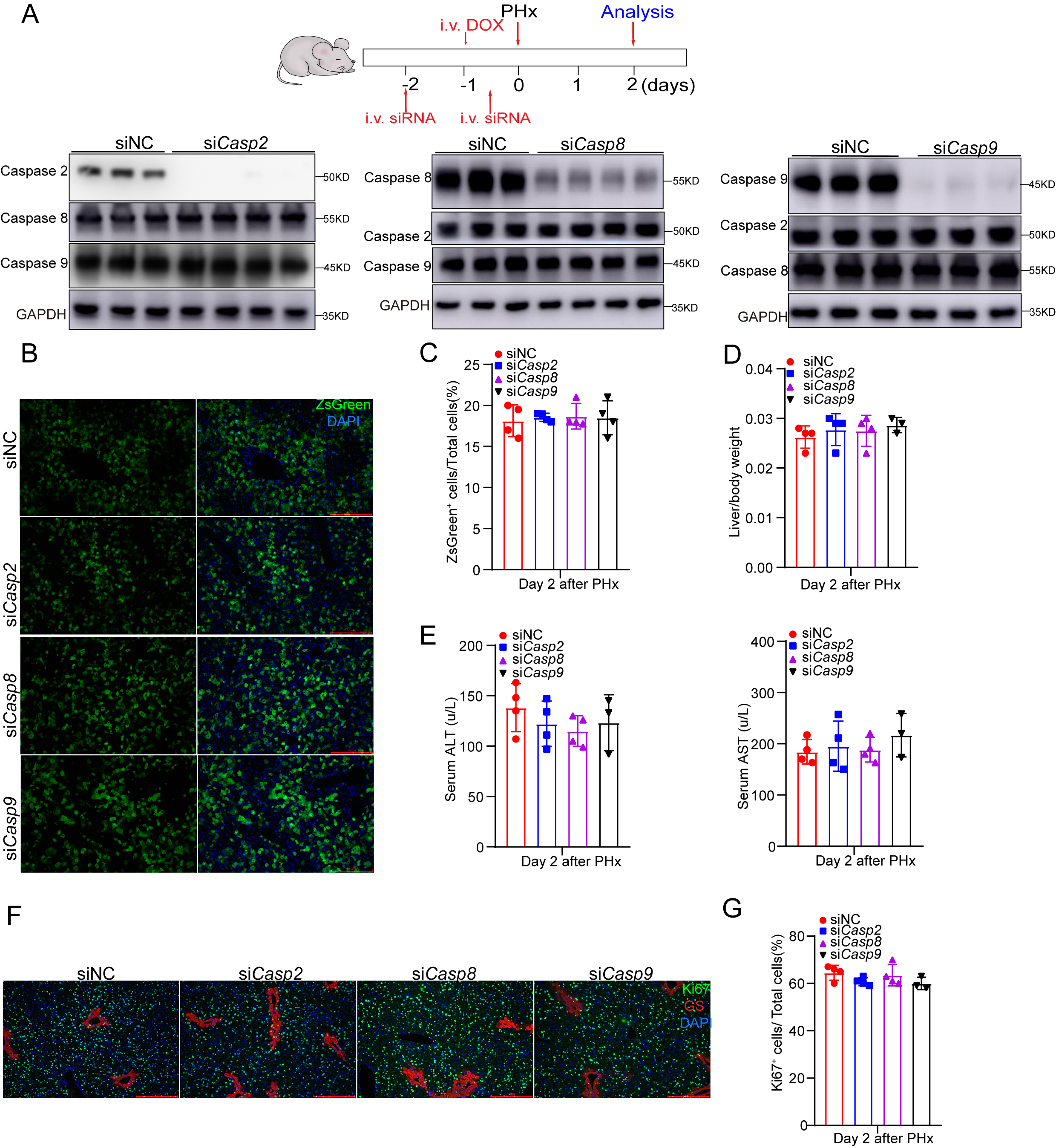

Supplement: S11 Fig — (A) Western blots showing the knockdown efficiency and specificity of siCasp2, siCasp8, and siCasp9 in livers. (B) The representative images of livers transfected with siCasp2, siCasp8, or siCasp9 on day 2 after PHx. Scale bar: 100 μm. (C–E) Quantification of the percentage of ZsGreen+ cells (C), liver-to-body weight ratio (D) and serum ALT and AST (E) in the indicated groups. Four mice in each group. (F,G) The representative images and quantification of Ki67 staining in the indicated groups. Scale bar: 100 μm. Four mice in each group and 3 fields per mouse. The data underlying the graphs shown in the figure can be found in S1 Data. Raw blot images can be found in S1 Raw Images. (TIF) [file pbio.3003357.s011.tif]

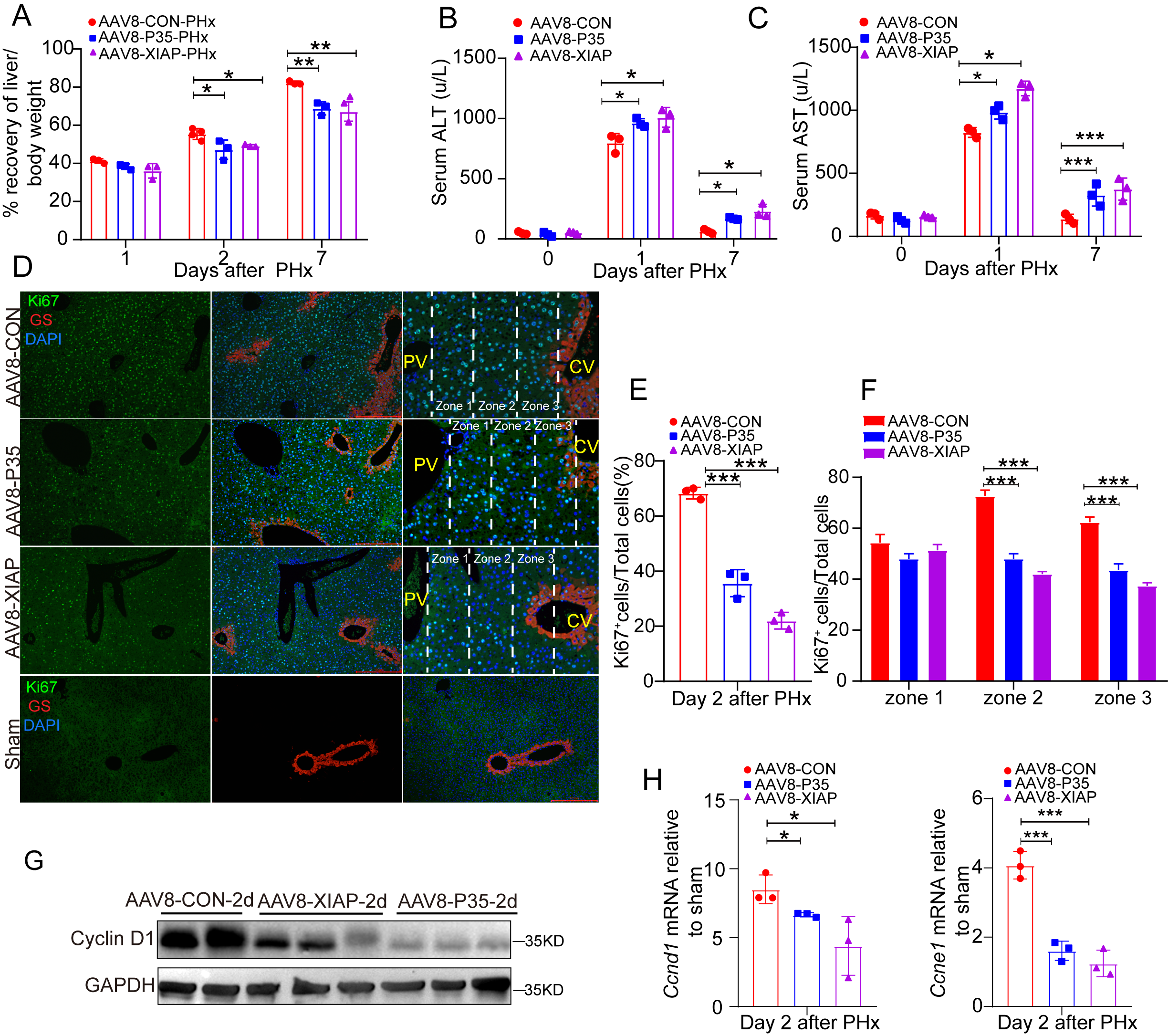

Supplement: S12 Fig — (A–C) The percentage of recovery of the liver-to-body weight ratio (A), serum ALT (B), and serum AST (C) at the indicated time points after PHx. In (A), the average liver/body weight ratio of the sham group in each genotype was considered as 100%. N = 3 for all groups except the AAV8-CON-PHx on day 2, for which N = 4. (D) The representative images of Ki67 staining in the indicated groups on day 2 after PHx. Scale bar: 100 μm. In the right column are magnified images of the region between a central vein (CV) and a portal vein (PV) to show the distribution of Ki67 in different zones. Staining of livers from sham group is used as a negative control. (E) Quantification of the percentage of Ki67+ cells in the field at low magnification of the indicated groups on day 2 after PHx. Three mice per group and 3 fields per mice. (F) Quantification of the percentage of Ki67+ cells in each zone in livers on day 2 after PHx. Three mice per group and 3 fields per mice. (G) Western blots showing the protein level of Cyclin D1 in the indicated groups on day 2 after PHx. (H) The mRNA levels of Ccnd1 and Ccne1 in the indicated groups on day 2 after PHx. The level in the sham-operated animals was set as 1. Three mice per group. Data are presented as the mean ± SD. *: P < 0.05. **: P < 0.01. ***: P < 0.001. ns: no significance. The data underlying the graphs shown in the figure can be found in S1 Data. Raw blot images can be found in S1 Raw Images. (TIF) [file pbio.3003357.s012.tif]

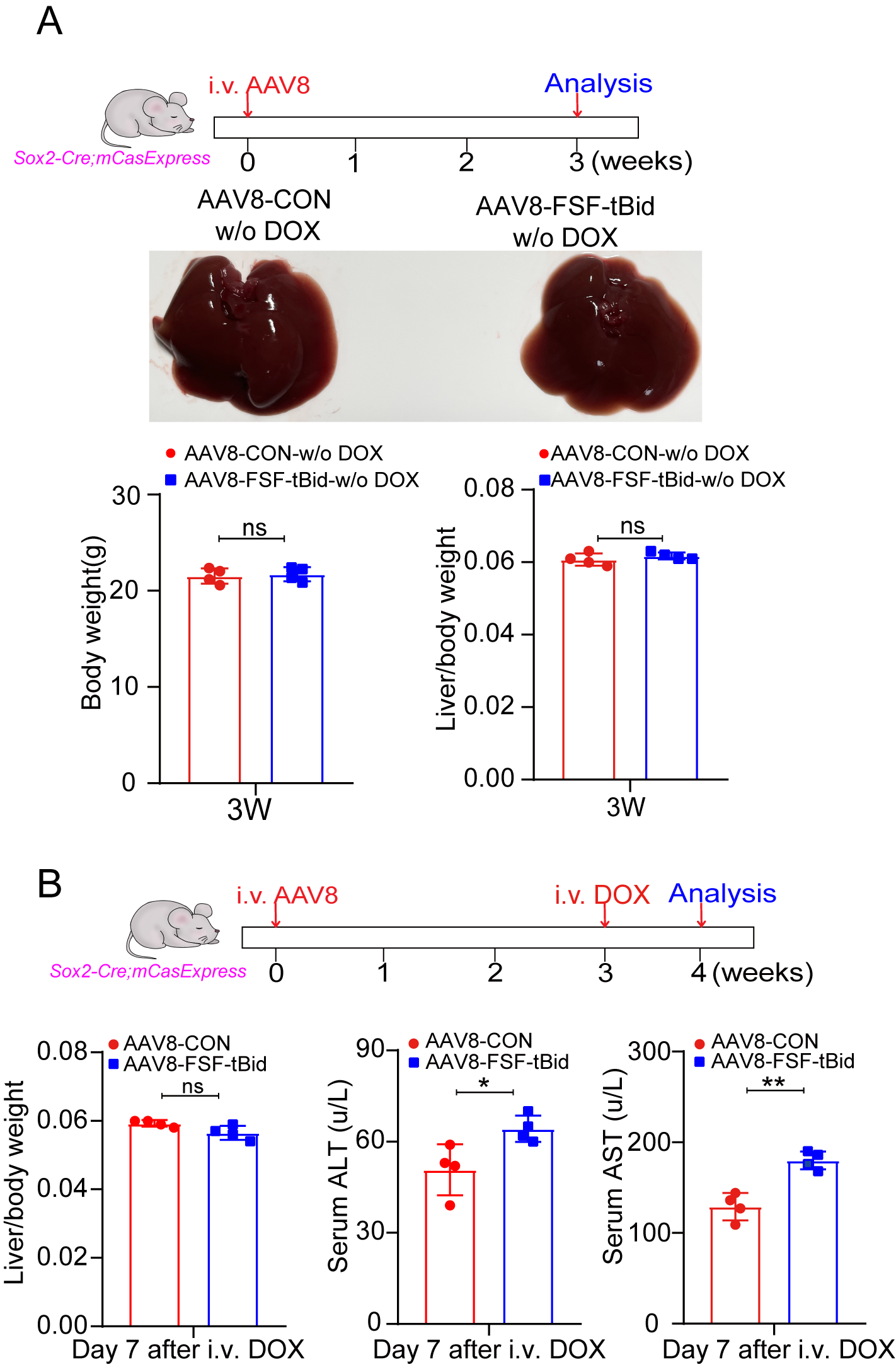

Supplement: S13 Fig — (A) Without (w/o) DOX injection, AAV8-FSF-tBid showed little effect on liver morphology, body weight and liver-to-body weight ratio. Four mice per group. 3W: 3 weeks after AAV8 injection. (B) Mice administered with AAV8-FSF-tBid exhibited similar liver-to-body weight ratio but mildly increased serum AST and ALT on day 7 after DOX injection. Four mice per group. i.v.: intravenous injection. Data are presented as the mean ± SD. *: P < 0.05. **: P < 0.01. ns: no significance. The data underlying the graphs shown in the figure can be found in S1 Data. (TIF) [file pbio.3003357.s013.tif]

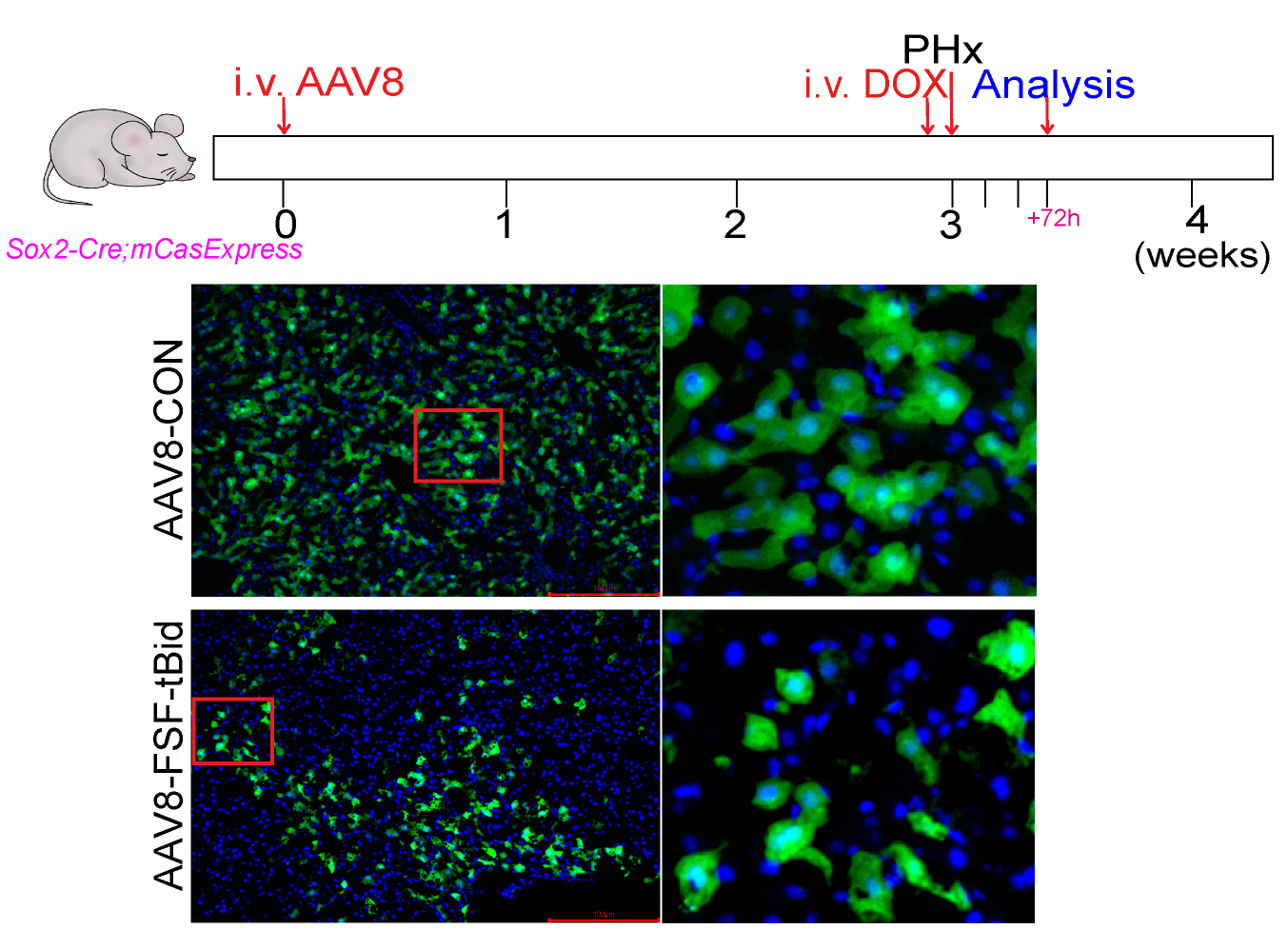

Supplement: S14 Fig — The representative images of livers with AAV8-CON or AAV8-FSF-tBid on day 3 after PHx. Livers with AAV8-FSF-tBid showed dramatically reduced ZsGreen+ cells, and the remaining ZsGreen+ cells showed abnormal morphology. Scale bar, 100 μm. In the right column are magnified images of the red rectangular regions. (TIF) [file pbio.3003357.s014.tif]

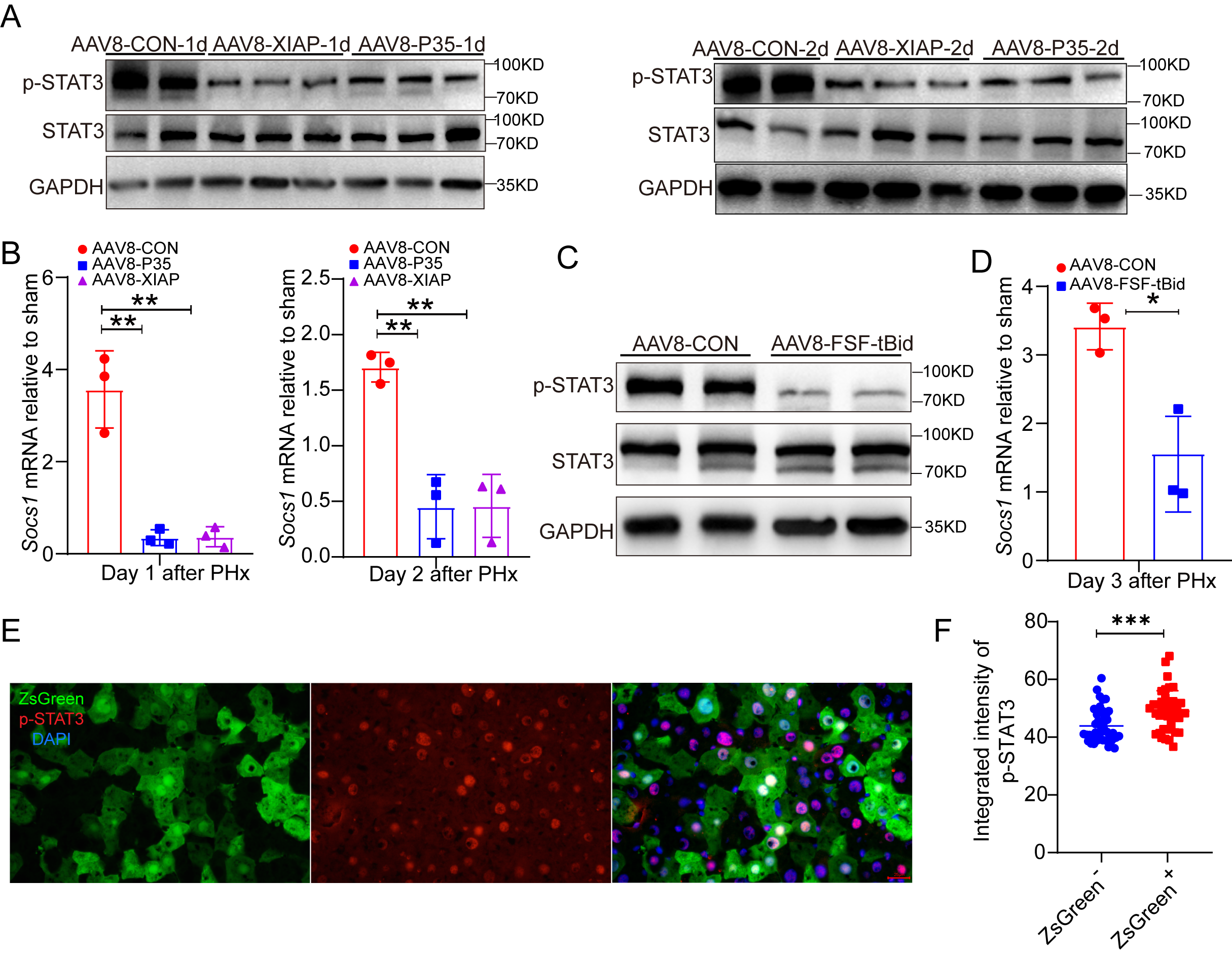

Supplement: S15 Fig — (A) Western blots showing the protein levels of p-STAT3 and STAT3 in livers with the indicated AAV8 on day 1 and 2 after PHx. (B) The mRNA level of Socs1 in the indicated groups on day 1 and 2 after PHx. The level in the sham-operated animals was set as 1. Three mice per group. (C, D) The protein levels of p-STAT3 and STAT3 (C) and the mRNA level of Socs1 (D) in livers with the indicated AAV8 on day 3 after PHx. Three mice per group. (E) The representative images of p-STAT3 staining in Sox2-Cre; mCasExpress livers on day 2 after PHx. Scale bar: 25 μm. (F) Quantification of the integrated intensity of nuclear p-STAT3 in ZsGreen+ cells and ZsGreen− cells in Sox2-Cre; mCasExpress livers. Five mice were included and about 8 fields were quantified per mouse. Each point in the graph represents the average intensity of p-STAT3 in cells in one field. Data are presented as the mean ± SD. *: P < 0.05. **: P < 0.01. ***: P < 0.001. The data underlying the graphs shown in the figure can be found in S1 Data. Raw blot images can be found in S1 Raw Images. (TIF) [file pbio.3003357.s015.tif]

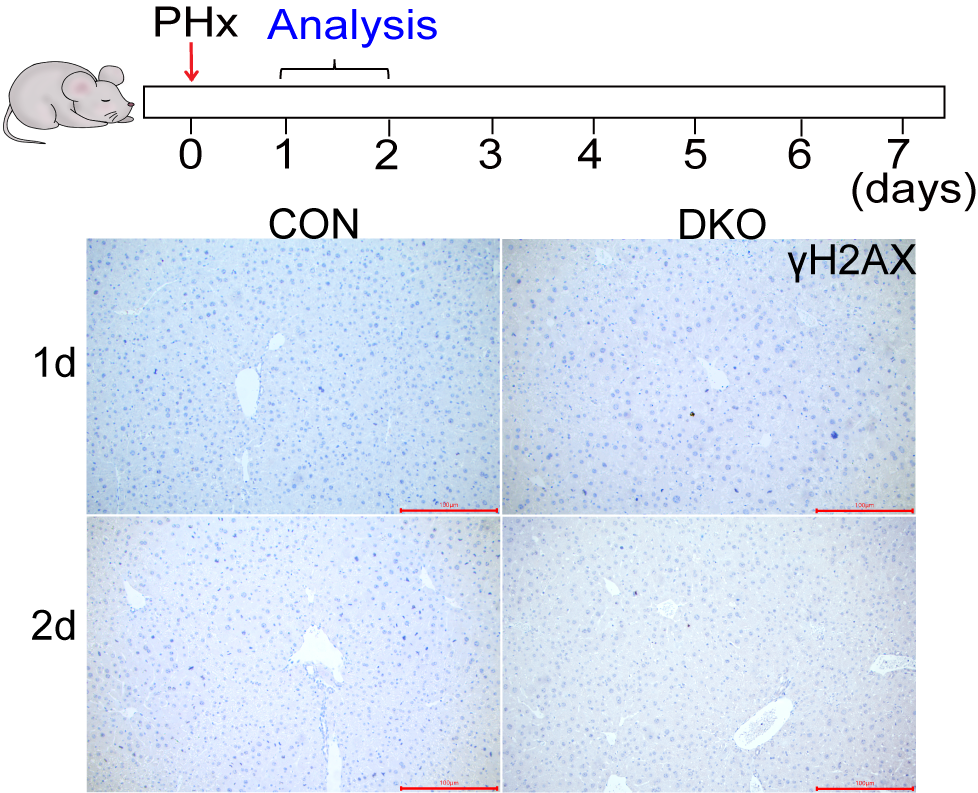

Supplement: S16 Fig — Scale bar: 100 μm. (TIF) [file pbio.3003357.s016.tif]

FIG1-G

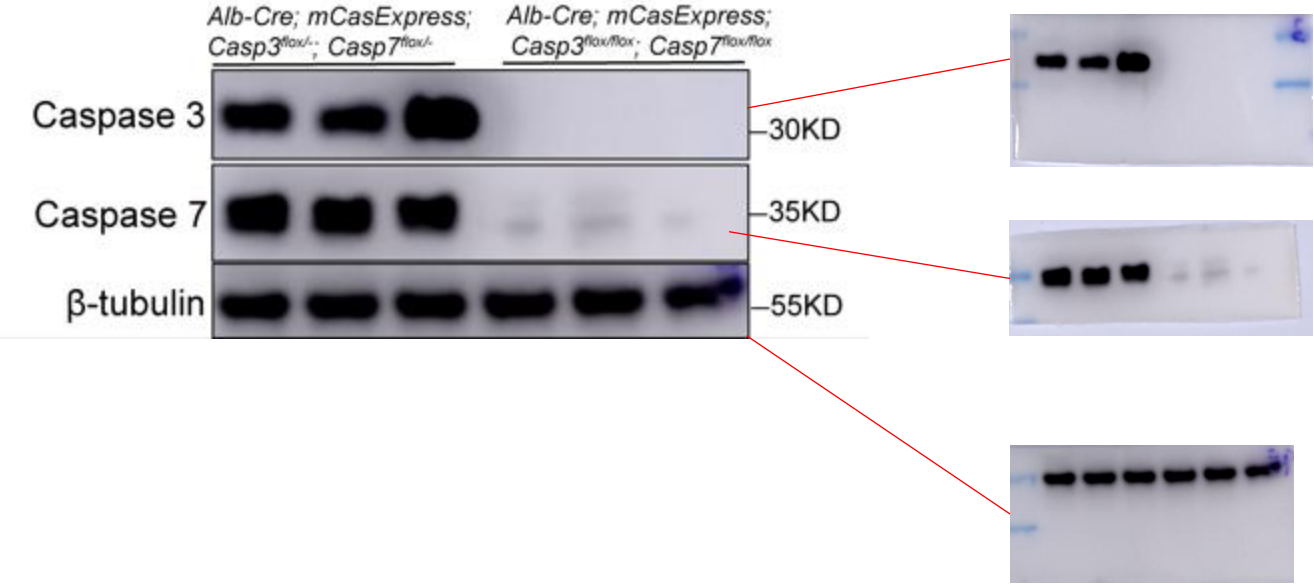

FIG3-H

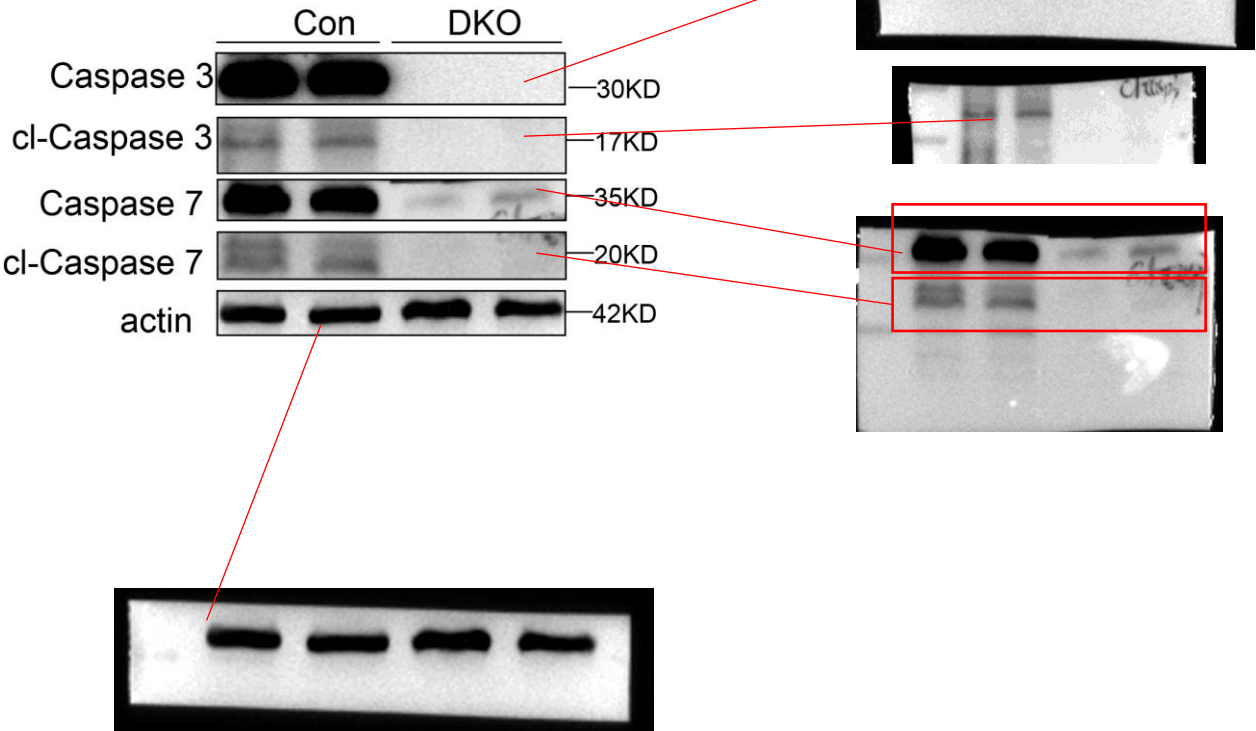

FIG5-G

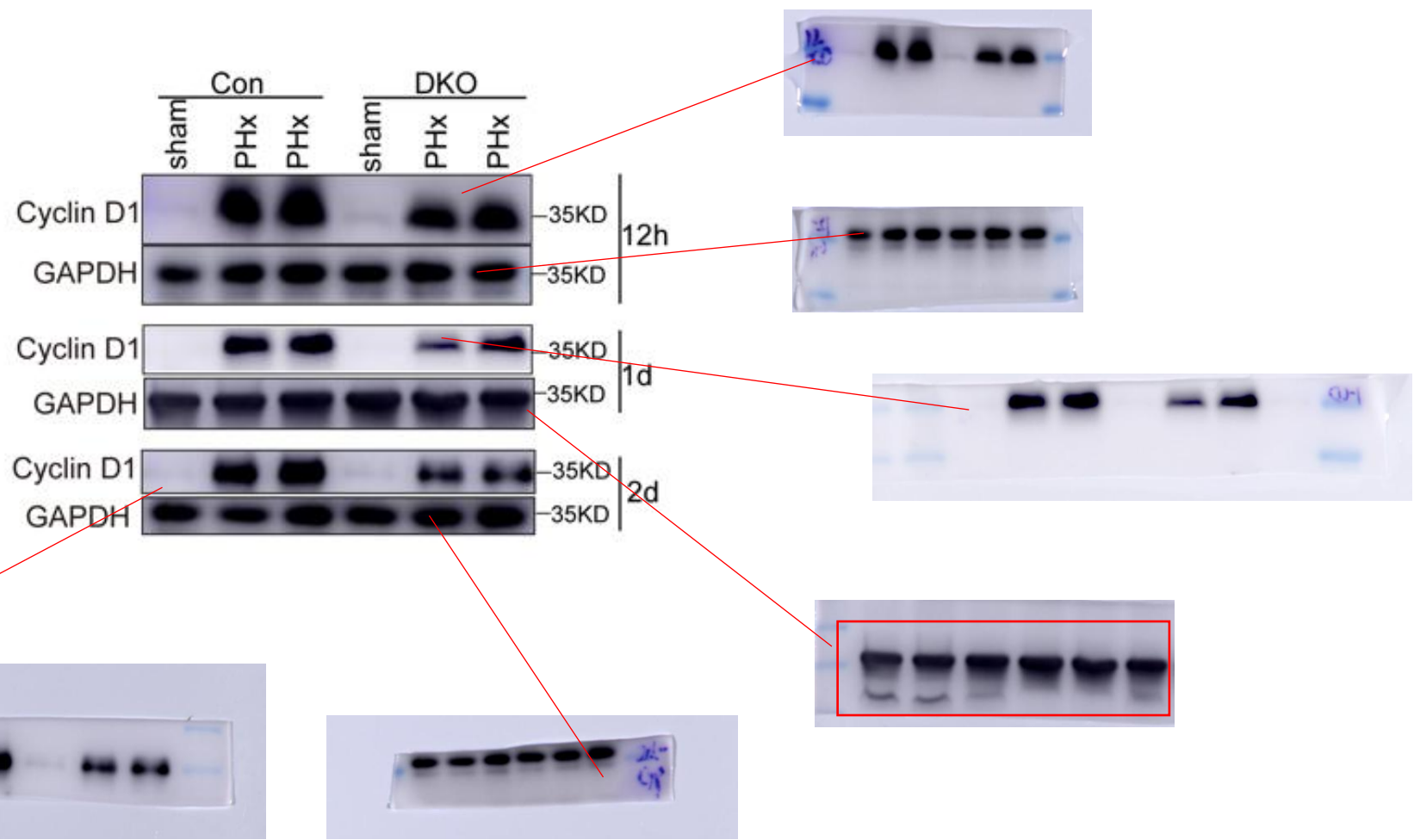

FIG6-D

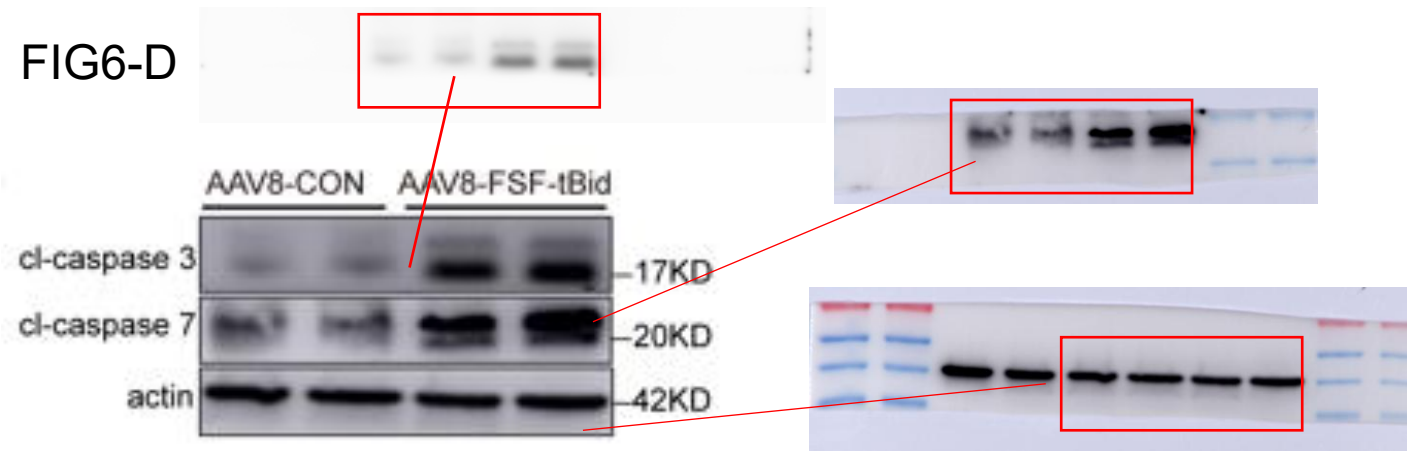

FIG6-M

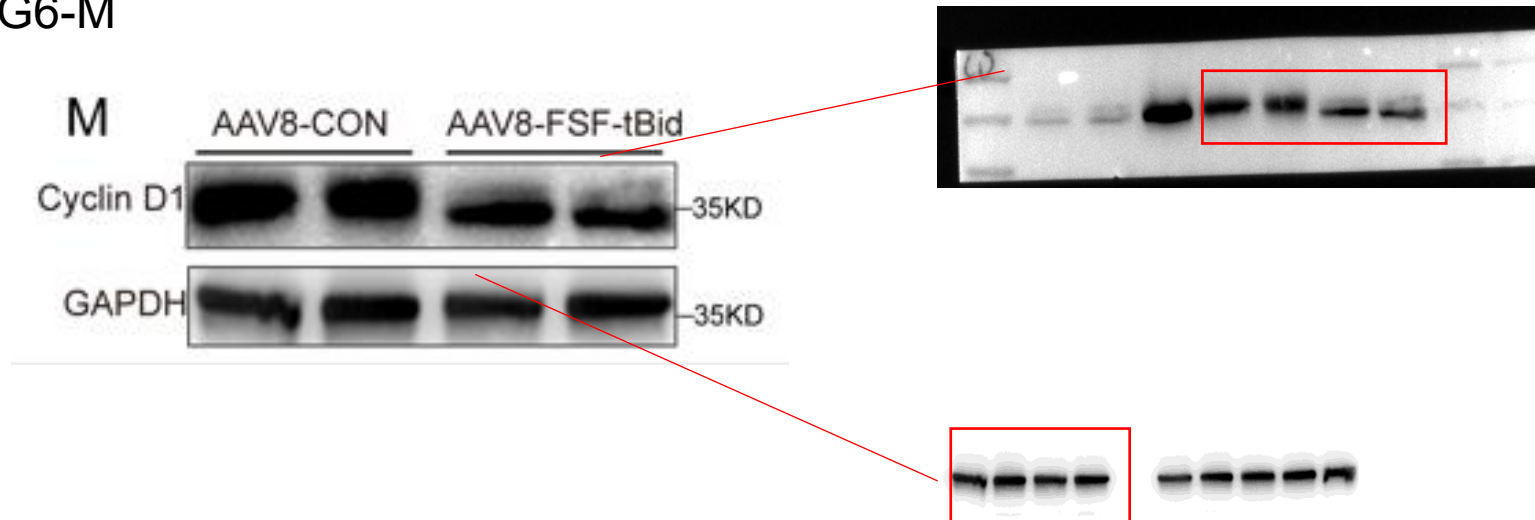

FIG7-A-1

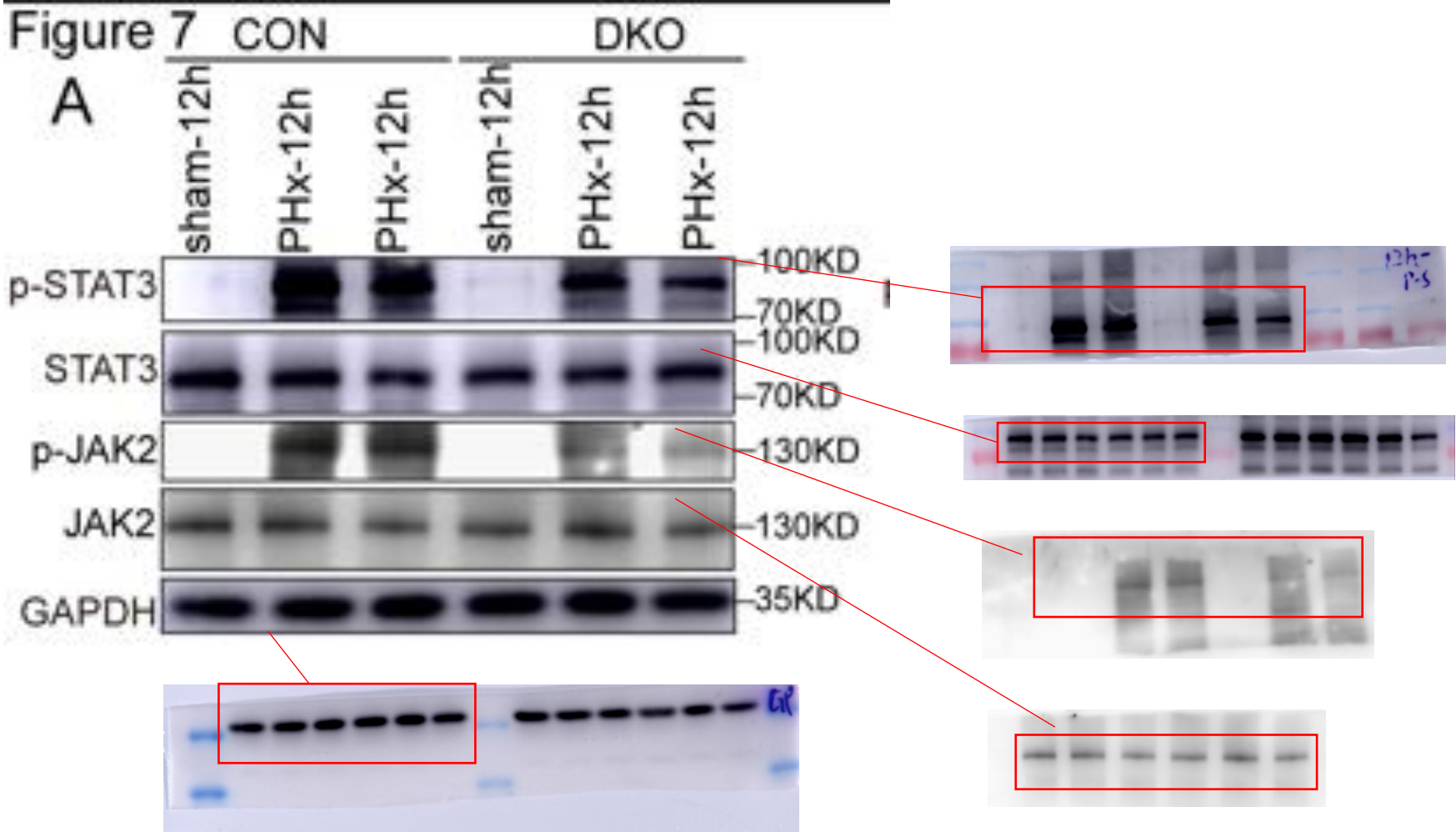

FIG7-A-2

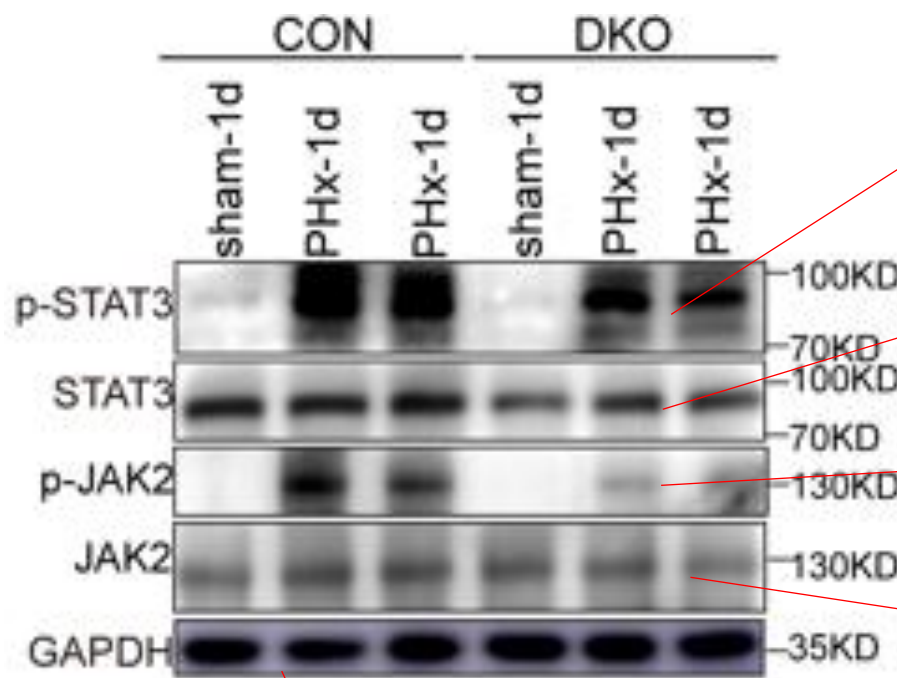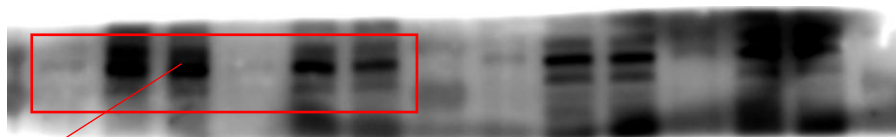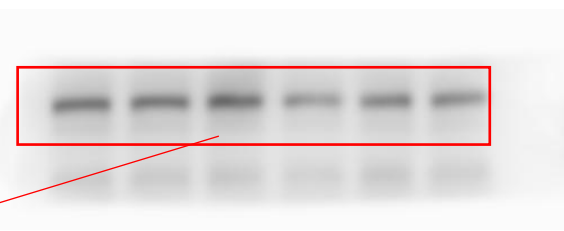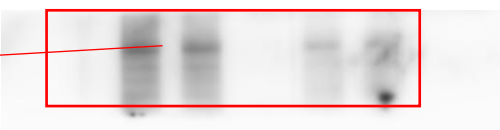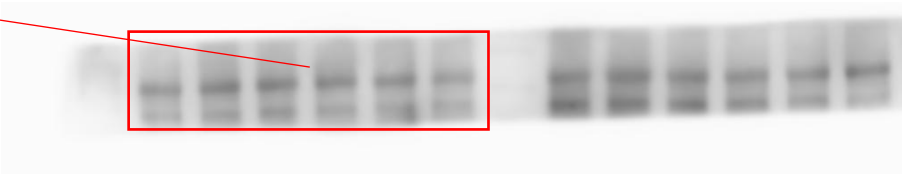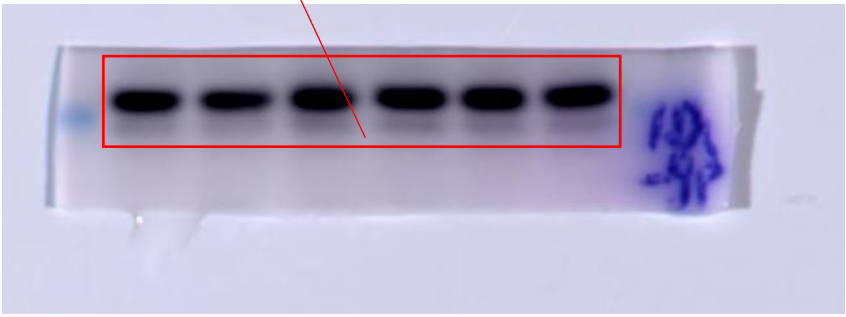

FIG7-A-3

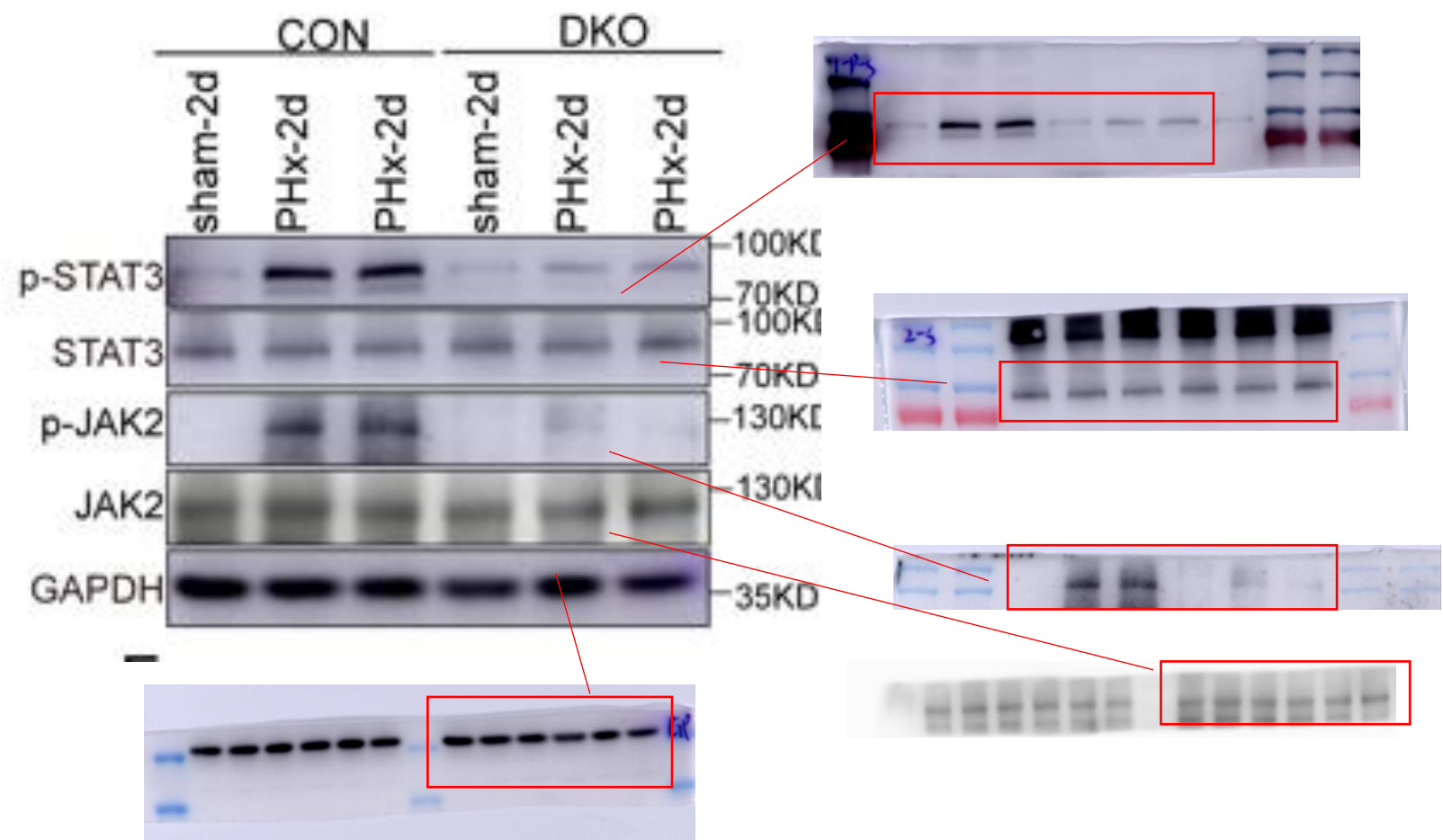

FIG7-F

F

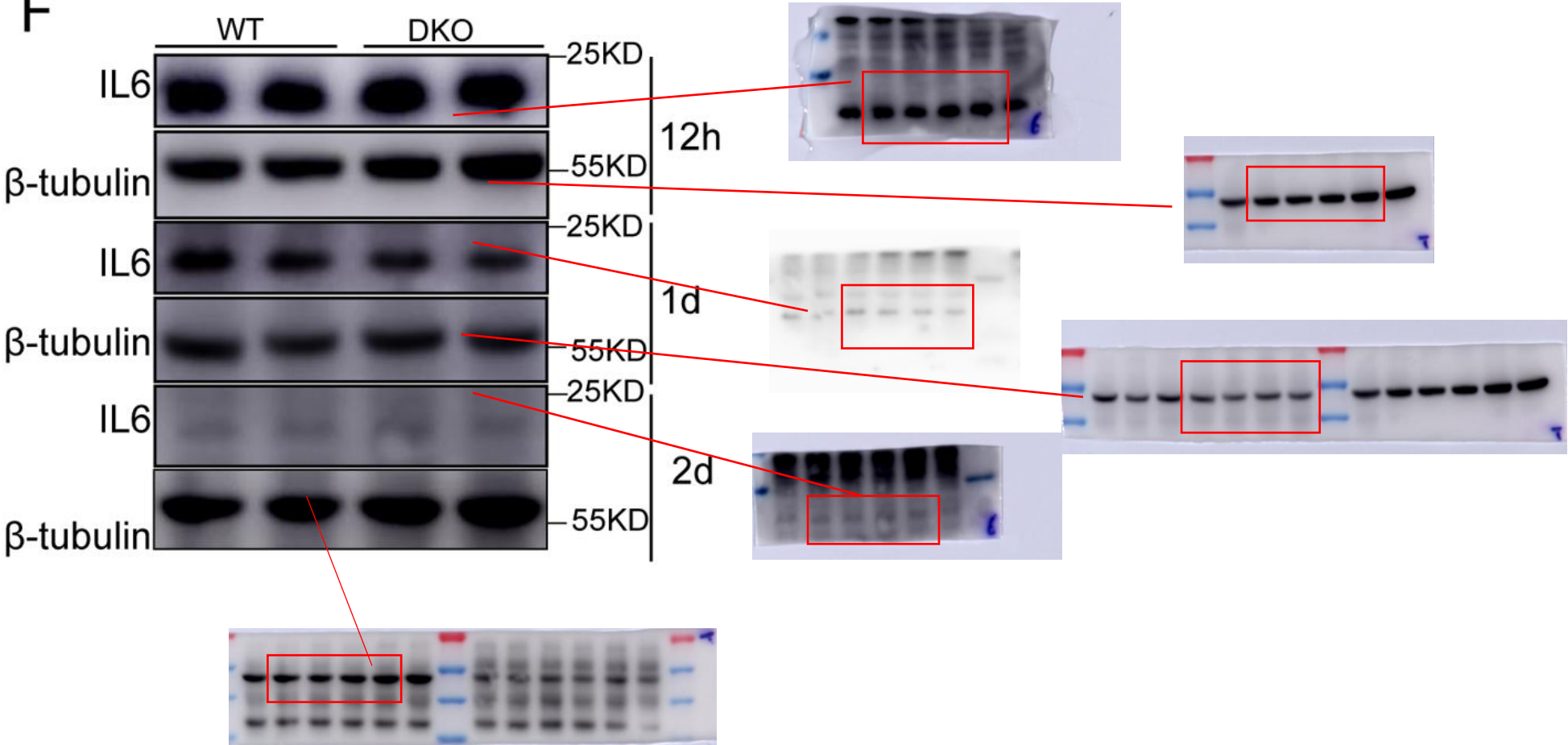

FIG7-G

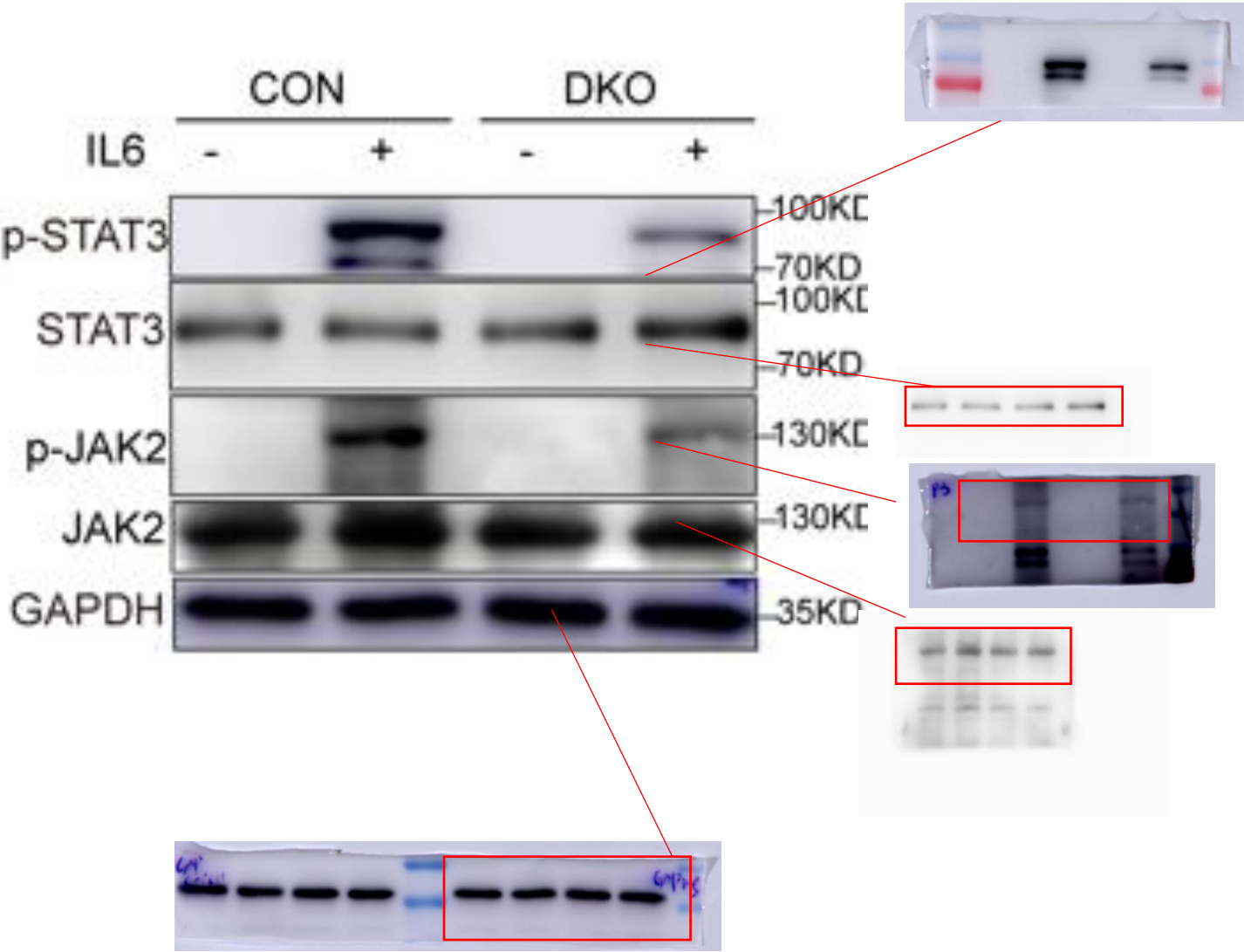

FIG7-I

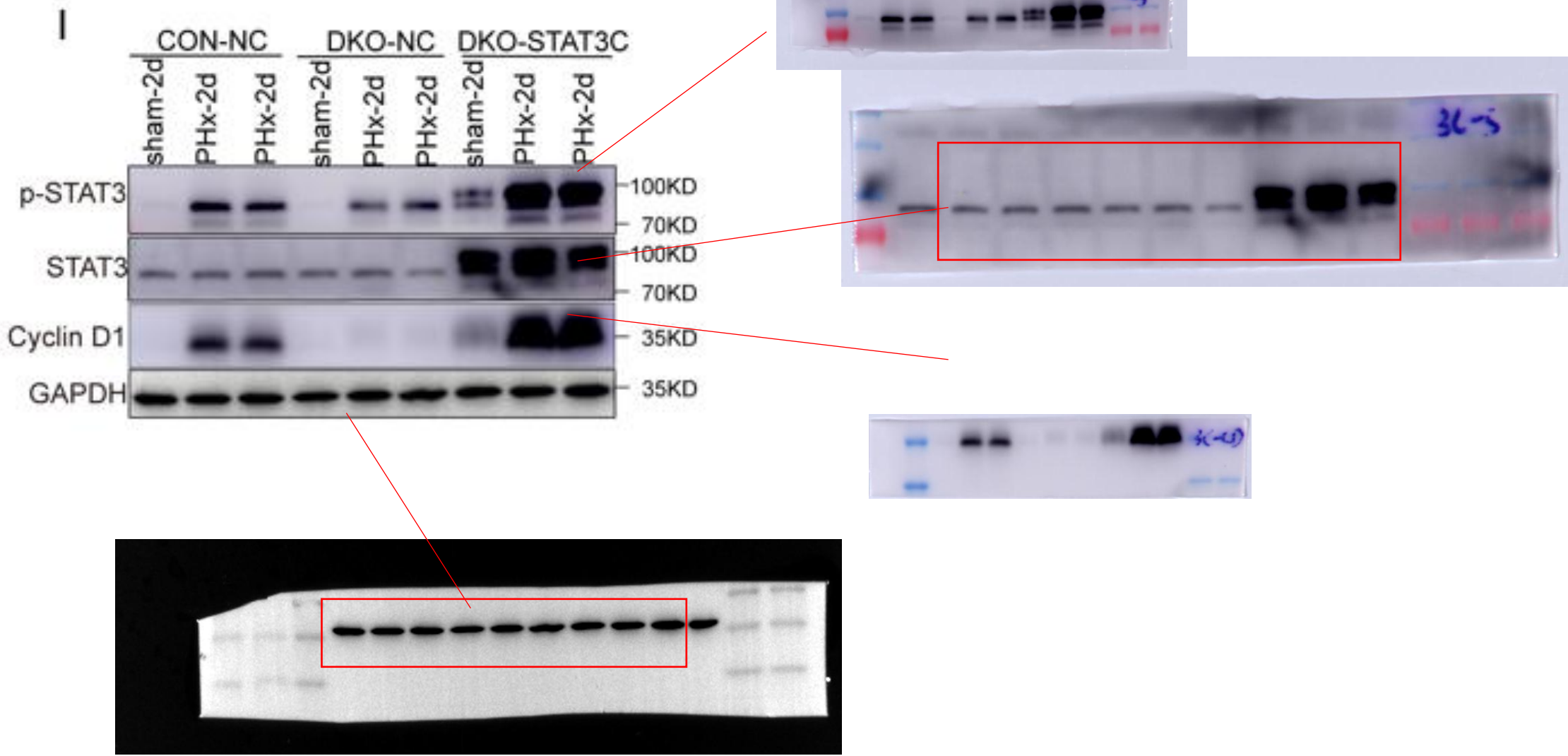

FIG S4-D

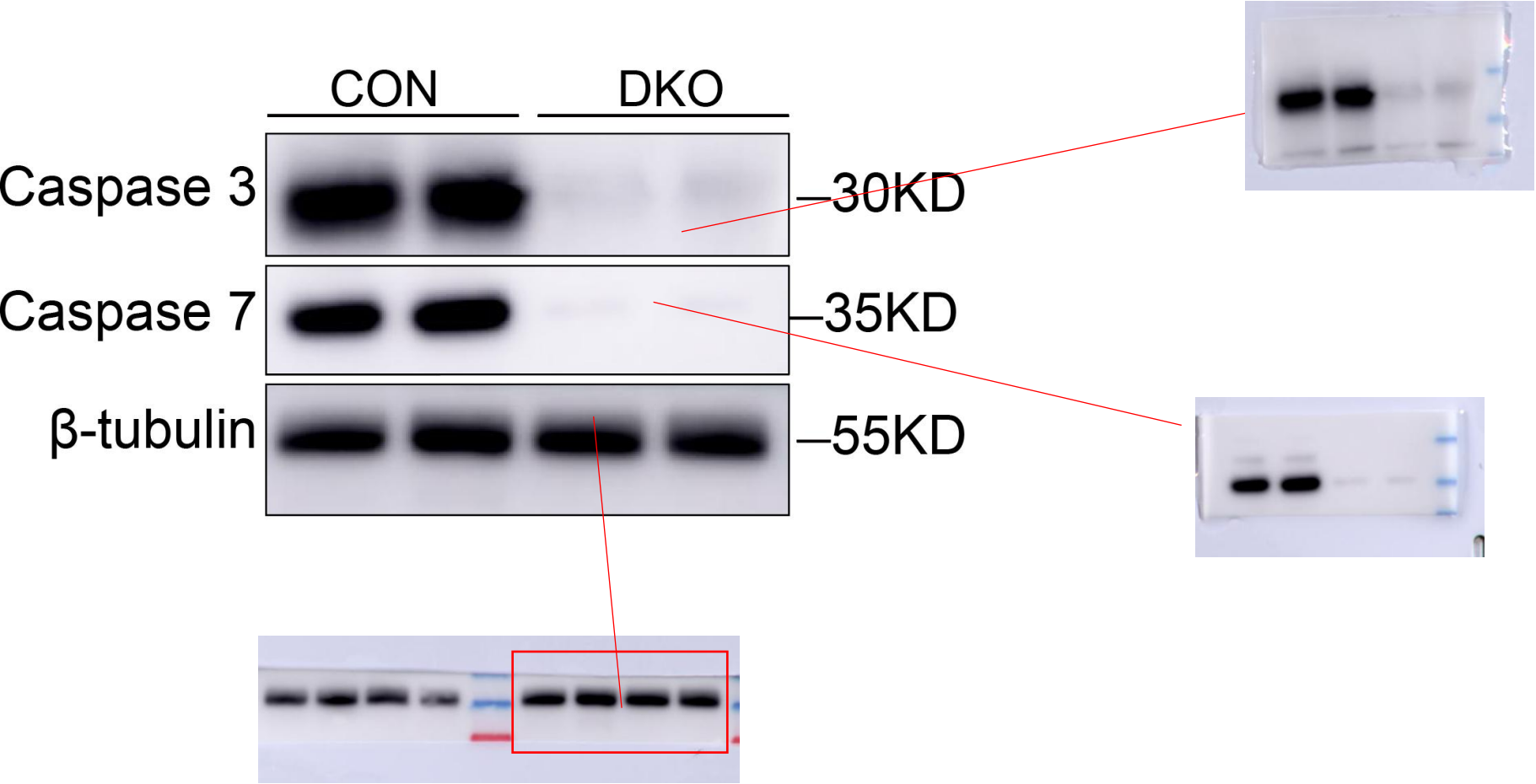

Fig-S11

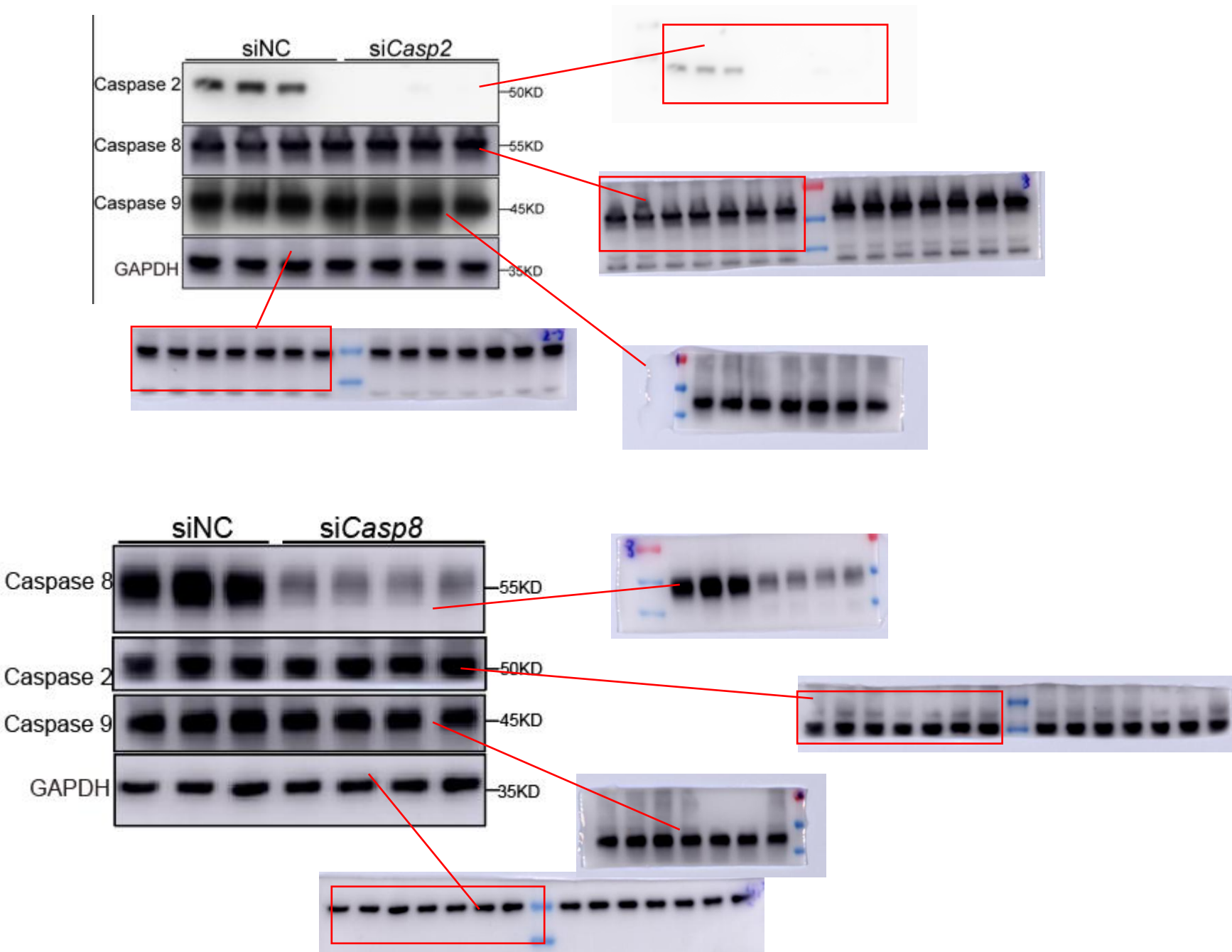

FIG-S11

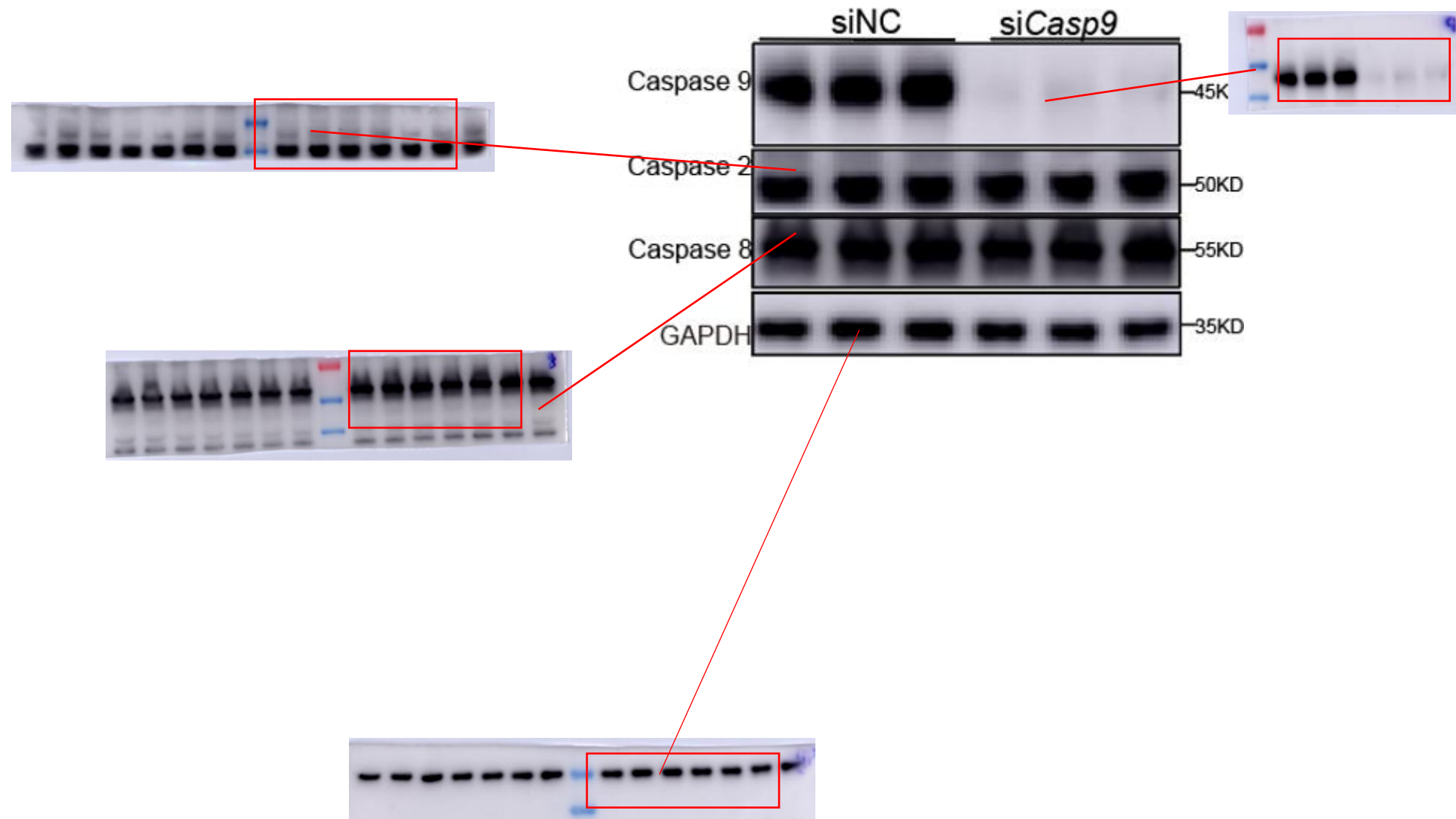

FIGS12-G

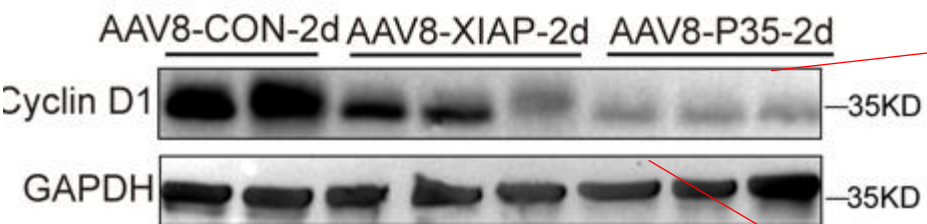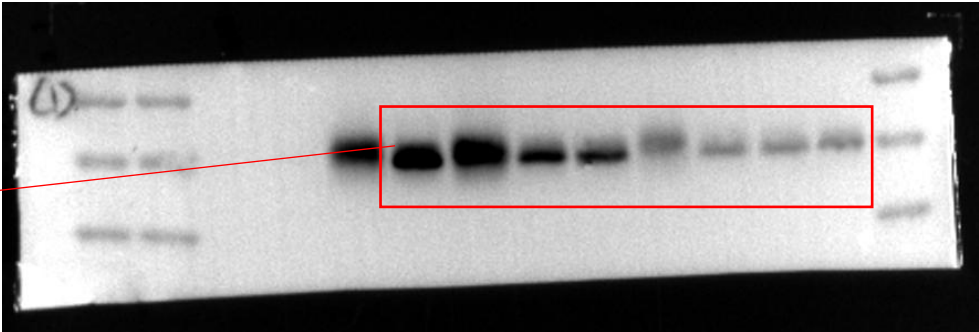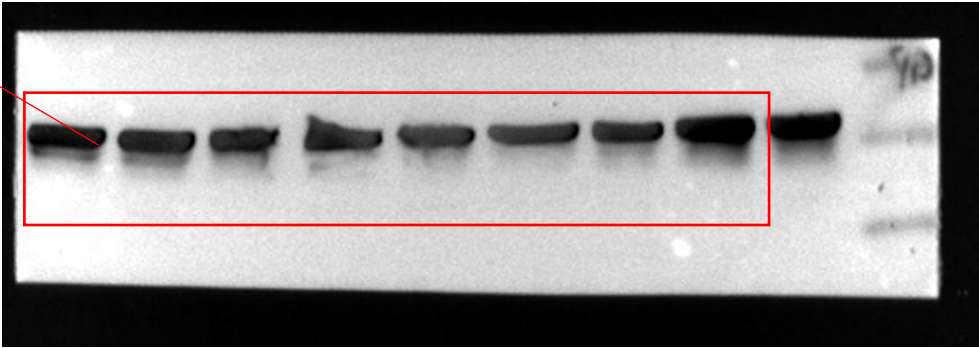

FIGS15-  
A-1

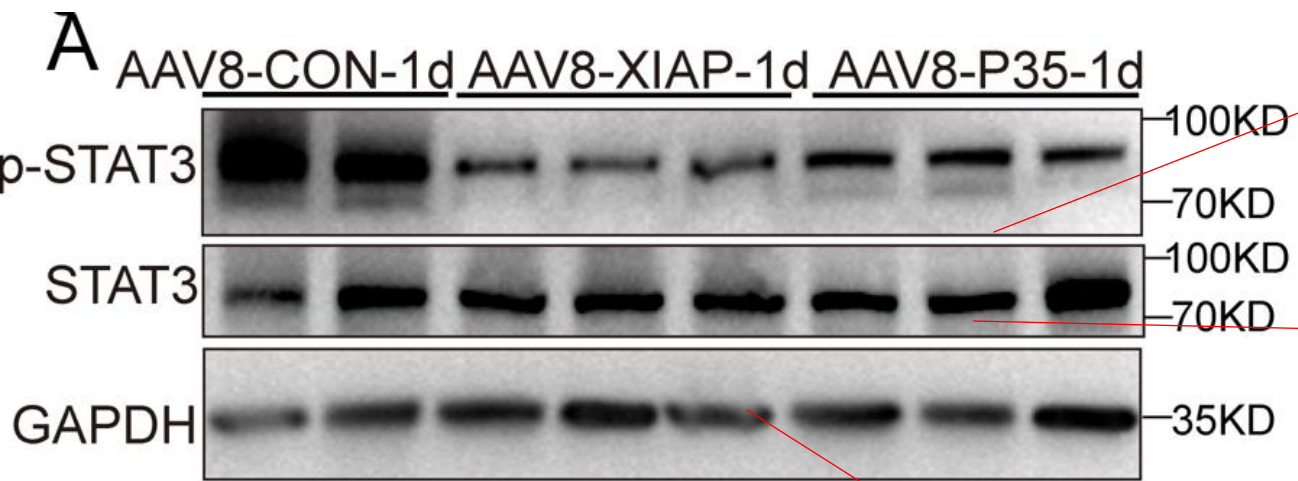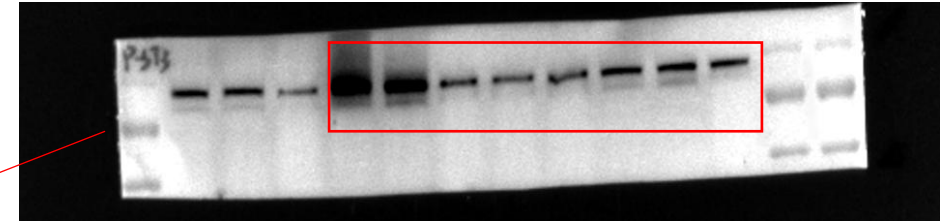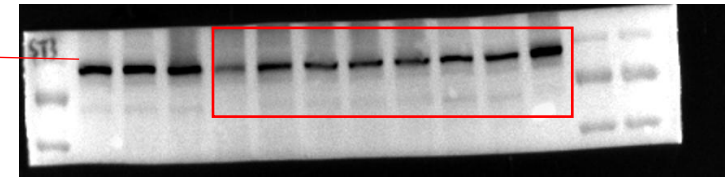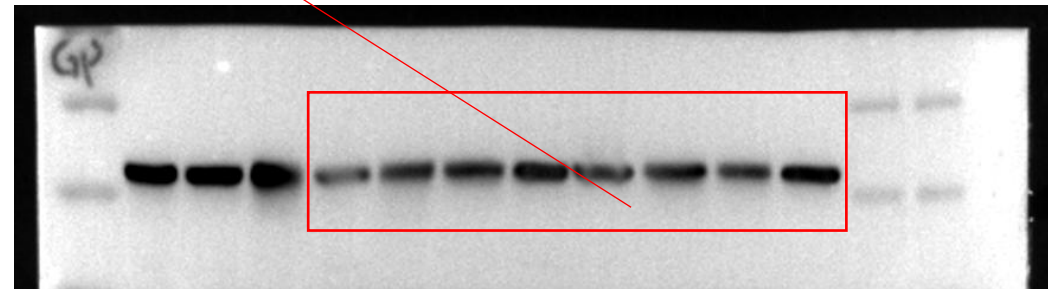

FIGS15-  
A-2

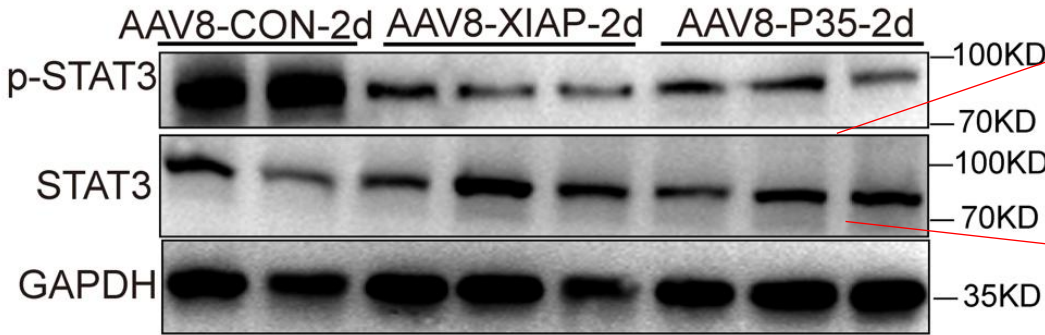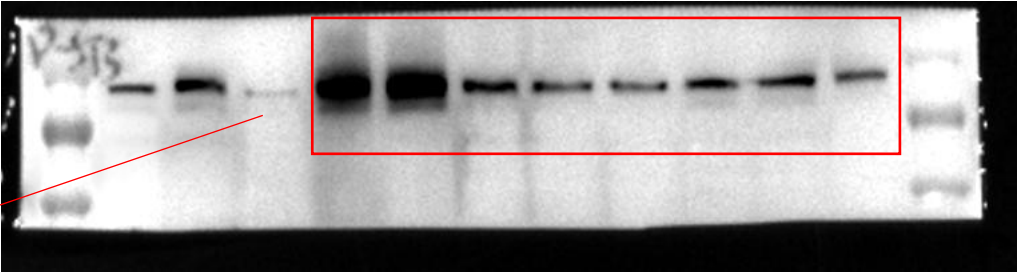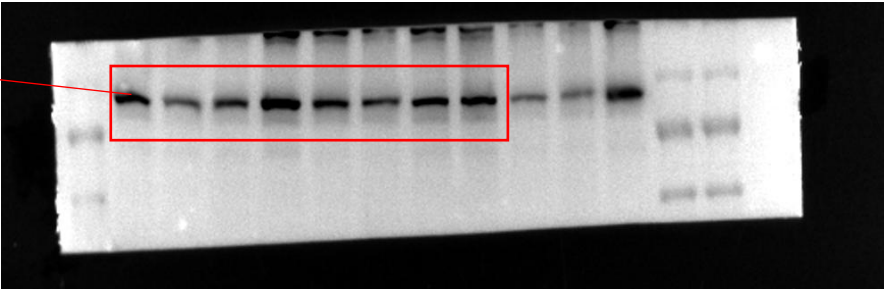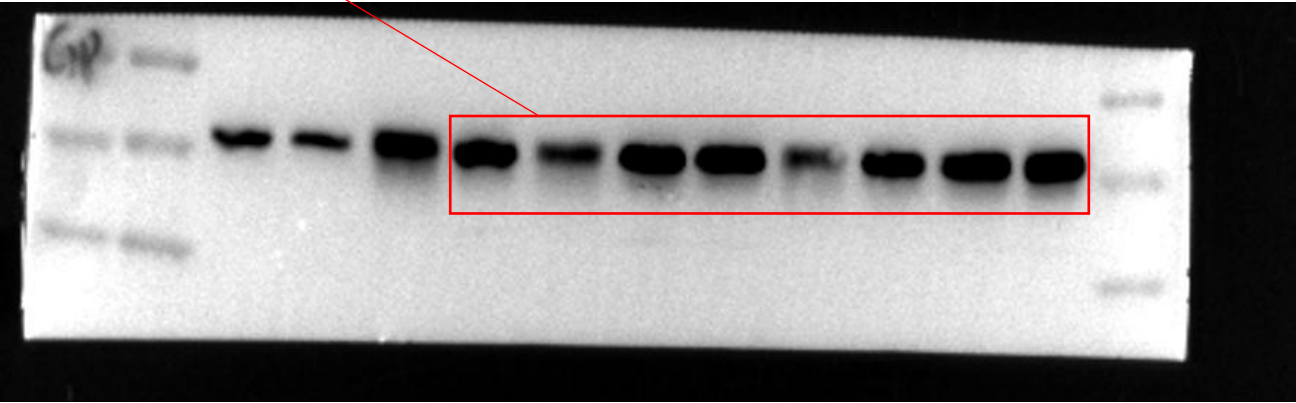

FIGS15-C

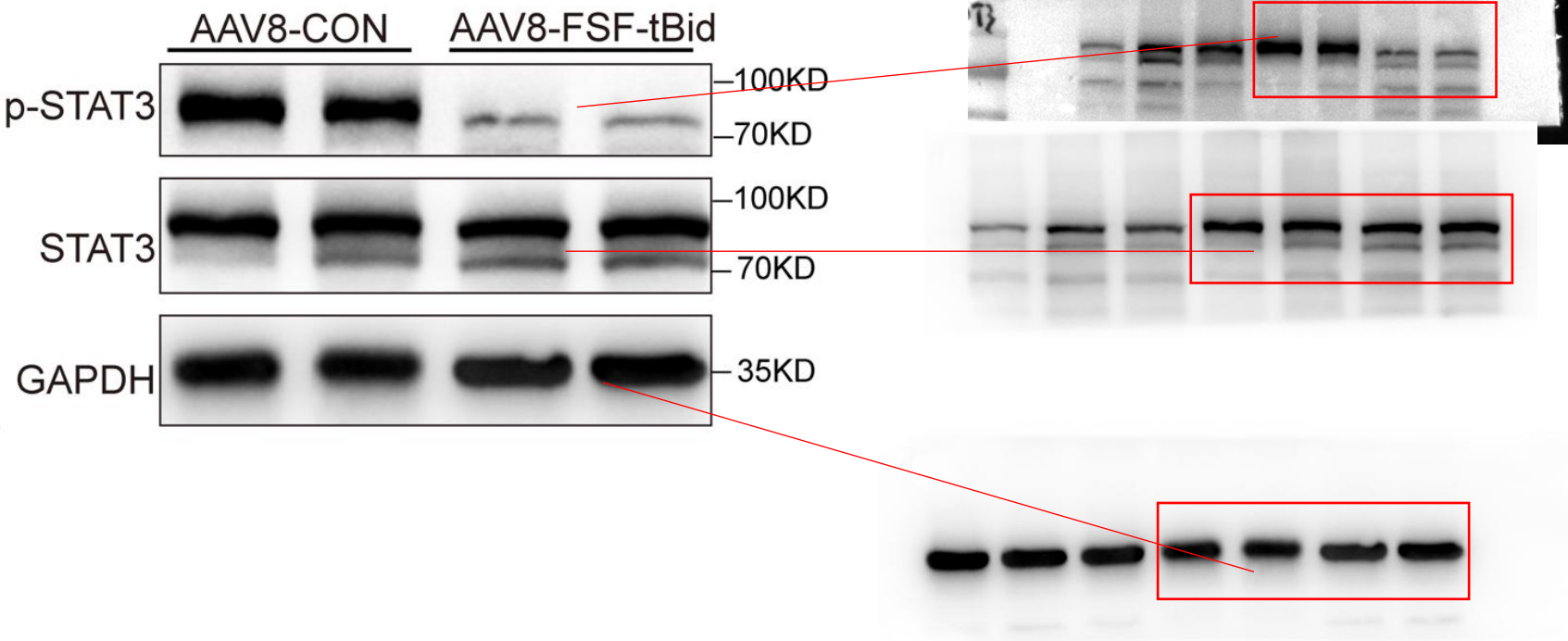

Supplement: S1 Raw Images — (PDF) [file pbio.3003357.s020.pdf]
