# Supplementary material for: Sublethal executioner caspase activation in hepatocytes promotes liver regeneration through the JAK/STAT3 pathway
Source: PLoS Biol. 2025 Aug 28;23(8):e3003357. doi: 10.1371/journal.pbio.3003357 (PMC12407553; doi:10.1371/journal.pbio.3003357)
Supplement: S1 Text — (DOCX) [file pbio.3003357.s017.docx]

**The list of oligonucleotides used in this study.**

| **qPCR** | | |
| --- | --- | --- |
| **Gene name** | **Forward primer** | **Reverse primer** |
| *Ccnd1* | AGGCGGATGAGAACAAGCAG | AGAAAGTGCGTTGTGCGGTA |
| *Ccne1* | CCTTTCAGTCCGCTCCAGAA | GGATGAAAGAGCAGGGGTCC |
| *Socs1* | CTGCGGCTTCTATTGGGGAC | AAAAGGCAGTCGAAGGTCTCG |
| *FLP* | TGGTGTACCTGGACGAGTTCCTG | CTGCTTGTTGCTGCTGCTGTTG |
| *XIAP* | AGCATTTGGGAGGCAGGGGTAG | GCTTGGGCTAGTGCGCTCAG |
| *P35* | TGGCAGCGTGTTGAAAAGCAAG | GCATACCGCCACGTAGCAGTC |
| *tBid* | AAGACAGCCTTCCCCAGAGA | CGTGTGGAAGACATCACGGA |
| *Actin* | GTGCTATGTTGCTCTAGACTTCG | ATGCCACAGGATTCCATACC |
| **Genotyping** | | |
| **Gene name** | **Forward primer** | **Reverse primer** |
| *LN-DEVD-FLP* | ATCATCCCTTACAACGGCCA | CCGGTCCTGTTCACTCTCTT |
| *FSF-ZsGreen* | AGATGACCATGAAGTACCGCA | CTCCCAGTTGTCGGTCATCT |
| *Sox2-Cre* | CCCGCAGAACCTGAAGATG | GACCCGGCAAAACAGGTAG |
| *CAG-Cre* | TTCGGCTTCTGGCGTGTGA | CTGACTTCATCAGAGGTGGCATC |
| *Alb-Cre* | TGGATGCCACCTCTGATGAAGTC | TCCTGGCATCTGTCAGAGTTCTCC |
| *Alb-Cre WT* | CAGCAAAACCTGGCTGTGGATC | ATGAGCCACCATGTGGGTGTC |
| *tetO-Cre* | GCGGTCTGGCAGTAAAAA CTA TC | GTGAAACAGCATTGCTGTCAC TT |
| *tdTomato* | CGGCATGGACGAGCTGTACAAG | TCAGCAAACACAGTGCACACCAC |
| *tdTomato WT* | CCCAAAGTCGCTCTGAGTTGTTA | TCGGGTGAGCATGTCTTTAATCT |
| *Casp3^flox^* | GCATCGCATTGTCTGAGTAGGTG | TACTTGGTCCCGAGTAAGTGGAAC |
| *Casp3 WT* | CAGCAAAACCTGGCTGTGGATC | ATGAGCCACCATGTGGGTGTC |
| *Casp7^flox^* | CATGAAAGGTCTGGGATTGTG | CCGCCCCTGTATGTTTTG |
| *rtTA* | AAAGTCGCTCTGAGTTGTTAT | GCGAAGAGTTTGTCCTCAACC |
| *rtTA WT* | AAAGTCGCTCTGAGTTGTTAT | GGAGCGGGAGAAATGGATATG |
| *H11* | CAGCAAAACCTGGCTGTGGATC | ATGAGCCACCATGTGGGTGTC |
| *ROSA26* | CCCAAAGTCGCTCTGAGTTGTTA | TCGGGTGAGCATGTCTTTAATCT |
| **siRNA** | | |
| **Name** | **sense** | **Anti-sense** |
| siNC | UUCUCCGAACGUGUCACGUTT | ACGUGACACGUUCGGAGAATT |
| si*Casp2* | CUACAAUGUCCAUGUGCUATT | UAGCACAUGGACAUUGUAGTT |
| si*Casp8* | GCGCAGACCACAAGAACAATT | UUGUUCUUGUGGUCUGCGCTT |
| si*Casp9* | CAAUGCGACUCACAGCAAATT | UUUGCUGUGAGUCCAUUGTT |
