# Supplementary material for: Sublethal executioner caspase activation in hepatocytes promotes liver regeneration through the JAK/STAT3 pathway
Source: PLoS Biol. 2025 Aug 28;23(8):e3003357. doi: 10.1371/journal.pbio.3003357 (PMC12407553; doi:10.1371/journal.pbio.3003357)
Supplement: S2 Text — (DOCX) [file pbio.3003357.s018.docx]

**The list of antibodies.**

| **Antibody** | **Company** | **Catalog No.** | **Working dilution** |
| --- | --- | --- | --- |
| Anti-HNF4α | Cell Signaling Technology | 3113 | 1:200 (IF) |
| Anti-CK19 | Servicebio | GB12197 | 1:200 (IF) |
| Anti-CD31 | Servicebio | GB11315 | 1:200 (IF) |
| Anti-CD68 | Cell Signaling Technology | 97778 | 1:200 (IF) |
| Anti-Glutamine Synthetase | Abcam | Ab176562 | 1:500 (IF) |
| Anti-Ki67 | Abcam | Ab15580 | 1:200 (IF) |
| Anti-Ki67 | Cell Signaling Technology | 12202 | 1:200 (IHC) |
| Anti-GAPDH | Proteintech | 60004-1-1g | 1:5000 (WB) |
| Anti-IL6 | ABclonal | A11115 | 1:1000 (WB) |
| Anti-STAT3 | Cell Signaling Technology | 9139 | 1:1000 (WB) |
| Anti-phosphor-STAT3 | Cell Signaling Technology | 9145 | 1:1000 (WB)  1:200 (IF) |
| Anti-Cyclin D1 | Abways | CY5404 | 1:1000 (WB) |
| Anti-JAK2 | Santa Cruz Biotechnology | sc-390539 | 1:1000 (WB) |
| Anti-phosphor-JAK2 | Cell Signaling Technology | 3771 | 1:1000 (WB) |
| Caspase 2 | ABclonal | A5724 | 1:1000 (WB) |
| Caspase 8 | ABclonal | A11324 | 1:1000 (WB) |
| Caspase 9 | ABclonal | A18676 | 1:1000 (WB) |
| Caspase 3 | Cell Signaling Technology | 14220 | 1:1000 (WB) |
| cl-Caspase 3 | Cell Signaling Technology | 9664 | 1:1000 (WB) |
| Caspase 7 | Cell Signaling Technology | 9492 | 1:1000 (WB) |
| Anti-Tubulin | NCM Biotech | AB2002 | 1:2000 (WB) |
| Anti-actin | Proteintech | 66009-1-1g | 1:2000 (WB) |
| 568- goat anti-rabbit | Invitrogen | A11011 | 1:200 (IF) |
| 488- goat anti-rabbit | Invitrogen | A11029 | 1:200 (IF) |
| 488- goat anti-mouse | Invitrogen | A11028 | 1:200 (IF) |
| HRP-goat anti-mouse | ORIGENE | ZB-2305 | 1:5000 (WB) |
| HRP-goat anti-rabbit | ORIGENE | ZB-2301 | 1:5000 (WB) |

IF: immunofluorescence; IHC: immunohistochemistry; WB: Western blotting
